# Network pharmacological investigation and experimental verification of the peel of *Zea mays* L. regulating metabolic reprogramming in the treatment of diabetic nephropathy

**DOI:** 10.3389/fendo.2025.1594782

**Published:** 2025-07-30

**Authors:** Andong Wang, Yuru Yang, Yaonan He, Guangtong Chen, Bai Ling, Xiaotian Cheng

**Affiliations:** ^1^ School of Pharmacy, Nantong University, Nantong, Jiangsu, China; ^2^ Department of Pharmacy, The Yancheng Clinical College of Xuzhou Medical University & The First People’s Hospital of Yancheng, Yancheng, Jiangsu, China

**Keywords:** diabetic nephropathy, the peel of *Zea mays* L., network pharmacology, metabolic reprogramming, experimental verification

## Abstract

**Background:**

*Zea mays* L. is one of the most significant genes in the Gramineae family, and the peel of *Zea mays* L. (YMP), an unproven folk remedy for diabetes, has not been well studied. Diabetic nephropathy (DN) is one of the most well-known and dangerous microvascular effects of diabetes mellitus. The effects and mechanisms of YMP on metabolic reprogramming are largely unknown.

**Methods:**

The components of YMP were systematically identified using UPLC-Q-TOF-MS/MS. A network pharmacology study between DN and significant components was then carried out. The pharmacological trials of YMP were evaluated in mice with diabetes. *In vitro* measurements were made of the biochemical activity, anti-inflammatory, and antioxidant properties. Moreover, UHPLC-LTQ-Orbitrap MS was used to do investigations on the metabolomics of serum and urine. Ultimately, transcriptomics analysis was utilized to clarify the complex processes by which the transcription factor influences DN.

**Results:**

43 components were systematically identified from YMP. It was found by network pharmacology analysis that signal transduction, namely metabolic disruption, involved pathways with a high degree of engagement. Experimental verification showed that YMP administration increased glomerular hypertrophy, collagenous tissue proliferation, urine microalbumin/creatinine ratio, inflammatory response remission, and oxidative stress promotion *in vivo*. Treatment with YMP may affect the pathways that are involved in the metabolism of amino acids and energy, as well as reverse metabolite abnormalities. YMP has the ability to restore the levels of metabolites like Gluconolactone, *D*-Ribulose 5-phosphate, Xylulose 5-phosphate, *L*-Alanine, *L*-Aspartic acid, Glutamic acid, Citrulline, *L*-Arginine, *L*-Leucine, *L*-Valine, *L*-Isoleucine, and so on. Metabolic reprogramming of energy metabolism was demonstrated. By transcriptomics, when STZ is administered, the GPI, GAPDH, G6PC, HK2, HK1, and HK3 genes associated with glycolysis/gluconeogenesis were significantly elevated from the model groups. However, the pentose phosphate pathway-related genes G6PD, PGLS, RPE, TALDO1, and HXLB significantly elevated when YMP was administered.

**Conclusions:**

This study was the first to show that YMP corrected disruptions in the pentose phosphate pathway and amino acid metabolism, alleviated diabetes-induced pathological changes in the kidneys of diabetic mice, and had a regulating effect on the liver glycolipid metabolism. By investigating the novel pharmacological effect of traditional Chinese medicine and encouraging in-depth study and development, this work may offer a new experimental foundation and theoretical direction for the sensible application of YMP on DN.

## 1 Introduction

The defining feature of diabetes mellitus was elevated plasma glucose levels. Diabetes was emerging as a significant chronic noncommunicable disease that was threatening people’s health, along with cancer and cardiovascular disorders (1). Nephropathy, neuropathy, and retinopathy were among the problems associated with diabetes. DN was one of the most prevalent and widespread long-term consequences of diabetes (2). Furthermore, DN was the leading cause of end-stage renal disease in developed countries. Additionally, there was a significant chance that DN might exacerbate cardiovascular illness (3). Consequently, DN significantly hindered public health. To date, no specific approach has proven effective in controlling the course of DN (4). No new drugs have been developed to treat DN in the last 20 years, and its pathogenic mechanism was still a mystery. Thus, it was essential to comprehend the pathogenic mechanism of DN and create new treatment drugs (5). Proteinuria was one of the most important biomarkers for diagnosing chronic renal disease and predicting its prognosis. The DN stage was determined by the degree of proteinuria (6). The diabetes mouse model’s progression was quite similar to that of people, and microvascular problems, particularly DN, were frequent.

*Zea* is one of the most significant genes in the Gramineae family, which has about 10,000 species (7). The material that makes up corn husk was brought to China during the Ming Dynasty. It is thought to be a diuretic that can treat edema, urinary stones, unpleasant urination, and appetite loss. Acute and chronic nephritis, edema, proteinuria, and diabetes are among the conditions for which corn is listed as a medicine in the “Dian nan Materia Medica”, “Ling nan Caiyao Lu”, “Modern Practical Chinese Medicine”, “Dictionary of Traditional Chinese Medicine”, and numerous other Chinese medical texts (8). Corn husks received less attention in earlier research than corn silk.

Network pharmacology is a new method of drug discovery that relates drugs to disease-related targets and gives a general notion of how they function. From a comprehensive perspective, it may create a biological network of connections and predict the analysis of related targets and pathways with high accuracy (9). Pharmacological therapy’s basic mechanisms were elucidated through the use of several targets and pathways. An integrated method for identifying possible medicinal medicines and their underlying mechanisms of action is network pharmacology. The molecular processes underlying drug therapy may be better understood thanks to network pharmacology (10). Over the past few decades, metabolomics has become increasingly popular in clinical and biological research thanks to advanced analytical techniques and bioinformatics (11). Metabolomics may be used to identify biomarkers linked to disease processes, identify changes in metabolites linked to disease, and identify biomarkers in epidemiology (12). In order to determine the pathogenesis of the disease and assess the drug’s mode of action, information technology is currently being used to identify landmark metabolites in organs, cells, and excretion (12).

Understanding the fundamentals and processes by which gene activity coordinates intricate cellular configurations in multicellular animals will have a significant impact on life science research. Recent developments in next-generation sequencing and imaging-based techniques have demonstrated the ability of spatial transcriptomics to systematically measure the expression levels of all or most genes throughout tissue space. This technology has been used to investigate a variety of disease contexts, including cancer, and to produce biological insights in the fields of neuroscience, development, and plant biology (13). Rosmarinic acid (RA) attenuated the cytotoxicity of natural killer cells, oxidative stress, and the inflammatory response of macrophages while significantly reducing renal tubular epithelial injury, especially in the proximal tubular S1 segment and on glomerular epithelial cells called podocytes. These results offer a thorough comprehension of the ways in which RA reduces inflammation, oxidative stress, and kidney damage, providing important direction for the practical use of RA in the management of DN (14). We generated 23,980 single-nucleus transcriptomes from three normal and three early diabetic nephropathy samples using unbiased single-nucleus RNA sequencing (snRNA-seq) on cryopreserved human diabetic kidney samples. The final dataset included all of the kidney’s major cell types. Gene expression variations that are critical for ion transport, angiogenesis, and immune cell activation were shown to vary by cell type by side-by-side comparison. Specifically, we demonstrate that a gene expression signature related to increased potassium secretion is adopted by the diabetic thick ascending limb, late distal convoluted tubule, and principal cells. This includes changes in the expression of Na^+^/K^+^-ATPase, WNK1, mineralocorticoid receptor, and NEDD4L, as well as decreased paracellular calcium and magnesium reabsorption. Strong angiogenic signatures are also found in main cells, proximal and distal convoluted tubules, and glomerular cell types (15).

To provide a standard for evaluating quality, the pharmacological effects of YMP on DN were evaluated in diabetic mice. This work provided a method for determining the components of YMP. Metabolomics studies were conducted to examine the mechanism of action using serum and urine samples. *In vivo* metabolism was used to clarify how YMP affected the improvement of DN. As a result of these discoveries, an integrated network was created with the goal of identifying important endogenous differential metabolites, therapeutic elements, and related pathways. This work was expected to investigate the novel pharmacological action, promote further investigation and development, and provide a new experimental basis and theoretical guidance for the prudent use of YMP on DN.

## 2 Materials and methods

### 2.1 Extraction and screening ingredients of YMP

The complete YMP was collected and extracted using 95% EtOH in a reflux setup three times, lasting two hours each time. To create the 95% ethanolic extracts, the solvent was evaporated using a vacuum rotary evaporator. 50 mg of YMP powder were dissolved in 1.2 mL of 70% methanol in a centrifuge tube. Every 30 minutes, the mixture was vortexed six times for 30 seconds each. A 0.22 µm microporous membrane was used to filter the clear supernatant after the tube was centrifuged for three minutes at 12,000 rpm. After filtering, the solution was collected and put in an autosampler vial for a further qualitative UPLC-MS/MS analysis. The chromatographic separation was carried out using a SHIMADZU Nexera X2 UPLC system equipped with an Agilent SB C18 column (1.8 µm, 2.1 mm×100 mm). Ultra-pure water was used as mobile phase A, while chromatographic acetonitrile was used as mobile phase B. Both phases contained 0.1% formic acid. The gradient elution conditions were as follows: Over nine minutes, B increased from 5% to 95%, held for one minute, then dropped to 5% between 10 and 11 minutes, held for another minute. The column temperature was kept at 40°C, the injection volume was 4 μl, and the flow rate was set at 0.35 ml/min.

### 2.2 Establishment of animal model

Fifty (20 ± 2 g) female mice were supplied by Beijing HFK Bioscience Co., Ltd. (Beijing, China) and kept in cages with free access to food and water, 12 hours of light and dark cycles, and 25 ± 1°C and 55 ± 5% relative humidity. The animal protocol was reviewed and approved by the Nantong University Animal Ethical and Welfare Committee (R240411744), and on April 25, 2024, the animal ethics and welfare were approved using Approve No. S20240425-006. After a week of acclimatization, 10 mice were randomly chosen to form the control group (CON), which was fed a regular diet, while the remaining mice were fed a high-fat (45%) diet (HFD). The ingredient ratio of the high-fat diet is: fat 45%, carbohydrates 20%, protein 20%, vitamins and minerals 15%. Two YMP-treated groups (40 and 160 mg/kg/d; known as the YMPL and YMPH groups, respectively) of HFD mice received YMP injections. The dosage of YMP was determined by our previous pre-experiment. After four weeks, the CON mice received injections of sodium citrate buffer, while the HFD mice received intraperitoneal injections of streptozotocin (STZ, 50 mg/kg, prechilled 0.1 M/l sodium citrate buffer, pH 4.2). Three days after STZ administration, the model group (MOD) and the metformin group (160 mg/kg/d, MET) were randomly assigned. Based on earlier studies, the dosages were determined with safety in mind. Changes in body weight and blood glucose levels were also regularly recorded. After receiving therapy, the animals were killed in conformity with laws protecting animal welfare, and mouse blood was taken for the evaluation of biochemical indicators.

### 2.3 Serum biochemical analysis

Commercial assay kits (Nanjing Jiancheng Technology Co., Ltd.) were used to assess the following parameters in the serum: total protein, albumin content, globulin (GLB) content, urinary Microalbumin Creatinine Ratio (UmACR), malondialdehyde (MDA), superoxide dismutase (SOD), total antioxidant capacity (T-AOC), glutathione (GSH), interleukin-1*β* (IL-1*β*), and interleukin-6 (IL-6).

### 2.4 Histopathological analysis

For staining subsequently, the renal samples were stored in a 10% formaldehyde solution. Hematoxylin-eosin (H&E) and Masson stains were applied to the paraffin slices. The Leica DM 1 inverted microscope was used to view and take pictures of the kidney tissues.

### 2.5 Network pharmacology analysis

Following UPLC-MS/MS analysis, chemical components that were successfully identified were assessed for network pharmacology. For each of the previously described chemical components in YMP, the putative target was the analytical platform (https://prediction.charite.de/subpages/target_prediction.php). The targets were aggregated and combined using the Uniport module in Perl software, and the names of the selected targets were standardized to the official human gene to remove duplicates. Following this, we employed five databases: OMIM (https://omim.org/) (16), DrugBank (https://www.drugbank.ca/) (17), DisGeNet (https://www.disgenet.org/home/) (18), Genecards (https://www.genecards.org/) (19), and TTD (http://db.idrblab.net/ttd/) (20) to search for DN-related genes. The search results from many databases were combined to create a complete gene collection linked to DN. We eventually obtained a collection of genes connected to chemical components target genes and DN by taking the intersection of the sets of genes associated with DN and chemical components target genes. A protein-protein interaction (PPI) network with a set parameter of moderate confidence (0.400) was built using the STRING database, the target gene set for chemical components, and the gene set linked to DN (21). The PPI network was exported using STRING, and the important subnetwork was further examined using Cytoscape. Genes that had Betweenness, Closeness, and Degree values greater than the median were filtered based on CytoNca scores (22). A primary subnetwork was constructed using these filtered genes, and a final, vital subnetwork was created by further filtering the primary subnetwork. We constructed a compound-target network using the DN-related gene set and the chemical components target gene set in Cytoscape version 3.10.2. The underlying biological processes (BP), cellular components (CC), molecular functions (MF), and significant signaling pathways were then identified using enrichment analysis using Kyoto Encyclopedia of Genes and Genomes (KEGG) pathway analysis and gene ontology (GO). This investigation was conducted using the Metascape database (https://metascape.org/) (23–25).

### 2.6 Sample preparation for metabolomics analysis

The urine and serum samples were mixed three times. Following three minutes of mixing, the mixture was centrifuged for twenty minutes at 10,000 rpm and 4°C. The supernatant was collected to conduct a focused metabolomics study. A centrifuge tube was filled with 120 µl of water, 190 µl of methanol, 380 µl of dichloromethane, and 100 µl of serum. The tube was vortexed for three minutes and then centrifuged for fifteen minutes at 12,000 rpm (4°C). QC (quality assurance) sample: Ten microliters of each urine and serum sample were combined, and the mixture was centrifuged to make serum quality control samples.

### 2.7 Untargeted metabolomics analysis conditions

Using the Ultimate 3000 UHPLC technology, a metabolomics investigation with an untargeting focus was conducted. Mobile phase A was water containing 0.1% (v/v) formic acid, while mobile phase B was acetonitrile. The flow rate is 0.3 milliliters per minute. The gradient elution program used was 5% B for 0–2 minutes, 5–60% B for 2–5 minutes, 60–95% B for 5–12 minutes, 95% B for 12–20 minutes, 95–5% B for 20–20.1 minutes, and 5% B for 20.1–25 minutes. Every seven samples, a quality control sample was added to the analysis queue. The injection volume is 8 µl. The temperature of the column is 35°C. For testing, LTQ Orbitrap Velos Pro was utilized. They used an electrospray ionization (ESI) source. Positive and negative ion modes served as the foundation for this effort.

### 2.8 Metabolomics analysis

Orthogonal partial least squares discriminant analysis (OPLS-DA) and unsupervised principal component analysis (PCA) were the two main methods used for multivariate statistical studies. The load plot S-plot and variable importance in projection (VIP) plot were utilized in the OPLS-DA study to illustrate the magnitude of the metabolite contribution. The degree to which each metabolite influences the VIP value-based classification of samples from each group may be measured by this method. The t-test was used to determine whether there was a significant difference between the groups. Differential metabolites were identified using the following criteria: VIP >1, t-test (*p* < 0.05), and fold change (FC) values (FC > 1.5 or < 0.7).

### 2.9 Transcription analysis

Thorough preprocessing was performed on the raw sequencing data to provide clean reads that could be used for additional research. Initially, reads comprising more than 1% of unknown bases, reads polluted by adaptor sequences, and reads with a low-quality base ratio (quality score ≤ 15) exceeding 40% were filtered out using SOAPnuke (version 1.6.5). Following that, the clean reads were saved in the FASTQ format. Bowtie2 (version 2.4.5) was then used to map the cleaned data to the constructed unique gene sequences. After mapping, RSEM (version 1.3.1) was used to precisely quantify the gene expression levels. Public databases like Gene Ontology (GO) and the Kyoto Encyclopedia of Genes and Genomes (KEGG) were used to annotate the genes. Important details regarding the biological roles of the genes were revealed by this annotation process. In order to remove differentially expressed genes (DEGs) with fragments per kilobase of transcript per million fragments mapped (FPKM) values less than 1 in both comparison groups and to make sure that the detection rate of DEGs is greater than or equal to 66.6% in at least one group, DESeq2 was used to identify genes that are differentially expressed between groups with fold change (FC) > 2 or < 0.5. The DEGs were functionally categorized using the KEGG and GO annotations. KEGG enrichment analysis was performed using R’s phyper function, and GO enrichment analysis was carried out using the TermFinder package to obtain more understanding of the enriched biological processes and pathways. Significantly enriched genes and pathways were identified using an AQ value threshold of < 0.05.

### 2.10 Statistical analysis

The program SPSS 22.0 was used to evaluate the data. The results were displayed using the mean plus or minus standard deviation. One-way ANOVA and the Tukey post-test were used to create group comparisons; a *p*-value of less than 0.05 was considered statistically significant.

## 3 Results

### 3.1 Compound identification by UPLC-MS/MS

The primary compounds in YMP were discovered using UPLC-MS/MS. The MS/MS fragment pattern served as the primary technique for compound identification in the evidence. By cross-referencing with database data and published literature, 43 compounds in YMP were found based on similarities in mass spectrometric behavior and retention time (**Supplementary Figure S1**). Among the several kinds of compounds discovered are lignins and flavonoids, among others. A comprehensive list of these chemicals can be found in **Table 1**.

**Table 1:** Chemical compounds identified in YMP by UPLC-MS/MS.

No.	Rt (min)	Molecular formula	Compound identified	CAS	Theoretical mass (m/z)	Accurated mass (*m/z)*	Error (ppm)	Fragmentions
1	3.81	C_19_H_18_O_8_	Casticin	479-91-4	374.1007	374.1002	1.36	342.0736, 360.0840
2	3.82	C_12_H_22_O_11_	Sucrose	57-50-1	342.1165	342.1162	0.72	71.0144, 89.0249, 113.0556
3	4.10	C_16_H_18_O_9_	Scopolin	531-44-2	354.095	354.0951	-0.15	133.0334, 178.0265, 193.0523
4	4.76	C_8_H_8_O_2_	4’-Hydroxyacetophenone	99-93-4	136.0525	136.0524	0.27	93.0346, 108.0217, 135.0451
5	6.09	C_15_H_16_O_9_	Esculin	531-75-9	340.0795	340.0794	0.13	105.0353, 133.0296, 177.0201
6	7.50	C_25_H_24_O_12_	Cynarine	212891-05-9	516.1267	516.1268	-0.21	145.0260, 163.0383
7	7.70	C_25_H_24_O_12_	Isochlorogenic acid B	89886-31-7	516.1279	516.1268	2.2	163.0419, 319.0909, 499.1293
8	7.81	C_25_H_24_O_12_	Isochlorogenic acid C	14534-61-3	516.127	516.1268	0.36	173.0402, 353.0853, 515.1177
9	7.90	C_16_H_18_O_10_	Fraxin	524-30-1	370.0904	370.09	0.97	91.0554, 185.0437, 231.0339
10	8.11	C_12_H_16_O_4_	Methyl 3-(3,4-dimethoxyphenyl)propanoate	27798-73-8	224.1049	224.1049	0.34	151.0754, 163.0383
11	8.14	C_15_H_22_O_8_	Homovanillyl alcohol 4-O-glucoside	104380-15-6	330.1314	330.1315	-0.09	185.0452, 276.1588, 276.1588
12	8.18	C_10_H_10_O_4_	Ferulic acid	1135-24-6	194.0579	194.0579	-0.08	114.1446, 133.0289, 160.8840
13	8.68	C_27_H_30_O_15_	Nicotiflorin	17650-84-9	594.1585	594.1585	0.07	287.0551, 449.1078
14	8.99	C_10_H_8_O_4_	Scopoletin	92-61-5	192.0422	192.0423	-0.13	94.0405, 122.0359, 133.0271
15	9.97	C_22_H_22_O_9_	Ononin	486-62-4	430.1267	430.1264	0.76	267.0661, 475.1242
16	11.21	C_27_H_30_O_16_	Osyritin	1340-08-5	610.1459	610.1532	-0.38	255.0270, 271.0232, 300.0243
17	11.26	C_26_H_28_O_14_	Neoshaftoside	51938-32-0	564.1476	564.1479	-0.51	117.0336, 353.0854
18	11.27	C_27_H_30_O_16_	Rutin	153-18-4	610.1462	610.1532	0.25	607.1321, 609.1472
19	11.29	C_26_H_28_O_14_	Apiin	26544-34-3	564.1481	564.1479	0.27	91.0549, 269.0454
20	11.30	C_27_H_30_O_16_	Lilyn	31512-06-8	610.1534	610.1532	-0.07	227.0343, 255.0294, 284.0323
21	11.35	C_12_H_14_O_4_	Ethyl ferulate	4046-02-0	222.089	222.0892	-0.89	91.0549, 177.0202
22	11.36	C_16_H_12_O_7_	Isorhamnetin	480-19-3	316.058	316.0583	-1.04	153.0168, 229.0478, 302.0418
23	11.59	C_11_H_10_O_4_	Scoparone	120-08-1	206.058	206.0579	0.38	151.0754, 179.0694
24	12.06	C_21_H_18_O_12_	Luteolin 7-glucuronide	29741-10-4	462.0795	462.0798	-2.94	153.0160, 287.0550
25	12.48	C_27_H_30_O_15_	Saponarin	20310-89-8	594.1581	594.1585	-0.58	353.0853, 449.1077
26	12.69	C_28_H_32_O_16_	Keioside	107740-46-5	624.17	624.169	0.71	71.0504, 274.0420
27	12.71	C_28_H_32_O_16_	Narcissin	604-80-8	624.1694	624.169	0.6	285.0436, 302.0450, 317.0656
28	14.54	C_15_H_10_O_7_	Quercetin	117-39-5	302.0423	302.0427	-1.1	165.0183, 247.0596, 257.0440
29	14.54	C_20_H_18_O_6_	Isolicoflavonol	94805-83-1	354.1036	354.1103	0.01	146.0599, 160.0749, 189.1023
30	15.13	C_10_H_10_O_2_	Methyl cinnamate	103-26-4	162.0678	162.0681	-1.73	131.0491, 160.0749
31	18.42	C_15_H_14_O_6_	Cianidanol	7295-85-4	290.0789	290.079	-0.62	123.0436, 165.0542, 291.0862
32	18.66	C_14_H_18_O_2_	Isopentyl cinnamate	7779-65-9	218.1309	218.1307	0.95	131.0492, 182.0422
33	20.96	C_15_H_10_O_5_	Apigenin	8002-66-2	270.0525	270.0528	-1.06	66.0043, 117.0336, 182.0421
34	26.78	C_20_H_18_O_5_	Demethoxycurcumin	22608-11-3	338.1152	338.1154	-0.69	119.0445, 147.0427
35	36.39	C_17_H_14_O_6_	Cirsimaritin	6601-62-3	314.0792	314.079	0.51	108.0212, 136.0159, 254.0577
36	36.74	C_14_H_28_O	2-Tetradecanone	2345-27-9	212.2139	212.214	-0.76	71.0503, 165.0542
37	36.78	C_17_H_14_O_6_	Gnaphaliin	33803-42-8	314.0794	314.079	1.12	282.9710, 297.9860, 313.0150
38	37.57	C_19_H_20_O_5_	Clausenidin	28384-44-3	328.1308	328.1311	-0.73	287.0550, 291.0861
39	37.97	C_19_H_20_O_5_	2’,4,4’,6’-Tetramethoxychalcone	25163-67-1	328.1384	328.1311	0.29	165.0542, 287.0551
40	41.63	C_19_H_20_O_5_	3,4,2’,5’-Tetramethoxychalcone	1383425-70-4	328.1308	328.1311	-0.77	165.0182, 314.0791
41	53.52	C_16_H_32_O_2_	Butyl laurate	106-18-3	256.24	256.2402	-0.75	177.0202, 182.0422
42	53.91	C_16_H_32_O_2_	Palmitic Acid	1957/10/3	256.2401	256.2402	-0.6	203.2024, 255.2330
43	54.08	C_16_H_32_O_2_	Ethyl tetradecanoate	124-06-1	256.2401	256.2402	-0.36	69.0331, 118.0242, 168.0421

### 3.2 Network pharmacological analysis

**Figure 1:**
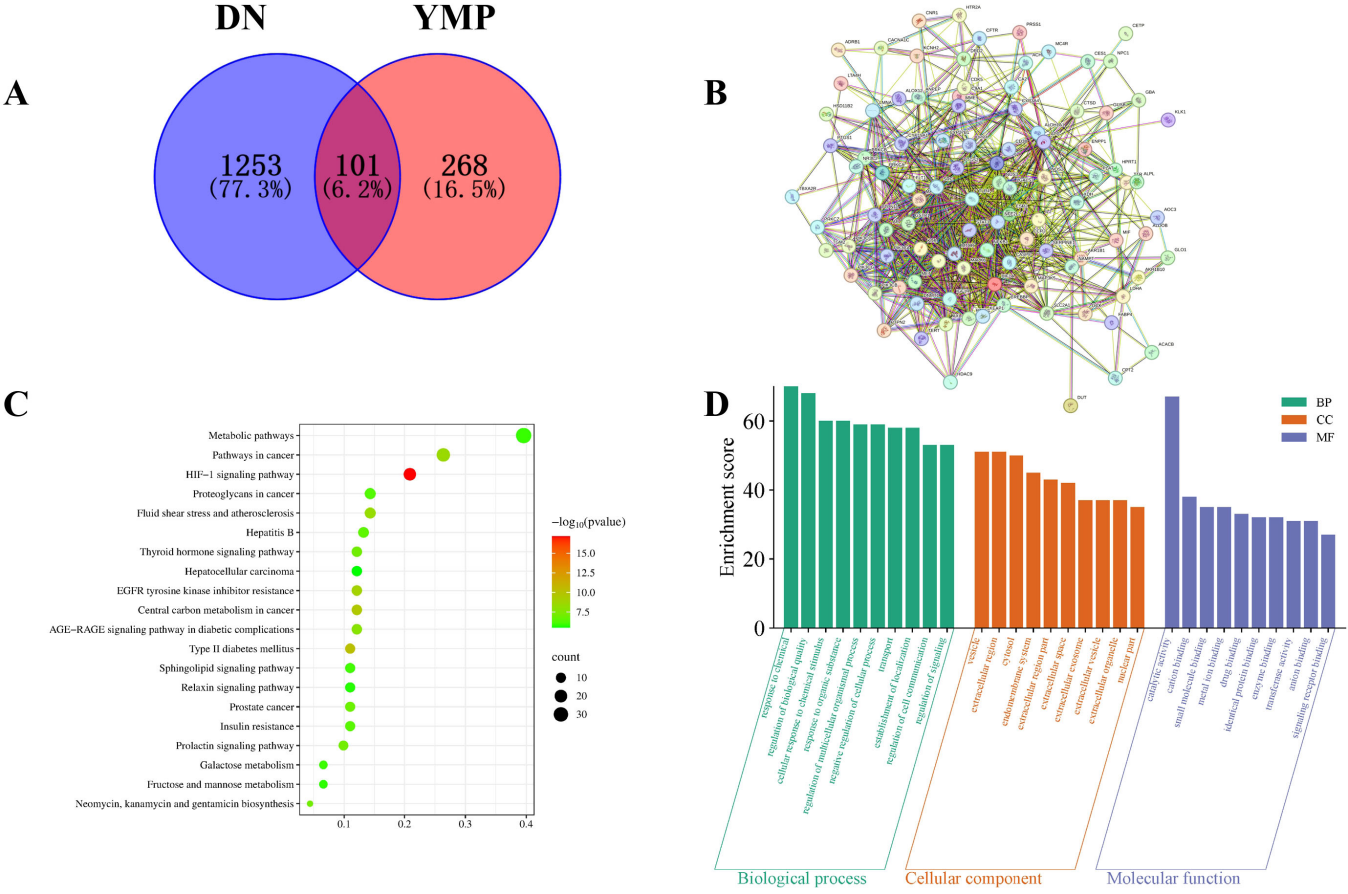
Venn diagram **(A)**, PPI network **(B)**, KEGG enrichment analysis **(C)**, GO enrichment analysis **(D)**.

Integrated multi-database analysis identified 101 high-confidence targets linking YMP components to DN pathogenesis. Initial screening revealed 369 YMP-associated targets (Super-pred) and 1,354 unique DN-related genes (aggregated from OMIM, DrugBank, GeneCards, PharmGKB, and TTD). Crucially, the intersection of these datasets yielded 101 shared targets (**Figure 1A**), suggesting convergent mechanisms for therapeutic intervention.

Protein-protein interaction analysis (STRING; **Figure 1B**) revealed significant modularity among these targets, indicating functional cooperativity in DN-relevant processes. KEGG pathway enrichment identified 152 significantly enriched pathways, with top pathways implicating HIF-1 signaling, metabolic dysregulation, and AGE-RAGE signaling - all established drivers of DN progression (**Figure 1C**). Notably, enriched cancer pathways were deprioritized as likely context-independent artifacts.

GO enrichment analysis further refined functional insights: Targets were significantly associated with cation/small molecule binding (MF), extracellular vesicle regulation (CC), and cellular response to chemical stimuli (BP) (**Figure 1D**). These terms align mechanistically with DN pathophysiology, particularly extracellular matrix remodeling, exosomal communication, and hyperglycemia-induced cellular stress.

### 3.3 Glycemia regulation analysis

**Figure 2:**
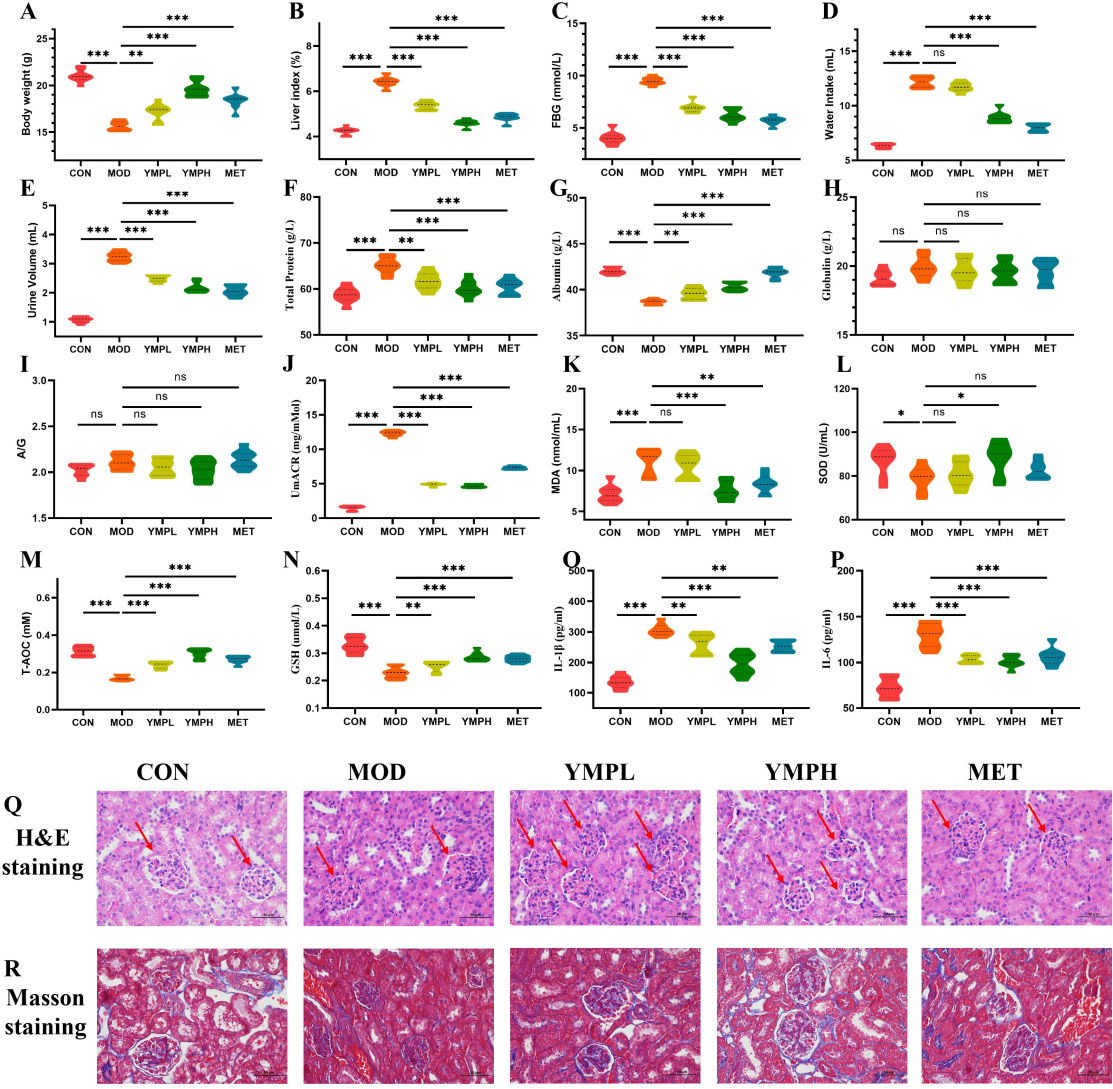
Body weight gain **(A)**, liver index **(B)**, FBG **(C)**, Water intake **(D)**, Urine volume **(E)**, Total protein **(F)**, Albumin **(G)**, GLB **(H)**, A/G **(I)**, UmACR **(J)**, MDA **(K)**, SOD **(L)**, T-AOC **(M)**, GSH **(N)**, IL-1β **(O)**, IL-6 **(P)**, H&E staining **(Q)**, Masson staining **(R)** (×200). ^*^ represents for vs MOD group (p < 0.05), ^**^ represents for vs MOD group (p < 0.01), ^***^ represents for vs MOD group (p < 0.01), respectively. (n=8) ns represents for vs MOD group (p ≥ 0.05).

CON mice displayed normal agility and physiological parameters. STZ/HFD-induced diabetic MOD mice exhibited characteristic metabolic dysfunction, including significant weight loss (p < 0.001), lethargy, polyuria, and reduced body mass, confirming successful model establishment (**Figure 2A**). Notably, the liver index of the diabetic mice was significantly greater (*p* < 0.001) than that of the CON group, suggesting that the organ index could be used to assess a drug’s toxicity. YMP appears to have a protective effect against STZ damage, as evidenced by the statistically significant difference (*p* < 0.001) between YMP and the other groups in **Figure 2B**. YMP at 40 and 160 mg/kg may considerably lower the FBG (*p* < 0.001) in comparison to the MOD group (**Figure 2C**). The MOD and CON groups drank significantly different amounts of water (*p* < 0.001). The group that got a high dosage of YMP had lower water administration (*p* < 0.001), while the YMPL group did not differ from the administration groups in terms of water intake following administration (*p* > 0.05) (**Figure 2D**). YMP administration demonstrated significant therapeutic effects.

### 3.4 Renal protection analysis

Diabetic (MOD) mice exhibited significantly elevated urine volume compared to controls (CON) (p < 0.001). YMP treatment displayed a downward trend and the impact was rose with concentration, with both YMPL and YMPH groups showing significantly lower urine output versus MOD (p < 0.001; **Figure 2E**). Urinary total protein excretion was markedly increased in MOD mice (p < 0.001). The treatment group showed a reduction in proteinuria with increasing concentrations (**Figure 2F**). Serum albumin levels were substantially reduced in MOD mice (p < 0.001), while YMP administration significantly restored albumin concentrations (p < 0.01; **Figure 2G**). No significant differences were observed in serum globulin (GLB) levels between MOD and CON groups (p > 0.05). YMP treatment did not alter GLB concentrations (**Figure 2H**). Similarly, the albumin/globulin ratio (A/G) showed no statistically significant changes across groups (**Figure 2I**). MOD mice displayed significantly elevated urinary microalbumin/creatinine ratio (UmACR) versus CON (p < 0.001). Both YMP doses effectively reduced UmACR levels compared to MOD (p < 0.001; **Figure 2J**). YMP significantly ameliorates diabetic nephropathy by reducing polyuria, proteinuria, albuminuria, and restoring serum albumin levels.

### 3.5 Oxidative stress and inflammatory response

To determine the effect of YMP on the inflammatory response and oxidative stress of the STZ-induced diabetic mice, proinflammatory cytokines and oxidative stress markers were evaluated. Important indicators of the body’s antioxidant status include the levels of MDA, SOD, T-AOC, and GSH. In contrast to the CON group, the MOD group had significantly lower levels of GSH, SOD, and T-AOC and higher levels of MDA. The MDA of the MET (*p* < 0.01) and YMPH (*p* < 0.001) groups was significantly lower than that of the MOD group. However, **Figure 2K** shows that YMPL did not significantly down-regulate MDA (*p* > 0.05). The MET and YMPL groups did not exhibit an increase in SOD when compared to the MOD group; however, there was evidence that the group administered a high dosage of YMP would change the SOD concentration (*p* < 0.05) (**Figure 2L**). The MOD group had a considerably greater YMPH than the CON group (*p* < 0.001). T-AOC decreased in each delivery group, and the impact was rose with concentration (*p* < 0.001) (**Figure 2M**). Notably, the GSH levels of the diabetic mice were significantly lower (*p* < 0.001) than those of the CON group, suggesting that GSH could be a helpful marker (**Figure 2N**). Compared to the CON group, the MOD group’s levels of IL-1*β* and IL-6 were considerably greater (*p* < 0.001). IL-1*β* levels were considerably lower in the YMPL and YMPH groups than in the MOD group (*p* < 0.001), and the impact was rose with concentration (**Figure 2O**). The IL-6 levels of the YMPL and YMPH groups did not exhibit a concentration-dependent effect, and they were significantly lower than those of the MOD group (*p* < 0.001) (**Figure 2P**). Based on the comprehensive findings, YMP ameliorates oxidative stress and inflammation in diabetic mice.

### 3.6 Renal pathological analysis

CON animals did not exhibit hyperplastic mesangial matrix, thickening of the capillary basement membrane, hypertrophy of the glomerulus as shown by H&E staining. The glomerulus exhibited mild hypertrophy and the mesangial area significantly increased in the MOD group. The glomerular hypertrophy in the YMPH group was notably less pronounced than in the other groups. The relevant data was shown in **Figure 2Q**. The collagen fibers appeared blue after Masson staining. Using a semiquantitative method, it was rated based on the degree of collagen fiber proliferation. The CON group’s tubular and glomerular structures were normal. The MOD group’s glomerular collagen hyperplasia was much higher than that of the CON group. The findings demonstrated that YMP was helpful in treating DN, with a higher intervention impact at high doses (**Figure 2R**).

### 3.7 Untargeted metabolomics analysis

**Figure 3:**
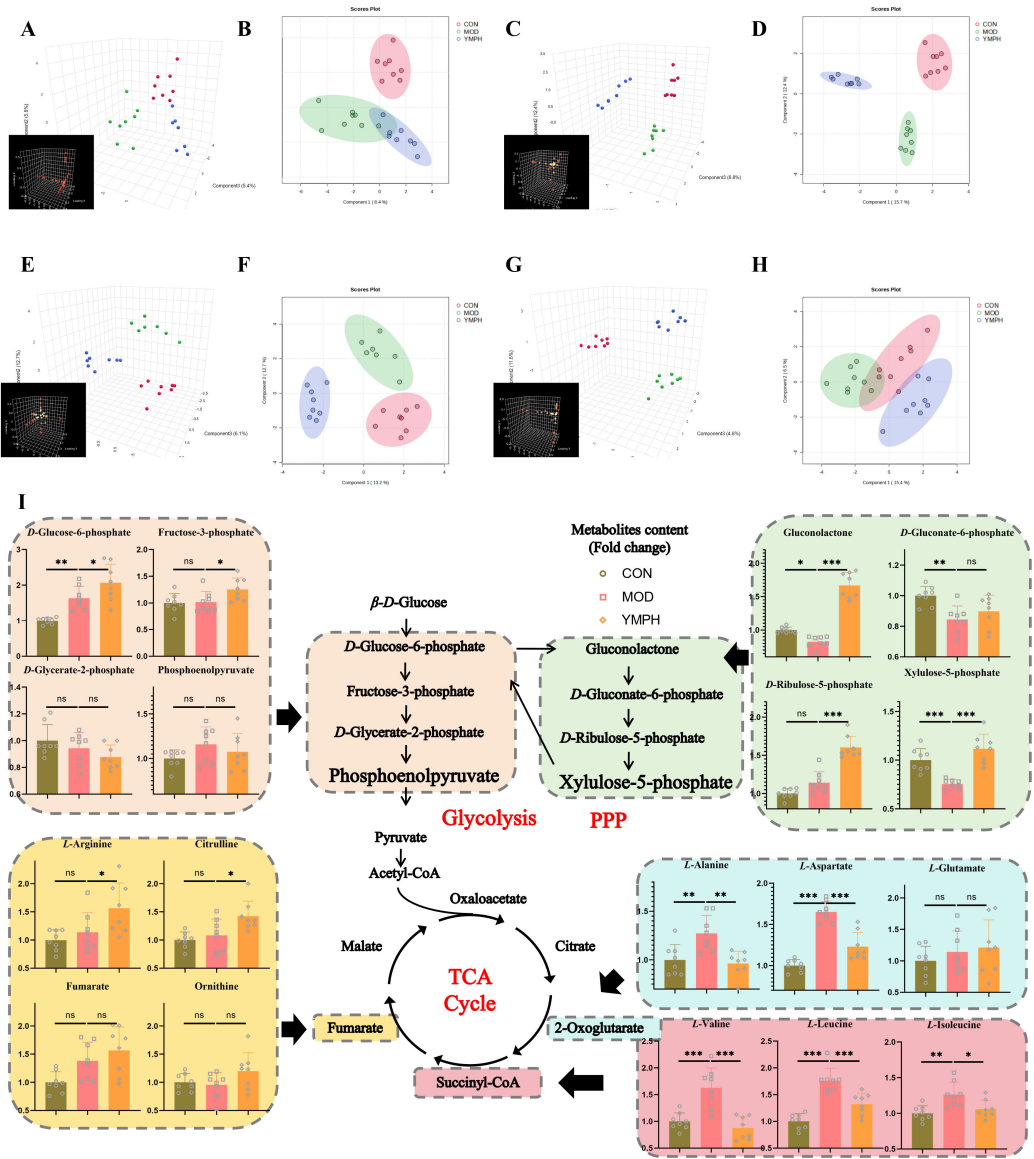
PCA 3D plots **(A)**, PCA 2D plots **(B)**, in positive serum mode, PCA 3D plots **(C)**, PCA 2D plots **(D)**, in negative serum mode, PCA 3D plots **(E)**, PCA 2D plots **(F)**, in positive urine mode, PCA 3D plots **(G)**, PCA 2D plots **(H)**, in negative urine mode, the change of key ingredient content according to metabolomics **(I)**. ^*^ represents for vs MOD group (p < 0.05), ^**^ represents for vs MOD group (p < 0.01), ^***^ represents for vs MOD group (p < 0.01), respectively. (n=8) ns represents for vs MOD group (p ≥ 0.05).

Serum and urine samples were subjected to non-targeted metabolomic analysis in positive and negative modes. Each sample was clearly separated from the mice’s CON group. The results demonstrated that the metabolisms of the MOD and administration groups were abnormal. There were 256 and 273 metabolites in positive and negative modes, respectively, in the serum samples (**Supplementary Tables S1**, **S2**). In urine samples, 427 and 460 metabolites were found in positive and negative modes, respectively (**Supplementary Tables S3**, **S4**). Based on the global features of the raw data, OPLS-DA analysis was carried out to distinguish between the groups and show the metabolic differences. The various metabolite groups were obviously distinct from one another based on differences in physiological markers. OPLS-DA may eliminate negligible discrepancies and increase classification accuracy. The two groups were separated using the OPLS-DA model score plots (**Figures 3A-H**). In MOD groups, endogenous chemical metabolisms were changed. Metabolic profiles could be used to identify biomarkers. **Supplementary Figures S2A-H** displayed the R2Y and Q2 values for serum and urine samples in both positive and negative modes. The MOD groups’ and the CON mice’s OPLS-DA score graphs were clearly different from one another. The metabolic profiles of the serum and urine also showed discernible changes after YMP administration. It was shown that tissue samples were more effective in detecting metabolic fingerprints specific to individual organs than biofluids that depict the metabolic state of the complete body. Serum samples from the CON and MOD groups showed differences in the pathways of glycolysis/gluconeogenesis, aspartate, glutamate, and neomycin biosynthesis, gentamicin, kanamycin, and nitrogen metabolism, pentose and glucuronate interconversions, starch and sucrose metabolism, and selenium metabolism. Numerous metabolic pathways, such as the biosynthesis of valine, leucine, and isoleucine, the metabolism of arginine and proline, the metabolism of nitrogen, the metabolism of butanoates, and the metabolism of histidine, were disrupted in urine samples. The biosynthesis of arginine was also disturbed. The following pathways were affected in serum samples between the MOD and YMPH groups: pentose phosphate pathway, glycolysis/gluconeogenesis, alanine, aspartate, and glutamate metabolism, glyoxylate and dicarboxylate metabolism, arginine and proline metabolism, nitrogen metabolism, arginine biosynthesis, butanoate metabolism, histidine metabolism, pentose and glucuronate interconversions, and citrate cycle (TCA cycle). Urine samples showed disruptions to multiple metabolic pathways: Glycolysis/Gluconeogenesis; Alanine, aspartate, and glutamate metabolism; Pantothenate and CoA biosynthesis; Valine, leucine, and isoleucine biosynthesis; Arginine biosynthesis; *D*-Amino acid metabolism; Nicotinate and nicotinamide metabolism; Histidine metabolism. Urine positive modes showed complementary but less comprehensive pathway alterations (**Figure 3I**). YMP administration significantly reversed diabetes-associated metabolic disruptions, with YMPH showing pronounced efficacy.

### 3.8 Transcription analysis

**Figure 4:**
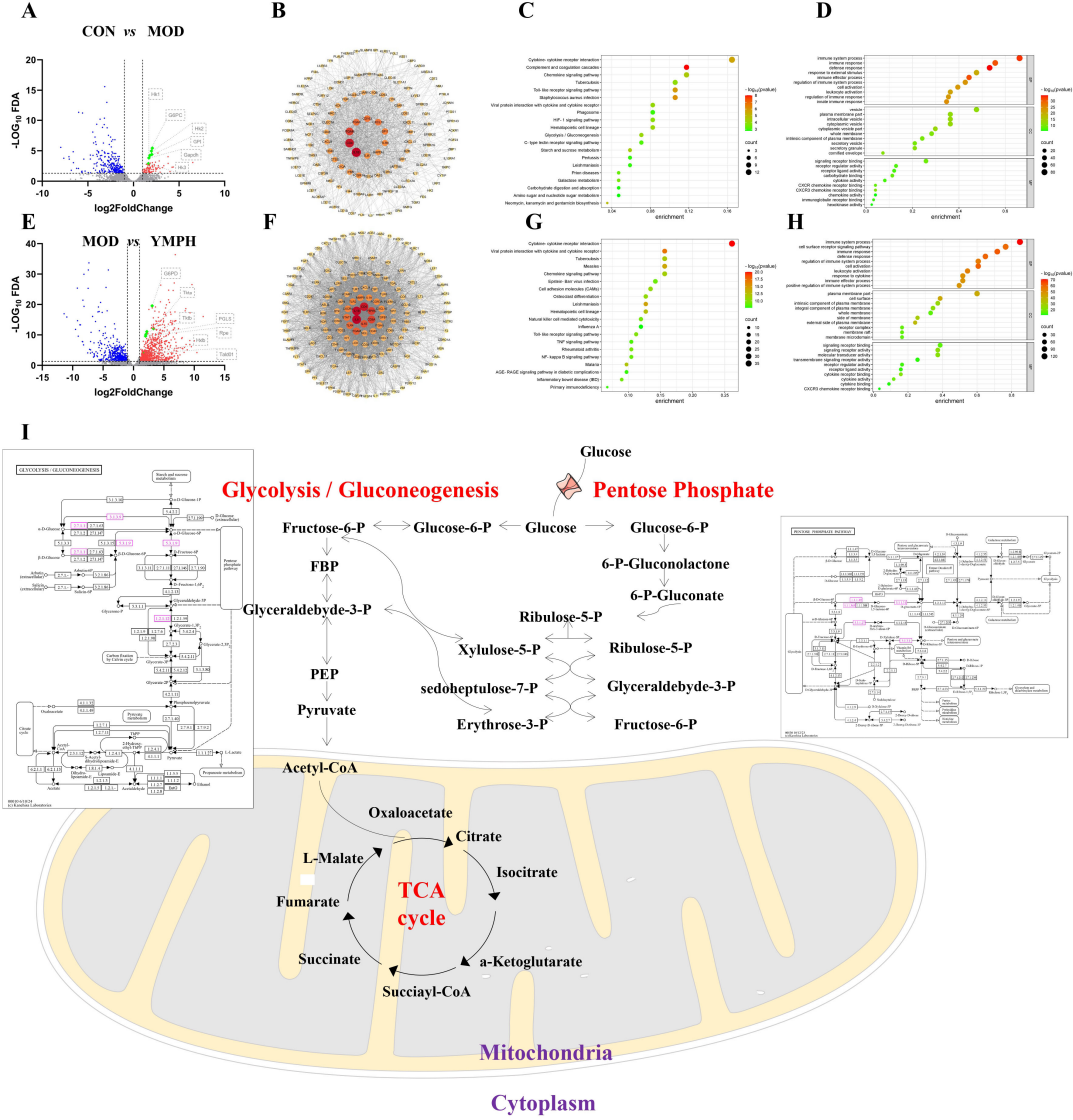
Volcano map between CON and MOD **(A)**, the key differential genes arranged by values of degree between CON and MOD **(B)**, the KEGG enrichment analysis of key differential genes between CON and MOD **(C)**, GO enrichment analysis of key differential genes between CON and MOD **(D)**, Volcano map between MOD and YMPH **(E)**, the key differential genes arranged by values of degree between MOD and YMPH **(F)**, the KEGG enrichment analysis of key differential genes between MOD and YMPH **(G)**, GO enrichment analysis of key differential genes between MOD and YMPH **(H)**, the change of key ingredient genes according to transcriptome **(I)**.

There were 87 and 323 genes in up and down modes, respectively, between the CON and MOD groups (**Figure 4A**, **Supplementary Tables S5**, **S6**). Significantly increased were the genes GPI, GAPDH, G6PC, HK2, HK1, and HK3 linked to Glycolysis/Gluconeogenesis. The intricate relationships that these target genes’ proteins demonstrated are depicted in the protein-protein interaction network that was generated from the STRING database (**Figure 4B**). A KEGG enrichment analysis was performed to determine which pathways were impacted by the 380 target genes. 36 KEGG pathways were found to be significantly enriched (**Supplementary Table S7**). These results suggest that the target genes are linked to several networks, including Complement and coagulation cascades, Staphylococcus aureus infection, Toll-like receptor signaling pathway, Cytokine-cytokine receptor interaction, Neomycin, kanamycin and gentamicin biosynthesis, Chemokine signaling pathway, Starch and sucrose metabolism, Hematopoietic cell lineage, Glycolysis/Gluconeogenesis, Viral protein interaction with cytokine and cytokine receptor. **Figure 4C** showed the bubble plot with the top 10 KEGG pathways. The 380 target genes’ MF, CC, and BP were identified using GO enrichment analysis. 1111 highly enriched GO keywords were discovered. **Figure 4D** and **Supplementary Table S8** displayed a graphic representation of the top 10 terms. According to the analysis’s findings, these target genes are essential for biological functions like defense response, immune system process, immune effector process, cytoplasmic vesicle part, cytoplasmic vesicle, intracellular vesicle, CXCR3 chemokine receptor binding, signaling receptor binding, carbohydrate binding.

There were 958 and 605 genes in up and down modes, respectively, between the MOD and YMPH groups (**Figure 4E**, **Supplementary Tables S9**, **S10**). Significantly increased were the genes G6PD, PGLS, RPE, TALDO1, HXLB linked to Pentose phosphate pathway. The intricate relationships that these target genes’ proteins demonstrated are depicted in the protein-protein interaction network that was generated from the STRING database (**Figure 4F**). A KEGG enrichment analysis was performed to determine which pathways were impacted by the 1563 target genes. 88 KEGG pathways were found to be significantly enriched (**Supplementary Table S11**). These results suggest that the target genes are linked to several networks, including Cytokine-cytokine receptor interaction, Viral protein interaction with cytokine and cytokine receptor, Leishmaniasis, Measles, Hematopoietic cell lineage, Malaria, Tuberculosis, Chemokine signaling pathway, Osteoclast differentiation, Cell adhesion molecules (CAMs). **Figure 4G** showed the bubble plot with the top 10 KEGG pathways. The 1563 target genes’ MF, CC, and BP were identified using GO enrichment analysis. 2861 highly enriched GO keywords were discovered. **Figure 4H** and **Supplementary Table S12** displayed a graphic representation of the top 10 terms. According to the analysis’s findings, these target genes are essential for biological functions like immune system process, immune response, cell activation, cell surface, plasma membrane part, external side of plasma membrane, signaling receptor binding, signaling receptor activity, molecular transducer activity. When administering STZ, the model groups activate glycolysis and gluconeogenesis, which is the same outcome of transcription analysis as it is of untargeted metabolomics study. But when YMPH was given, the pentose phosphate pathway was triggered (**Figure 4I**).

### 3.9 Interaction among compound, gene and metabolites analysis

**Figure 5:**
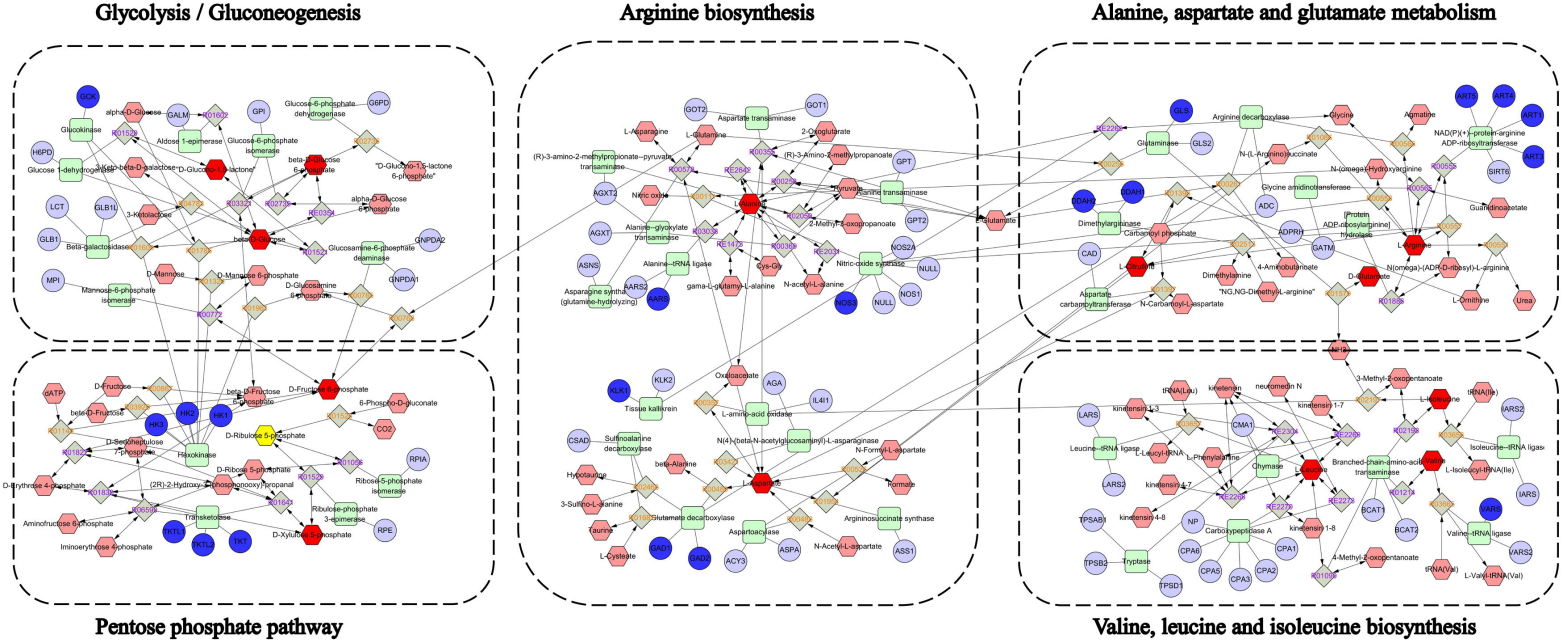
Overview of interactions among compounds, genes.

To elucidate YMP’s therapeutic mechanism, an integrated multi-omics network was constructed by combining key targets from network pharmacology with significantly altered metabolites from metabolomics (**Figure 5**). This synthesis revealed critical interactions between: Gene targets: Arginine metabolism regulators (ART1/3/4/5, DDAH1/2, NOS3), glucose phosphorylation enzymes (GCK, HK1/2/3), and neurotransmitter modulators (GAD1/2). Differential metabolites: Glycolytic intermediates (*β*-D-glucose, glucose-6-phosphate, fructose-6-phosphate), pentose phosphate pathway components (gluconolactone, ribulose-5-phosphate), and amino acids (alanine, aspartate, glutamate, citrulline, arginine, branched-chain amino acids). Pathway enrichment analysis identified five core disrupted pathways: glycolysis/gluconeogenesis, pentose phosphate pathway, arginine biosynthesis, alanine, aspartate and glutamate metabolism; valine, leucine and isoleucine biosynthesis. These findings suggest YMP restores metabolic homeostasis through coordinated regulation of central carbon metabolism and amino acid biosynthesis.

## 4 Discussion

One of the most important microvascular effects of diabetes is DN, which markedly increased cardiovascular morbidity and death. The numerous signaling pathways and targets implicated in the pathogenesis of DN were yet unknown (26). Despite having different effects, these signaling pathways and targets were closely related and mishybrid. TCM has been shown to provide a significant advantage in the treatment of DN. By focusing on several targets and pathways, it not only reduces clinical symptoms but also delays the course of the disease. The majority of the time, diseases developed and progressed as dynamic processes (27).

The peel of *Zea mays* L., which was brought to China during the Ming Dynasty, makes up corn husk. It is thought to be a diuretic that can treat edema, urinary stones, unpleasant urination, and appetite loss. Network pharmacology was a systematic research methodology that used clinical and experimental data to speed up drug discovery and development (28). According to KEGG enrichment analysis, the target genes are linked to several networks, including those related to cancer pathways, metabolic pathways, and the HIF-1 signaling pathway in problems related to diabetic nephropathy. In HFD/STZ-induced DN mice, Dang-Gui-Bu-Xue decoction (DBD) controlled energy and water intakes, impairments of glucose and lipid metabolism, renal dysfunction, glomerular filtration rate, renal interstitial glycogen buildup, and renal fibrosis (29). The AGEs/RAGE pathway was the most prevalent pathway for Huang-Lian-Jie-Du Decoction (HLJDD) against DN, according to *in silico* network pharmacology. Through the improvement of renal damage and glycolipid metabolic abnormalities, HLJDD demonstrated protective benefits against DN *in vivo*. Additionally, we confirmed for the first time that HLJDD protected against DN via controlling the AGEs/RAGE/Akt/Nrf2 pathway (30).

YMP’s active ingredients, including as scopolin, esculin, ferulic acid, and isorhamnetin, have a long history of use in the treatment of diabetes and related conditions. Scopolin and neochlorogenic were shown to be the most significant chemicals in *B. vulgaris* that controlled PGC-1α, AMPK, and GSK3 based on the results of molecular docking (31). As evidenced by Naseem’s suppression of human insulin fibrillation, esculin’s anti-amyloidogenic capabilities may make it a promising therapy option for neurodegenerative diseases in the near future (32). The mechanisms behind fraxin’s renal protective properties against diabetic renal tubulointerstitial fibrosis (RIF) were investigated and explained by Zeng. In NRK-52E cells exposed to high glucose (HG), Fraxin improved cellular shape, inhibited abnormal expression of markers associated with the epithelial-to-mesenchymal transition (EMT) and proinflammatory cytokines, and subsequently reduced the creation of extracellular matrix (ECM). Fraxin reduced extracellular matrix deposition in the renal tubule interstitium, inhibited abnormal production of proinflammatory cytokines and EMT-related markers, and markedly enhanced renal function *in vivo* in *db/db* rats (33). Ferulic acid significantly (*p* < 0.05) decreased the activities of *α*-glucosidase, α-amylase, and pancreatic lipase while increasing glucose absorption in isolated rat psoas muscles. Glutathione (GSH), superoxide dismutase (SOD), and catalase activities were considerably (*p* < 0.05) decreased when oxidative pancreatic injury was caused, while nitric oxide (NO), malondialdehyde (MDA), acetylcholinesterase, and chymotrypsin activities were increased. Mao demonstrated that isorhamnetin enhanced erectile function, reduced collagen content, and increased smooth muscle content in the corpus cavernosum (CC) of diabetic rats. Additionally, isorhamnetin enhanced the activities of SOD, GPx, and CAT in addition to the levels of NO, lowered the levels of MDA in corpus cavernosum tissues, and elevated the levels of the anti-inflammatory mediators IL-10 and IL-4. Additionally, it raised the levels of TNF-α, IL-1β, and IL-6, which are pro-inflammatory proteins. Furthermore, in the CC tissues of diabetic rats, isorhamnetin increased the quantity of CD31, reduced apoptosis, and promoted the PI3K/AKT/eNOS signaling pathway (34). Li found that apigenin accelerated the conversion of M1-type macrophages into M2 type macrophages, inhibited the production of cellular M1-type factors TNF-*α* and IL-1*β*, and increased the secretion of M2-type factors IL-10 and TGF-*β* in LPS-stimulated RAW264.7 cells. It also elevated the expression of miR-21. Furthermore, we established an *in vitro* co-culture system between HUVECs and macrophages and found that apigenin increased the expression of miR-21 in macrophages, which in turn improved the motility, proliferation, and VEGF production of HUVECs (35). Cirsimaritin effectively reduced the elevated serum glucose levels in diabetic rats as compared to the vehicle control group (*p* < 0.001). Simitin reversed the increased serum insulin levels in the treated diabetic group of rats compared to the vehicle control group (*p* < 0.01). The homeostasis model assessment of insulin resistance (HOMA-IR) decreased in the diabetic rats treated with cirsimaritin compared to the vehicle controls. GLUT4 (*p* < 0.01) and pAMPK-α1 (*p* < 0.05) protein levels were upregulated in both skeletal muscle and adipose tissue following cirsimaritin administration. Cercaritin may enhance the expression of GLUT2 and AMPK proteins in the liver (*p* < 0.01, p < 0.05, respectively). Cirsimaritin-treated diabetic rats exhibited reduced LDL-C, triglyceride, and cholesterol levels in comparison to the vehicle controls (*p* < 0.001) (36).

The metabolomics approach was a powerful technique that could reveal minute details of biological pathways or mutations. Using network pharmacology and metabolomics, we found that amino acid metabolism was the pathway most significantly affected. Energy metabolism-related pathways were also affected. Targeted metabolomics confirmed changes in downstream small-molecule metabolites from pathways that were the focus of untargeted metabolomics. Microarray and real-time PCR studies have shown that ferulic acid (FA) boosts the expression of numerous genes related to energy metabolism, dopamine synthesis, and cell survival and proliferation in the limbic region of the mouse brain. Interestingly, compared to bupropion, an antidepressant drug, FA dramatically boosts energy metabolism. Additionally, FA decreases glycogen levels in the limbic system of mice’s brains while increasing catecholamines (dopamine and noradrenaline), ATP, and brain-derived neurotrophic factor (37). It was discovered that the dilated tubules, the region of interstitial fibrosis, and renal glycolysis were all improved by proximal tubule-specific overexpression of the platelet isoform of phosphofructokinase 1 (PFKP). On the other hand, it was discovered that PFKP knockdown suppressed these processes. Furthermore, PFKP overexpression increased TGF-*β*1-induced glycolysis in the human proximal tubular epithelial cells (PTECs) line, while PFKP knockdown decreased it. Mechanistic insight into how TGF-*β*1 opposes the SMAD family member 3-SP1 complex by recruiting small molecules to the PFKP promoter to boost its expression was obtained by chip-qPCR study. PTEC-induced elevated glycolysis and renal fibrosis was improved in mice treated with isorhamnetin (38). Sánchez-Alegría found that neurons derived from human neuroblastoma MSN generated ATP through mitochondrial metabolism when exposed to high but safe quantities of palmitic acid. This was connected to a decrease in insulin signaling in the neurons and an increase in cytosolic Ca^2+^. These findings reveal a new method by which saturated fats lead to Ca^2+^ entry and insulin resistance, which could be the underlying reason of the elevated brain vulnerability associated with metabolic diseases (39).

Amino acids are essential for vital physiological functions like glycolysis and the tricarboxylic acid cycle. All facets of the organism’s cellular metabolism, including the synthesis of proteins, lipids, and nucleic acids, depended on amino acid metabolism. Amino acid metabolism is one of the most significant cellular metabolism processes in living organisms. The metabolism of amino acids had an important effect on immune cell activity. Scopolettin enrichment may help LG31 plants survive atrazine stress, according to earlier studies that found that Gongai2 (GA2) plants had higher atrazine tolerance because of enhanced proline biosynthesis and glutathione metabolism (40). Arginase catalyzes the conversion of *L*-arginine to *L*-ornithine and urea in the last stage of the urea cycle, removing toxic ammonia. *L*-ornithine is further broken down by ornithine aminotransferase (OAT) to produce polyamines (putrescine, spermidine, and spermine) that contribute to *β*-cell dysfunction, insulin resistance, and pro-inflammatory responses, or by ornithine decarboxylase (ODC) to produce *L*-proline, which mediates *β*-cell dysfunction and insulin resistance. Both human patients and diabetic rats had considerably reduced plasma arginine concentrations, which may be positively correlated with arginase activation in diabetes mellitus. Experimental and clinical studies suggest that *L*-arginine supplementation may improve insulin secretion, insulin sensitivity, and glucose tolerance. Urea, a distinct byproduct of arginase-mediated *L*-arginine metabolism, has been linked to *β*-cell malfunction, decreased insulin sensitivity, and glucose intolerance (41). Hunger and diabetes mellitus are associated with higher levels of branched-chain amino acids (BCAAs), which include valine, leucine, and isoleucine. However, the pathophysiology is still unknown. Because of the high activity of BCAA aminotransferase, which converts BCAA and *α*-ketoglutarate (α-KG) into glutamate and branched-chain keto acids (BCKAs), research suggests that muscles are where most BCAA catabolism occurs (42). Consistent with the aforementioned findings, alanine, glutamic acid, branched-chain aromatic amino acids (BCAAs), aromatic amino acids (AAs), and α-aminobutyric acid were generally positively correlated with markers of glucose homeostasis, including fasting glucose, HbA1c, fasting insulin, C-peptide, hs-CRP, and HOMA of insulin resistance, whereas glycine, glutamine, asparagine, and taurine had an inverse relationship with these markers. Conversely, aspartic acid and glutamic acid were associated with a less favorable cardiometabolic profile (43). The pentose phosphate pathway is a key pathway for generating NADPH and 5-phosphoribose. In diabetic nephropathy, hyperglycemia and oxidative stress lead to excessive activation of the pentose phosphate pathway. Excessive production of NADPH may intensify the generation of reactive oxygen species and promote glomerular fibrosis and inflammation. Branched-chain amino acids and aromatic amino acids are often elevated in the plasma of patients with diabetic nephropathy, which may be related to insulin resistance and mitochondrial dysfunction. In the present investigation, the MOD group exhibited notably elevated levels of *L*-alanine, *L*-aspartate, *L*-leucine, *L*-valine, and *L*-isoleucine. The levels of *L*-alanine, *L*-aspartate, *L*-leucine, *L*-valine, and *L*-isoleucine were all significantly lowered by large YMP dosages. The high-dose group achieved significantly greater levels of *L*-arginine and citrulline. The therapeutic effect of YMP in MOD mice was not well concentration-dependent. Within a certain dosage range, a drug’s impact strength increases with administered dose. After the effect intensity reached its maximum efficacy, the dose increased but the effect stopped growing. Chinese drugs often showed no significant quantitative association in pharmacodynamic testing. The contents and intended purposes of Chinese remedies were complex. It is feasible for various parts of the same target organ to work on separate targets and interact with each other. Even though the majority of the composition’s elements were in the same class, there were many ambiguous elements, which indicated that even while the components were in the same class, the composition’s overall effect might differ slightly. However, because they may also have opposing effects, with one being excitatory and the other inhibitory, and because the thresholds of these components may also vary, the overall effects that were revealed at various levels were fairly complex. The interactions and modes of action of the formula’s component elements need further investigation. This study was the first to show that YMP corrected disruptions in the pentose phosphate pathway and amino acid metabolism, alleviated diabetes-induced pathological changes in the kidneys of diabetic mice, and had a regulating effect on the liver glycolipid metabolism. By investigating the novel pharmacological effect of traditional Chinese medicine and encouraging in-depth study and development, this work may offer a new experimental foundation and theoretical direction for the sensible application of YMP on DN.

## Data Availability

The original contributions presented in the study are included in the article/**Supplementary Material**. Further inquiries can be directed to the corresponding authors.

## Glossary

AAs: aromatic amino acids
AMPK: AMP-activated protein kinase
BCAAs: branched-chain amino acids
BP: biological processes
CAMs: cell adhesion molecules
CC: cellular components
CoA: coenzyme A
CON: control
CXCR3: CXC chemokine receptor 3
DEGs: differentially expressed genes
DN: Diabetic nephropathy
EMT: epithelial-to-mesenchymal transition
ESI: electrospray ionization
EtOH: ethyl alcohol
FA: ferulic acid
FC: fold change
FPKM: fragments per kilobase of transcript per million fragments mapped
G6PC: Glucose-6-phosphatase
G6PD: Glucose-6-phosphate 1-dehydrogenase
GAPDH: Glyceraldehyde-3-phosphate dehydrogenase
GLB: globulin
GO: gene ontology
GPI: GPI-anchor transamidase
GSH: glutathione
H&E: hematoxylin-eosin
HFD: high-fat (45%) diet
HK1: Hexokinase-1
HK2: Hexokinase-2
HK3: Hexokinase-3
HXLB: 3-hexulose-6-phosphate isomerase
IL-1*β*: interleukin-1*β*
IL-6: interleukin-6
KEGG: Kyoto Encyclopedia of Genes and Genomes
MDA: malondialdehyde
MET: metformin
MF: molecular functions
MOD: model
NEDD4L: E3 ubiquitin-protein ligase NEDD4-like
NO: nitric oxide
OMIM: Online Mendelian Inheritance in Man
OPLS-DA: Orthogonal partial least squares discriminant analysis
PCA: principal component analysis
PFKP: phosphofructokinase 1
PGLS: 6-phosphogluconolactonase
PPI: protein-protein interaction
RA: Rosmarinic acid
SOD: superoxide dismutase
STZ: streptozotocin
T-AOC: total antioxidant capacity
TCM: traditional Chinese medicine
TTD: Therapeutic Target Database
UmACR: urinary Microalbumin Creatinine Ratio
UPLC: Ultra Performance Liquid Chromatography
VIP: variable importance in projection
YMP: the peel of Zea mays L.
YMPH: high dose of YMP
YMPL: low dose of YMP
