# Supplementary material for: Network pharmacological investigation and experimental verification of the peel of Zea mays L. regulating metabolic reprogramming in the treatment of diabetic nephropathy
Source: Front Endocrinol (Lausanne). 2025 Jul 30;16:1594782. doi: 10.3389/fendo.2025.1594782 (PMC12343222; doi:10.3389/fendo.2025.1594782)
Supplement: Supplementary file 1 [file DataSheet1.docx]

Network pharmacological investigation and experimental verification of the peel of *Zea mays* L. regulating metabolic reprogramming in the treatment of diabetic nephropathy

Andong Wang ^a,#^, Jinyan He ^a,#^, Yuru Yang ^a^, Yaonan He ^a^, Guangtong Chen ^a^, Bai Ling ^a ,b^, Xiaotian Cheng ^a,b,*^

^a^ School of Pharmacy, Nantong University, Nantong, Jiangsu 226001, P. R. China

^b^ Department of Pharmacy, The Yancheng Clinical College of Xuzhou Medical University & The First people’s Hospital of Yancheng, Yancheng, Jiangsu 224001, P. R. China

# These authors contributed equally to this work and shared first authorship.

**Figure details**


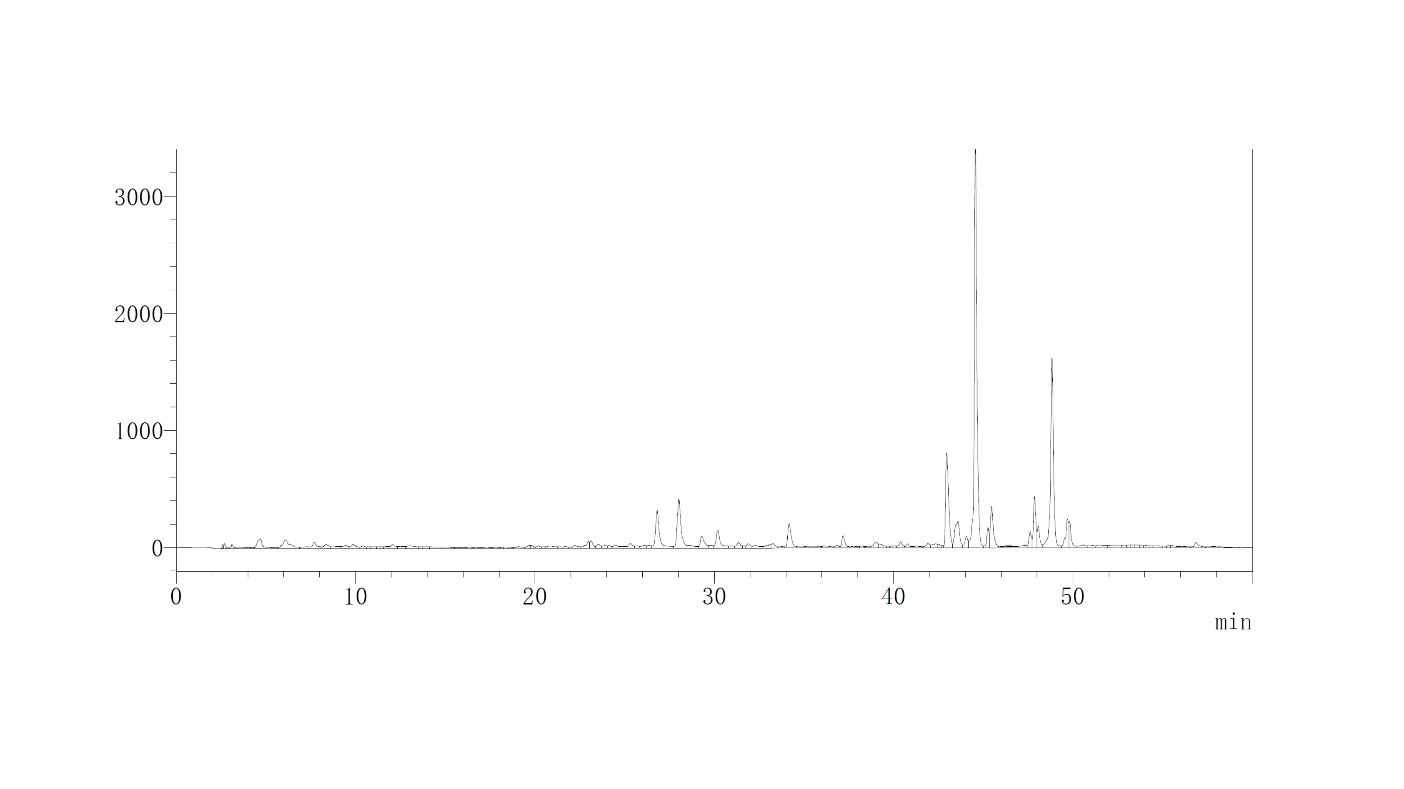


Figure S1 The High-performance Liquid Chromatography (HPLC) Spectrum of YMP


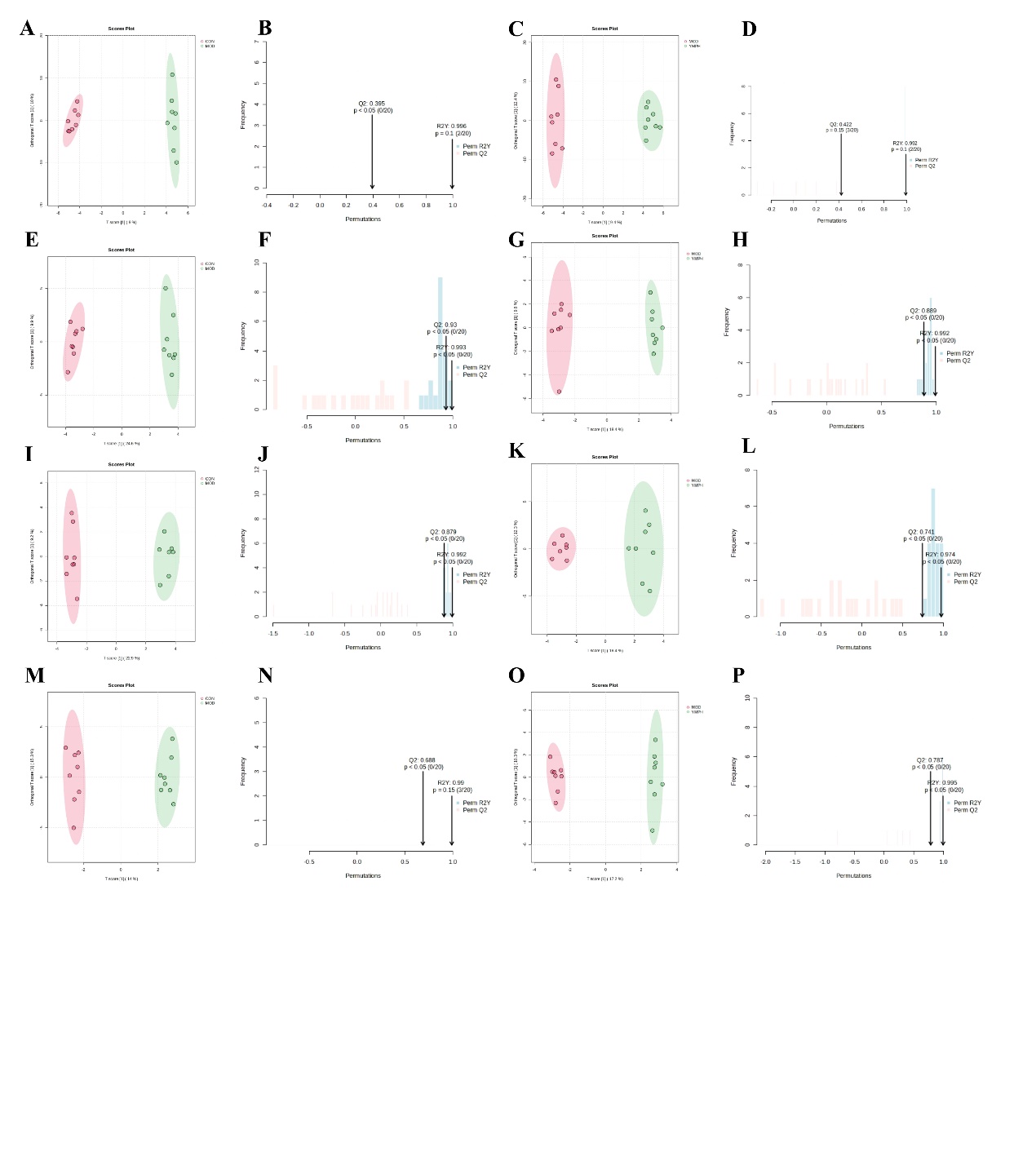


Figure S2 OPLS-DA between CON and MOD (A), R2Y and Q2 values between CON and MOD (B), OPLS-DA between MOD and YMPH (C), R2Y and Q2 values between MOD and YMPH (D), in positive serum mode, OPLS-DA between CON and MOD (E), R2Y and Q2 values between CON and MOD (F), OPLS-DA between MOD and YMPH (G), R2Y and Q2 values between MOD and YMPH (H), in negative serum mode, OPLS-DA between CON and MOD (I), R2Y and Q2 values between CON and MOD (J), OPLS-DA between MOD and YMPH (K), R2Y and Q2 values between MOD and YMPH (L), in positive urine mode, OPLS-DA between CON and MOD (M), R2Y and Q2 values between CON and MOD (N), OPLS-DA between MOD and YMPH (O), R2Y and Q2 values between MOD and YMPH (P), in negative urine mode.

Table S1 256 metabolites in positive mode in serum samples

| **ID** | **m/z** | **rt(s)** |
| --- | --- | --- |
| M409T212_1 | 408.65479 | 211.995500 |
| M292T409 | 292.10383 | 408.622000 |
| M876T492 | 876.39594 | 491.599500 |
| M605T213 | 604.99738 | 213.379000 |
| M641T213 | 640.99828 | 213.422000 |
| M928T364 | 928.13713 | 364.376000 |
| M424T372_1 | 423.99842 | 372.367000 |
| M424T372_2 | 424.01589 | 371.666000 |
| M225T243 | 224.92463 | 243.224500 |
| M683T480 | 683.10573 | 480.246000 |
| M246T430 | 246.14602 | 429.987000 |
| M376T401 | 376.02376 | 401.092000 |
| M310T470 | 309.87459 | 470.337000 |
| M417T472 | 417.17815 | 472.241000 |
| M617T358 | 617.00440 | 357.785500 |
| M721T365 | 721.14765 | 364.701000 |
| M528T438 | 527.97240 | 437.995000 |
| M502T214_2 | 502.31921 | 213.737000 |
| M539T460 | 538.97172 | 460.469000 |
| M482T433 | 482.02469 | 432.950000 |
| M326T357 | 326.14528 | 357.114500 |
| M298T293 | 298.02036 | 293.361000 |
| M448T295 | 448.05786 | 294.831000 |
| M490T428 | 490.02333 | 428.047000 |
| M223T306 | 222.89780 | 305.858000 |
| M668T366 | 668.12135 | 366.338000 |
| M634T434 | 633.98379 | 434.100500 |
| M521T367 | 521.07197 | 367.037000 |
| M502T398 | 502.10134 | 398.229500 |
| M629T438 | 628.98794 | 438.174500 |
| M587T472 | 587.20127 | 471.618000 |
| M453T357 | 453.17666 | 356.622500 |
| M509T597 | 509.02208 | 597.168500 |
| M312T303 | 312.13029 | 303.117000 |
| M432T407 | 432.04799 | 406.562500 |
| M129T417_4 | 129.36169 | 416.771000 |
| M465T429 | 464.98855 | 429.361000 |
| M627T490 | 627.17577 | 489.727000 |
| M678T213_2 | 678.33137 | 212.891000 |
| M263T342 | 262.98977 | 342.439000 |
| M563T303 | 562.93570 | 303.370000 |
| M533T486 | 533.11926 | 486.258000 |
| M427T304 | 426.99044 | 304.026000 |
| M653T302 | 652.84277 | 301.783000 |
| M213T426 | 213.08815 | 425.635000 |
| M361T415 | 361.08659 | 415.308000 |
| M499T214 | 498.65832 | 214.202000 |
| M499T213 | 499.32408 | 213.296000 |
| M413T461 | 413.09674 | 461.223000 |
| M803T365 | 803.15022 | 364.942000 |
| M457T304 | 456.88820 | 303.527000 |
| M429T460 | 428.96820 | 459.845000 |
| M467T412 | 467.01377 | 412.197000 |
| M371T366 | 371.10482 | 365.692000 |
| M665T302 | 664.76498 | 301.918000 |
| M804T213 | 804.00277 | 212.547000 |
| M405T357 | 404.99242 | 356.593000 |
| M293T344 | 292.89166 | 343.883000 |
| M783T461 | 783.20933 | 461.185000 |
| M937T467 | 936.92336 | 467.099500 |
| M476T440 | 476.07180 | 440.399000 |
| M660T213 | 659.99756 | 213.223000 |
| M302T416 | 302.14688 | 415.648000 |
| M421T254 | 421.27289 | 253.723000 |
| M520T214 | 519.98699 | 213.845000 |
| M294T431_1 | 293.97405 | 431.185000 |
| M1055T214_2 | 1055.34554 | 214.497000 |
| M307T306 | 306.96066 | 305.827500 |
| M269T592 | 268.90159 | 592.032000 |
| M267T403 | 267.09661 | 403.207000 |
| M415T435 | 414.97124 | 434.913000 |
| M541T303 | 540.94706 | 302.505500 |
| M366T402 | 366.02986 | 401.819500 |
| M555T214 | 554.65649 | 214.072500 |
| M300T214 | 300.26278 | 213.808000 |
| M573T304 | 572.86025 | 303.511500 |
| M692T372 | 692.07323 | 371.653500 |
| M243T612 | 242.89656 | 612.197500 |
| M505T461 | 505.11301 | 460.535000 |
| M330T287 | 330.05831 | 286.968000 |
| M466T357 | 466.12855 | 357.132000 |
| M918T212 | 917.51248 | 212.133500 |
| M266T304 | 266.08832 | 304.211500 |
| M920T595 | 919.54460 | 595.242500 |
| M570T434 | 569.99225 | 434.083000 |
| M329T292 | 329.00444 | 291.531000 |
| M509T430 | 509.03618 | 430.360000 |
| M161T305 | 161.06493 | 305.091000 |
| M550T373 | 550.04840 | 372.523500 |
| M705T340 | 705.06825 | 340.136500 |
| M507T501 | 506.87042 | 500.908000 |
| M345T306 | 344.99041 | 305.511500 |
| M102T214 | 101.92422 | 213.822500 |
| M215T215 | 215.12900 | 215.024500 |
| M407T404 | 407.12856 | 403.962500 |
| M244T406 | 244.16672 | 405.657000 |
| M392T427 | 392.04272 | 427.206000 |
| M482T507 | 481.79713 | 507.291000 |
| M517T426 | 517.03429 | 425.757000 |
| M845T365 | 845.13961 | 364.790000 |
| M716T372 | 716.03410 | 372.467000 |
| M402T447 | 402.05105 | 446.866500 |
| M321T211 | 321.24394 | 211.467000 |
| M701T304 | 700.84785 | 304.100000 |
| M781T214 | 781.00946 | 213.599000 |
| M522T372 | 522.04957 | 371.904000 |
| M242T462 | 242.07869 | 461.854000 |
| M609T368 | 609.08536 | 367.675000 |
| M378T293 | 378.02465 | 293.467500 |
| M264T433 | 264.04661 | 433.051000 |
| M679T213 | 678.99699 | 213.232000 |
| M521T343 | 521.02212 | 343.163000 |
| M423T502 | 422.83065 | 502.079500 |
| M480T213 | 479.99161 | 212.754000 |
| M948T213 | 947.75733 | 212.510000 |
| M367T303 | 366.96879 | 303.022000 |
| M367T304 | 366.98180 | 303.530000 |
| M379T412 | 378.99353 | 412.330500 |
| M862T595 | 861.58366 | 594.637000 |
| M483T502 | 482.78132 | 502.071500 |
| M199T215 | 199.07253 | 215.090000 |
| M246T443 | 246.14602 | 442.779000 |
| M709T345 | 708.93953 | 345.032000 |
| M699T214 | 698.66167 | 213.506000 |
| M310T453 | 309.95936 | 452.781500 |
| M205T452 | 204.90410 | 452.101000 |
| M413T431 | 413.01682 | 431.323000 |
| M350T404 | 350.04496 | 403.971000 |
| M624T214 | 623.99667 | 213.865500 |
| M363T448 | 363.12628 | 447.608000 |
| M621T302 | 620.84311 | 302.464000 |
| M516T430 | 516.01411 | 430.187500 |
| M781T358 | 781.01074 | 357.503000 |
| M432T448 | 432.13420 | 447.574000 |
| M350T447 | 350.13098 | 447.047500 |
| M370T303 | 370.13680 | 303.089500 |
| M82T593 | 81.71790 | 593.356000 |
| M492T357 | 492.03870 | 356.850000 |
| M443T429 | 443.00696 | 429.303000 |
| M434T213 | 434.49101 | 213.223000 |
| M493T447 | 492.99065 | 447.159500 |
| M799T304 | 798.82468 | 304.111500 |
| M869T367 | 869.10030 | 366.803000 |
| M381T306 | 381.00623 | 306.437000 |
| M381T593 | 380.93507 | 592.676000 |
| M108T472 | 108.05677 | 472.347000 |
| M769T213_2 | 769.33278 | 213.046000 |
| M979T213 | 979.01538 | 213.171000 |
| M104T597 | 104.40419 | 597.200500 |
| M453T358 | 452.99823 | 357.708000 |
| M925T461 | 925.14108 | 461.103000 |
| M402T504 | 401.81204 | 504.174500 |
| M551T592 | 551.36652 | 592.012500 |
| M523T342 | 523.00122 | 342.456000 |
| M422T416 | 422.05341 | 416.463000 |
| M388T597 | 388.02983 | 596.510000 |
| M210T513 | 209.89340 | 512.829500 |
| M441T453 | 440.93719 | 452.757500 |
| M259T446 | 259.14133 | 445.620000 |
| M405T305 | 404.93772 | 305.057000 |
| M96T215 | 95.93457 | 215.499500 |
| M460T507 | 459.81520 | 507.157000 |
| M715T595 | 714.95527 | 594.591500 |
| M607T213 | 606.99420 | 213.477500 |
| M322T428 | 322.06354 | 428.155000 |
| M359T346 | 358.94594 | 345.869000 |
| M260T487 | 259.88974 | 487.441000 |
| M697T594 | 697.46301 | 593.950000 |
| M235T590 | 235.10112 | 590.083000 |
| M394T432 | 394.00448 | 431.987500 |
| M611T593 | 610.96293 | 592.641000 |
| M466T507 | 465.82331 | 507.010500 |
| M359T303 | 358.91089 | 302.646500 |
| M838T214_2 | 837.67401 | 213.511000 |
| M182T342 | 182.05720 | 342.385000 |
| M423T303 | 422.89112 | 303.262000 |
| M736T357 | 736.09919 | 356.974000 |
| M400T507 | 399.86466 | 506.828000 |
| M98T404 | 98.06119 | 403.867500 |
| M501T520 | 500.77364 | 520.234000 |
| M256T489 | 256.13028 | 488.585500 |
| M776T596 | 775.50994 | 595.851000 |
| M344T448 | 344.11413 | 447.596000 |
| M272T213 | 272.23138 | 213.204000 |
| M199T507 | 199.42882 | 506.561000 |
| M444T507_2 | 443.84242 | 507.041000 |
| M514T447 | 514.13719 | 447.094000 |
| M529T452 | 528.95847 | 452.192500 |
| M512T453 | 511.91483 | 452.640500 |
| M441T345 | 440.94892 | 345.002000 |
| M1032T212 | 1031.68619 | 212.399500 |
| M314T306 | 313.94669 | 305.982000 |
| M424T504 | 423.79368 | 503.981500 |
| M523T345 | 522.95220 | 345.002000 |
| M244T429 | 244.09403 | 429.415000 |
| M439T344 | 439.01892 | 344.434000 |
| M305T593 | 304.92804 | 593.321000 |
| M78T335 | 77.72044 | 334.993000 |
| M697T213 | 696.99686 | 212.653000 |
| M208T449 | 208.10696 | 448.730500 |
| M258T448 | 258.14610 | 448.090000 |
| M529T498 | 528.86518 | 498.304000 |
| M385T213 | 385.48137 | 212.853500 |
| M267T600 | 266.94123 | 599.683000 |
| M698T594 | 698.46818 | 593.965000 |
| M111T213 | 110.91692 | 213.163000 |
| M443T305 | 442.96779 | 305.104500 |
| M487T513 | 486.75244 | 512.912000 |
| M203T452 | 202.90333 | 452.160500 |
| M597T213 | 597.49519 | 213.355000 |
| M282T488 | 282.10739 | 488.231000 |
| M640T214 | 640.33284 | 214.198000 |
| M487T344 | 486.97435 | 344.462500 |
| M269T597 | 268.99690 | 597.230000 |
| M451T343 | 450.97998 | 343.438000 |
| M596T214 | 596.49645 | 213.569500 |
| M320T507 | 319.87812 | 506.839500 |
| M627T593 | 626.93591 | 593.310500 |
| M341T356 | 341.01656 | 356.335000 |
| M78T303 | 77.72042 | 303.095000 |
| M569T344 | 568.97712 | 344.142000 |
| M202T452 | 201.90459 | 451.550000 |
| M133T215_2 | 133.05073 | 215.407500 |
| M627T345 | 626.93643 | 344.516000 |
| M535T358 | 535.00153 | 358.365000 |
| M357T344 | 357.01535 | 343.995000 |
| M398T519 | 397.84040 | 518.798000 |
| M441T344 | 440.99840 | 344.073000 |
| M384T507 | 383.81982 | 507.158000 |
| M585T513 | 584.72804 | 513.375000 |
| M460T518 | 459.79647 | 518.150000 |
| M544T204 | 544.30461 | 203.794000 |
| M168T396 | 168.07778 | 395.640500 |
| M168T471 | 168.07795 | 470.997500 |
| M452T197 | 452.27840 | 197.431500 |
| M793T216 | 792.53149 | 216.489000 |
| M558T223 | 558.33411 | 223.318000 |
| M793T150 | 792.57841 | 150.343000 |
| M245T156 | 245.11835 | 155.834000 |
| M168T423 | 168.02677 | 422.811000 |
| M237T219 | 237.09155 | 219.191000 |
| M164T130 | 164.03521 | 130.071000 |
| M218T238 | 218.09359 | 238.191000 |
| M186T447 | 186.12487 | 447.094000 |
| M113T416 | 113.02448 | 415.943500 |
| M113T388 | 113.03568 | 387.690000 |
| M291T464 | 291.08349 | 464.148000 |
| M274T455 | 274.10459 | 454.745000 |
| M293T365 | 293.11445 | 364.844000 |
| M154T395 | 154.06210 | 395.194000 |
| M188T210 | 188.03532 | 210.220000 |
| M425T503 | 425.08100 | 502.626000 |
| M377T93 | 377.20032 | 92.947100 |
| M128T423 | 128.03536 | 422.506000 |
| M738T162 | 737.53757 | 161.519500 |
| M469T165 | 469.22696 | 164.838000 |

Table S2 273 metabolites in negative mode in serum samples

| **ID** | **m/z** | **rt(s)** |
| --- | --- | --- |
| M445T367 | 445.07966 | 366.935500 |
| M481T383 | 481.11493 | 383.479000 |
| M787T479 | 787.00510 | 478.731000 |
| M569T250 | 569.13368 | 250.257000 |
| M243T358 | 243.13506 | 357.668000 |
| M408T241 | 407.97540 | 241.181000 |
| M401T366_2 | 401.10282 | 365.682000 |
| M401T366_1 | 401.04992 | 365.789500 |
| M496T351_2 | 496.17394 | 350.613500 |
| M307T387 | 307.04599 | 387.279500 |
| M310T426 | 310.04000 | 426.208000 |
| M562T507 | 561.76122 | 506.848000 |
| M977T212 | 976.68574 | 212.047000 |
| M467T488 | 467.09279 | 488.309500 |
| M810T370 | 810.06369 | 370.104000 |
| M461T368 | 461.07442 | 367.552000 |
| M700T242 | 700.32052 | 242.253000 |
| M361T459 | 360.83642 | 459.253000 |
| M1034T213_2 | 1034.34894 | 212.890000 |
| M475T435 | 474.95286 | 435.209500 |
| M437T599 | 437.03712 | 598.502000 |
| M315T469 | 314.84778 | 468.944000 |
| M673T341 | 672.97130 | 341.415500 |
| M129T417_3 | 129.32468 | 416.737000 |
| M98T214 | 97.93138 | 213.808000 |
| M300T427 | 300.08169 | 427.159000 |
| M704T366 | 704.09962 | 366.500000 |
| M198T434 | 197.98673 | 434.456000 |
| M353T257 | 352.96255 | 257.320000 |
| M643T336 | 642.98206 | 336.460000 |
| M412T337 | 411.99088 | 337.282500 |
| M783T459 | 783.04917 | 458.646000 |
| M430T419 | 430.09454 | 418.807500 |
| M296T432 | 295.95309 | 431.777000 |
| M563T514 | 562.74552 | 513.768500 |
| M269T472 | 268.90143 | 471.949000 |
| M867T302 | 866.79174 | 302.473000 |
| M773T467 | 773.01325 | 467.056000 |
| M436T385 | 436.09273 | 384.857000 |
| M381T212 | 381.48761 | 212.037000 |
| M671T303 | 670.83722 | 303.105000 |
| M259T537 | 258.92666 | 537.375500 |
| M304T370 | 304.03953 | 369.700000 |
| M735T465 | 734.81679 | 465.097500 |
| M162T237 | 161.93580 | 236.887000 |
| M928T212_2 | 928.34288 | 212.077000 |
| M666T485 | 665.80579 | 485.317500 |
| M769T303 | 768.81473 | 302.713000 |
| M304T309 | 304.03464 | 309.250000 |
| M324T482 | 324.12900 | 482.280000 |
| M220T305 | 220.01344 | 305.225000 |
| M404T401 | 404.10457 | 400.772000 |
| M184T210 | 184.09799 | 210.428000 |
| M621T393 | 621.22066 | 392.626000 |
| M975T367 | 975.14725 | 367.179000 |
| M906T212 | 905.75784 | 211.886000 |
| M575T598 | 574.99384 | 598.495500 |
| M435T212 | 435.48963 | 212.346000 |
| M581T369 | 581.08660 | 369.361000 |
| M202T282 | 202.07220 | 281.652000 |
| M663T302 | 662.76732 | 302.363500 |
| M370T353 | 370.05451 | 352.562500 |
| M623T340 | 623.06521 | 340.375500 |
| M266T412 | 266.02534 | 411.919000 |
| M343T290 | 343.04968 | 290.339000 |
| M339T503 | 338.89283 | 503.495000 |
| M275T588 | 274.88067 | 587.593000 |
| M238T238 | 237.91660 | 238.073500 |
| M249T426 | 249.10923 | 426.019000 |
| M447T452 | 446.95503 | 452.176000 |
| M282T405 | 282.12086 | 404.897500 |
| M477T403 | 477.18161 | 403.190000 |
| M198T228 | 197.94237 | 228.458500 |
| M618T404 | 618.13469 | 404.154000 |
| M274T237 | 274.08358 | 236.825000 |
| M185T393 | 185.05673 | 393.067500 |
| M837T338 | 836.97750 | 338.013000 |
| M307T402 | 306.95793 | 401.622000 |
| M693T371 | 693.06844 | 370.819500 |
| M747T304 | 746.82951 | 304.289000 |
| M320T369 | 320.03424 | 368.923000 |
| M588T213 | 587.99540 | 212.825000 |
| M731T212_1 | 730.66890 | 211.759000 |
| M282T462 | 282.07098 | 461.868000 |
| M321T581 | 320.90122 | 580.919500 |
| M248T431 | 247.97110 | 430.600000 |
| M727T365 | 727.15681 | 365.204000 |
| M129T417_2 | 129.28748 | 416.591000 |
| M677T213 | 676.99967 | 212.747000 |
| M296T294 | 296.02177 | 294.084500 |
| M318T470 | 317.84831 | 469.725000 |
| M285T359 | 285.03972 | 358.876000 |
| M599T364 | 599.13033 | 363.556000 |
| M370T580 | 369.83493 | 580.264000 |
| M792T595 | 791.51802 | 595.258000 |
| M901T209 | 900.53146 | 209.335000 |
| M505T480 | 505.08456 | 479.825000 |
| M425T532 | 424.89884 | 532.331500 |
| M982T214 | 982.34329 | 213.673000 |
| M473T466 | 472.93509 | 466.066000 |
| M347T415 | 347.07119 | 415.058500 |
| M311T393 | 311.10990 | 392.638500 |
| M414T286 | 414.12366 | 285.674000 |
| M338T412 | 338.03506 | 412.252000 |
| M1119T213 | 1119.27004 | 212.660000 |
| M1146T214 | 1145.77281 | 214.424500 |
| M699T364 | 699.16079 | 364.398500 |
| M160T215 | 160.06165 | 214.569000 |
| M729T340 | 729.02915 | 339.939000 |
| M261T453_2 | 260.95385 | 452.831000 |
| M124T242 | 124.05174 | 241.724000 |
| M314T425 | 314.09730 | 424.944000 |
| M448T404 | 448.15525 | 403.943000 |
| M642T212 | 642.32943 | 212.275000 |
| M634T372 | 634.03169 | 371.942000 |
| M364T371 | 364.04730 | 371.031000 |
| M174T238 | 173.88582 | 237.707000 |
| M158T507 | 158.43349 | 506.868500 |
| M214T410 | 214.07222 | 410.100500 |
| M1036T213 | 1035.68036 | 213.000000 |
| M537T469 | 536.90806 | 469.322000 |
| M469T597 | 468.94607 | 597.116000 |
| M425T295 | 425.07353 | 294.535000 |
| M440T372 | 440.04650 | 371.546000 |
| M322T310 | 322.07854 | 309.668000 |
| M281T602 | 280.97876 | 601.565000 |
| M299T538 | 298.93175 | 537.932000 |
| M289T591 | 288.90907 | 591.391000 |
| M477T360 | 477.04542 | 359.781500 |
| M335T335 | 335.22065 | 334.908000 |
| M229T361 | 229.08080 | 361.492000 |
| M496T401 | 496.17833 | 400.571000 |
| M490T453 | 489.93270 | 452.831000 |
| M809T478 | 808.98644 | 477.557000 |
| M482T213 | 481.98849 | 213.026000 |
| M351T504_1 | 350.86042 | 504.445000 |
| M507T592 | 506.90208 | 591.657000 |
| M527T367 | 527.08265 | 367.384500 |
| M980T212_2 | 980.34703 | 212.282000 |
| M258T614 | 257.92475 | 613.951500 |
| M445T461 | 445.18406 | 460.560000 |
| M190T431 | 189.98913 | 431.422000 |
| M339T338 | 339.23283 | 338.136000 |
| M633T595_2 | 633.03564 | 594.633000 |
| M658T213 | 657.99983 | 212.715000 |
| M491T496 | 491.21082 | 495.660000 |
| M259T578 | 258.92666 | 577.931000 |
| M389T472 | 389.17194 | 472.267500 |
| M471T357 | 471.04760 | 356.713500 |
| M752T369 | 752.10635 | 368.595000 |
| M72T378 | 72.13559 | 378.467000 |
| M664T466 | 663.97254 | 465.724000 |
| M343T401 | 343.01377 | 401.399500 |
| M387T603 | 386.94302 | 602.816000 |
| M360T309 | 360.03476 | 309.440000 |
| M383T589 | 382.86966 | 588.784000 |
| M375T308 | 374.99188 | 307.948000 |
| M580T365 | 580.10845 | 364.603000 |
| M483T367_1 | 483.03468 | 366.818000 |
| M605T343 | 605.00415 | 342.829500 |
| M605T345 | 604.95544 | 345.036000 |
| M445T598 | 444.98493 | 598.290000 |
| M579T479 | 579.10864 | 479.244500 |
| M337T506 | 336.86888 | 506.460000 |
| M192T239 | 191.85809 | 238.656000 |
| M568T434 | 567.99349 | 434.184000 |
| M533T435_1 | 532.96819 | 435.274000 |
| M444T405 | 444.11023 | 404.751000 |
| M1019T214 | 1019.34443 | 213.803000 |
| M102T471 | 101.93383 | 470.579500 |
| M156T356 | 156.04156 | 355.981500 |
| M492T213 | 492.48684 | 212.855000 |
| M313T614 | 312.90856 | 613.889000 |
| M663T459 | 663.10034 | 458.714000 |
| M506T392 | 506.14622 | 392.354000 |
| M398T496 | 398.16821 | 495.580000 |
| M685T461 | 685.08326 | 460.573000 |
| M593T358 | 593.04321 | 357.785500 |
| M433T257 | 433.03035 | 257.175000 |
| M244T405 | 244.08278 | 405.391000 |
| M640T365 | 640.12371 | 364.758500 |
| M387T345_1 | 386.94309 | 345.291000 |
| M733T213 | 732.66667 | 213.355000 |
| M765T478 | 765.02262 | 477.533000 |
| M300T405 | 300.06677 | 404.946000 |
| M661T213 | 661.32892 | 213.028000 |
| M657T597 | 656.99663 | 596.936000 |
| M298T615 | 297.81704 | 614.825000 |
| M248T366 | 248.09300 | 365.656500 |
| M411T212_1 | 410.65197 | 212.403500 |
| M635T343_2 | 635.00349 | 343.187000 |
| M291T451 | 291.16756 | 450.608500 |
| M661T303 | 660.91231 | 302.947500 |
| M355T582 | 354.87021 | 581.625000 |
| M192T216 | 191.81658 | 215.665000 |
| M492T367 | 492.07849 | 366.889500 |
| M485T345 | 484.97829 | 344.890000 |
| M402T294 | 402.06958 | 294.343000 |
| M260T441 | 259.95513 | 440.987500 |
| M280T284 | 280.06232 | 283.937000 |
| M284T294_1 | 283.90763 | 294.446000 |
| M193T344 | 193.07190 | 343.544000 |
| M321T469 | 320.85024 | 469.136000 |
| M925T212 | 925.01366 | 211.844000 |
| M444T213 | 443.99015 | 213.064000 |
| M458T442 | 458.23732 | 442.472000 |
| M811T366 | 811.14427 | 366.160000 |
| M390T428 | 389.99274 | 427.567000 |
| M270T392 | 270.07112 | 392.200500 |
| M321T293 | 321.06894 | 293.413000 |
| M384T287 | 384.11279 | 286.965000 |
| M1067T213 | 1067.26527 | 213.232000 |
| M699T358 | 699.00756 | 357.852000 |
| M113T401 | 113.06089 | 400.526000 |
| M401T471 | 401.20070 | 471.014000 |
| M632T426 | 632.06518 | 425.993500 |
| M820T595 | 819.53669 | 594.601000 |
| M229T519 | 229.39455 | 518.649000 |
| M403T430 | 402.98784 | 429.535000 |
| M511T459 | 510.97138 | 459.241000 |
| M642T213 | 641.66396 | 212.873000 |
| M611T301 | 610.91269 | 301.361500 |
| M419T585 | 418.88081 | 584.872500 |
| M100T216 | 100.04048 | 215.671000 |
| M407T459 | 406.98620 | 459.214000 |
| M480T468 | 479.95248 | 467.751000 |
| M444T443 | 444.02687 | 442.548500 |
| M87T389 | 87.05640 | 389.422500 |
| M661T212 | 660.66325 | 212.442500 |
| M514T213 | 513.99588 | 212.528500 |
| M840T213_2 | 839.75327 | 212.541000 |
| M398T354 | 398.05338 | 354.042000 |
| M916T370 | 916.11146 | 369.578000 |
| M430T213 | 429.65119 | 213.085500 |
| M497T435 | 496.93459 | 434.763000 |
| M1107T213 | 1106.76753 | 212.860500 |
| M281T530 | 280.92112 | 530.012000 |
| M384T212 | 384.48321 | 212.344000 |
| M391T399 | 391.09997 | 398.739000 |
| M430T438 | 429.99185 | 437.965500 |
| M348T293 | 348.01395 | 293.469000 |
| M272T444 | 272.08841 | 444.395000 |
| M621T592 | 621.43168 | 592.019000 |
| M419T426_2 | 419.05343 | 425.999000 |
| M199T434 | 199.07247 | 434.256000 |
| M947T213_1 | 946.67522 | 212.547000 |
| M336T577 | 335.88478 | 577.211000 |
| M354T287 | 354.10208 | 287.041000 |
| M465T303 | 464.95905 | 303.262000 |
| M339T257 | 338.98228 | 256.950000 |
| M957T301 | 956.69844 | 301.293000 |
| M423T597 | 422.91981 | 596.556000 |
| M438T213 | 438.48506 | 212.782500 |
| M153T213 | 153.06705 | 212.846000 |
| M190T237 | 189.86152 | 237.319000 |
| M696T212_1 | 695.66591 | 212.291000 |
| M469T345 | 468.94629 | 344.899000 |
| M465T394 | 465.11957 | 394.065000 |
| M254T450 | 254.11244 | 449.973000 |
| M263T602 | 263.01309 | 601.892500 |
| M756T303 | 755.54169 | 303.254000 |
| M588T367 | 588.10684 | 367.372000 |
| M266T461 | 266.11242 | 461.198000 |
| M586T365 | 586.11880 | 364.562000 |
| M161T345 | 160.93558 | 345.182000 |
| M379T426 | 379.01699 | 425.968000 |
| M965T303 | 964.76729 | 302.810000 |
| M487T213 | 487.49419 | 212.753000 |
| M375T450 | 375.13889 | 449.599000 |
| M488T239 | 488.14666 | 238.540000 |
| M385T408 | 385.11409 | 408.038000 |
| M710T610 | 709.96491 | 609.708000 |
| M422T507 | 421.84655 | 507.187000 |

Table S3 427 metabolites in positive mode in urine samples

| **ID** | **m/z** | **rt(s)** |
| --- | --- | --- |
| M381T417 | 381.00928 | 416.906000 |
| M267T304_1 | 267.07235 | 303.878500 |
| M289T488 | 289.11567 | 488.120000 |
| M258T472 | 258.08208 | 471.616500 |
| M704T383 | 704.16569 | 382.730000 |
| M789T300_2 | 788.88622 | 299.919000 |
| M502T214_1 | 501.65371 | 213.937000 |
| M245T473 | 244.96115 | 473.249500 |
| M465T513 | 464.77053 | 513.181000 |
| M172T367 | 172.07282 | 367.297500 |
| M332T213 | 332.47859 | 213.104000 |
| M400T444 | 400.02153 | 443.633000 |
| M468T455 | 467.95059 | 455.383000 |
| M229T343 | 229.04825 | 343.293000 |
| M346T377 | 346.11964 | 377.420500 |
| M1104T475 | 1104.48009 | 474.911000 |
| M294T615 | 293.82290 | 615.310000 |
| M179T464 | 178.88439 | 463.827500 |
| M765T212 | 764.67281 | 211.683000 |
| M359T589 | 358.94403 | 588.790500 |
| M610T372 | 610.06927 | 371.546000 |
| M926T213 | 926.34512 | 213.291500 |
| M530T428 | 530.03007 | 428.094000 |
| M161T486 | 161.09326 | 486.286500 |
| M764T212 | 764.00692 | 212.108000 |
| M294T285 | 294.04240 | 284.959500 |
| M537T300 | 536.88210 | 299.632000 |
| M893T365 | 893.14514 | 365.482500 |
| M316T470 | 315.84865 | 469.655000 |
| M203T304 | 203.09390 | 304.289000 |
| M451T435 | 450.96522 | 435.295000 |
| M438T285 | 438.21597 | 285.228000 |
| M438T282 | 438.19781 | 282.347000 |
| M770T367 | 769.86169 | 367.197500 |
| M729T212 | 729.33670 | 212.430000 |
| M426T212 | 425.65730 | 211.742000 |
| M446T438 | 445.96912 | 437.901000 |
| M255T440 | 254.99084 | 440.397000 |
| M267T435 | 266.99814 | 435.140000 |
| M681T254 | 681.32103 | 253.577000 |
| M340T370 | 340.01564 | 369.564000 |
| M418T366 | 418.12927 | 365.693000 |
| M561T438 | 561.00020 | 437.665000 |
| M836T299 | 835.88118 | 299.174500 |
| M425T591 | 424.89891 | 591.374500 |
| M861T301 | 860.71918 | 301.388000 |
| M201T283_1 | 200.92186 | 283.013500 |
| M1034T213_1 | 1033.68361 | 213.463000 |
| M1091T213 | 1091.27086 | 213.379000 |
| M606T212 | 606.32917 | 212.456500 |
| M431T393 | 431.12185 | 392.620000 |
| M329T412 | 328.98604 | 411.863500 |
| M374T446 | 373.97940 | 446.282000 |
| M539T246 | 539.19561 | 245.676000 |
| M436T467 | 435.77847 | 467.041000 |
| M569T354 | 569.10403 | 353.553000 |
| M70T478 | 70.20120 | 477.511500 |
| M278T372 | 278.08826 | 372.372000 |
| M448T403 | 448.10773 | 403.177500 |
| M1010T213 | 1010.01495 | 213.134000 |
| M763T302 | 762.74200 | 301.852500 |
| M646T349 | 646.03570 | 348.896000 |
| M1013T301 | 1012.75127 | 301.207000 |
| M292T428 | 292.03108 | 427.971500 |
| M1015T212 | 1014.68447 | 212.022000 |
| M242T432 | 242.04696 | 431.882500 |
| M339T439 | 339.11708 | 439.286500 |
| M361T429 | 361.00363 | 429.315000 |
| M769T474_2 | 769.34991 | 474.276000 |
| M479T521 | 478.79100 | 520.681000 |
| M505T471 | 505.19820 | 470.959000 |
| M147T300 | 146.92621 | 300.299000 |
| M260T488 | 260.12537 | 487.581500 |
| M267T304_2 | 267.09653 | 304.353000 |
| M784T213 | 784.00504 | 212.742000 |
| M427T436 | 427.12681 | 436.298500 |
| M903T300 | 902.84490 | 300.424500 |
| M635T227 | 635.09528 | 226.651000 |
| M384T422 | 384.05251 | 421.762000 |
| M129T362 | 129.10344 | 362.203000 |
| M251T593 | 250.98161 | 593.316500 |
| M226T356 | 226.08188 | 356.095000 |
| M1106T213_2 | 1106.26883 | 213.444000 |
| M294T310 | 294.04618 | 309.931000 |
| M242T457 | 241.85093 | 456.654000 |
| M307T492 | 307.15124 | 492.270500 |
| M905T364 | 905.25408 | 364.448000 |
| M1105T213 | 1105.26888 | 213.097000 |
| M376T215 | 376.18809 | 214.545000 |
| M243T340 | 243.06011 | 340.173000 |
| M359T585 | 359.01469 | 585.180000 |
| M626T422 | 626.13952 | 422.159000 |
| M868T386 | 868.29229 | 385.993000 |
| M316T431 | 316.09515 | 430.558500 |
| M1048T213 | 1048.02370 | 212.641000 |
| M283T580 | 283.35818 | 580.212000 |
| M1033T335 | 1032.93322 | 334.594500 |
| M409T294 | 409.09603 | 294.159000 |
| M252T431 | 251.98932 | 431.330500 |
| M254T310_2 | 254.08243 | 309.923000 |
| M391T473 | 391.10744 | 472.846500 |
| M115T292 | 115.02092 | 291.821000 |
| M692T212 | 692.00497 | 212.285000 |
| M108T310 | 107.99662 | 309.859000 |
| M280T355 | 280.05272 | 355.013000 |
| M353T287 | 353.05702 | 286.616500 |
| M301T479 | 301.03472 | 479.498000 |
| M125T361 | 125.09720 | 361.492000 |
| M521T475 | 520.74980 | 474.901000 |
| M1132T212 | 1132.02219 | 211.645000 |
| M483T367_2 | 483.05419 | 367.342000 |
| M717T386 | 717.22634 | 385.993000 |
| M681T342 | 680.95829 | 341.983500 |
| M541T340 | 541.06203 | 340.351000 |
| M499T366 | 499.08354 | 366.365000 |
| M321T533 | 320.90130 | 533.279000 |
| M340T415 | 340.05031 | 415.306500 |
| M169T215 | 169.06197 | 214.869000 |
| M85T423 | 84.93875 | 422.517500 |
| M240T352 | 240.06040 | 351.567000 |
| M251T356 | 251.04112 | 356.232000 |
| M105T299 | 104.53495 | 299.138500 |
| M606T213 | 605.66304 | 212.986500 |
| M529T594 | 528.96712 | 593.967000 |
| M401T365_1 | 401.03235 | 365.373000 |
| M336T539 | 335.88482 | 538.712500 |
| M299T453 | 298.92200 | 452.834500 |
| M895T477 | 895.30281 | 477.267000 |
| M214T374 | 214.10859 | 374.495000 |
| M698T213 | 697.99588 | 212.635000 |
| M272T304 | 272.01788 | 304.360000 |
| M262T292 | 262.09340 | 291.958000 |
| M512T351 | 512.15156 | 351.060000 |
| M361T338 | 361.06049 | 337.614500 |
| M273T485 | 273.09438 | 485.400000 |
| M253T356 | 253.05657 | 356.387000 |
| M243T294 | 243.09866 | 293.957000 |
| M300T440 | 300.12032 | 439.854000 |
| M713T464 | 712.83614 | 464.454000 |
| M305T397 | 305.06452 | 396.782500 |
| M508T432 | 508.01577 | 431.777000 |
| M185T215 | 185.05686 | 215.100500 |
| M617T364 | 617.15780 | 364.195000 |
| M801T213 | 801.34010 | 212.949000 |
| M177T365 | 177.07805 | 364.884000 |
| M427T430 | 427.03279 | 429.873000 |
| M235T233 | 235.10886 | 233.152000 |
| M458T365 | 458.09034 | 364.675000 |
| M379T389 | 379.09145 | 388.658500 |
| M385T478 | 385.09064 | 478.387000 |
| M510T337 | 510.02548 | 337.023500 |
| M335T284 | 335.10167 | 284.095000 |
| M377T581 | 376.86171 | 580.602000 |
| M363T365 | 363.07594 | 365.155000 |
| M339T423_1 | 339.04774 | 422.949000 |
| M553T211 | 552.65963 | 211.405500 |
| M319T364 | 319.09968 | 364.324500 |
| M311T468 | 310.87389 | 468.403000 |
| M359T308 | 359.01448 | 308.386500 |
| M805T459 | 805.03267 | 458.709000 |
| M317T352 | 317.07796 | 351.531000 |
| M382T212 | 382.48605 | 212.153000 |
| M531T249 | 531.16840 | 249.293000 |
| M596T253 | 596.31371 | 252.630000 |
| M445T469 | 444.92636 | 469.064000 |
| M769T213_1 | 768.66719 | 213.227000 |
| M230T253 | 230.08841 | 252.926000 |
| M943T213_1 | 942.68069 | 212.648000 |
| M982T213 | 981.67815 | 213.046000 |
| M265T422 | 265.14807 | 422.131000 |
| M261T228 | 260.99173 | 228.016000 |
| M282T471 | 282.02831 | 470.667000 |
| M418T294 | 418.04669 | 294.343000 |
| M284T310 | 284.05189 | 310.382500 |
| M359T528 | 358.94383 | 527.526500 |
| M197T370 | 196.90354 | 369.952500 |
| M354T211 | 353.98734 | 211.435000 |
| M205T472 | 205.05389 | 472.271500 |
| M713T429 | 713.04759 | 429.339000 |
| M396T362 | 396.13661 | 362.040000 |
| M372T453 | 372.03485 | 452.821000 |
| M626T247_1 | 626.20691 | 247.356000 |
| M274T393 | 274.01347 | 392.532000 |
| M681T365 | 681.13328 | 364.564000 |
| M244T358 | 244.04624 | 358.165000 |
| M463T417 | 463.01241 | 416.850500 |
| M905T300 | 904.84829 | 300.111000 |
| M641T350 | 641.15833 | 350.468000 |
| M312T294 | 311.99963 | 294.446000 |
| M803T212_2 | 803.33655 | 211.912500 |
| M694T213_2 | 694.33445 | 212.713000 |
| M861T466 | 860.78171 | 465.642000 |
| M512T447 | 511.99983 | 446.932000 |
| M263T411 | 263.03915 | 410.889000 |
| M1095T423 | 1095.11020 | 422.535500 |
| M925T299 | 924.83438 | 299.462000 |
| M709T300 | 708.89096 | 300.301500 |
| M310T427 | 309.96887 | 427.205000 |
| M345T427 | 345.07915 | 426.605000 |
| M861T423 | 861.03633 | 423.152000 |
| M405T478 | 405.02958 | 478.199000 |
| M403T531 | 402.90425 | 531.379000 |
| M169T282 | 169.06197 | 281.623000 |
| M513T479 | 513.08319 | 479.383000 |
| M425T453 | 424.87950 | 453.413000 |
| M864T595 | 863.59786 | 594.611000 |
| M359T403 | 359.12502 | 403.113500 |
| M211T615 | 210.84231 | 615.148000 |
| M477T294 | 477.06579 | 294.212000 |
| M379T247 | 379.00233 | 246.800500 |
| M1123T423 | 1123.11273 | 423.059500 |
| M240T238 | 239.91291 | 237.614000 |
| M563T368 | 563.05926 | 368.243000 |
| M224T230 | 223.79834 | 229.577000 |
| M460T365 | 460.14012 | 364.532000 |
| M790T304 | 789.51051 | 304.001500 |
| M523T427 | 523.05097 | 426.516000 |
| M361T580 | 360.87479 | 580.218000 |
| M1035T213 | 1035.01525 | 212.891000 |
| M402T370 | 402.03703 | 369.796000 |
| M996T600 | 995.69232 | 600.325000 |
| M359T289 | 359.02724 | 289.392000 |
| M322T295 | 322.07696 | 295.405000 |
| M359T584 | 358.85081 | 583.522000 |
| M420T396 | 420.09773 | 395.730000 |
| M169T336 | 169.06196 | 335.869500 |
| M643T464 | 642.70433 | 463.840000 |
| M415T401 | 414.94032 | 400.514000 |
| M1034T365 | 1034.18563 | 364.826000 |
| M308T335 | 308.10027 | 335.256000 |
| M122T293 | 121.94410 | 292.933000 |
| M281T515 | 280.86904 | 514.601000 |
| M371T294 | 371.01837 | 294.343000 |
| M378T488 | 378.12569 | 487.968500 |
| M289T417 | 288.99760 | 417.303500 |
| M915T467 | 914.94073 | 466.829000 |
| M313T470 | 312.87401 | 470.261000 |
| M446T400 | 446.07751 | 399.983500 |
| M1172T416 | 1172.24506 | 416.379000 |
| M304T366 | 304.05261 | 366.148500 |
| M457T366 | 457.01157 | 365.887000 |
| M554T213 | 553.99129 | 212.786000 |
| M143T276 | 142.94422 | 275.830000 |
| M405T433 | 405.10160 | 432.704000 |
| M760T17 | 759.50044 | 17.096550 |
| M594T395 | 594.12220 | 395.437000 |
| M425T500 | 424.86744 | 500.073000 |
| M285T364 | 285.07057 | 363.820000 |
| M313T360 | 313.03984 | 359.883000 |
| M766T335 | 765.98680 | 334.686500 |
| M377T543 | 376.86169 | 543.457000 |
| M369T484 | 369.11295 | 484.433500 |
| M542T365 | 542.07140 | 364.866000 |
| M761T302 | 760.74410 | 301.936000 |
| M369T472 | 369.11290 | 472.231500 |
| M633T228 | 633.09797 | 227.715500 |
| M880T438 | 880.43983 | 437.796000 |
| M473T474 | 473.11069 | 473.526000 |
| M205T297 | 204.93639 | 297.377000 |
| M525T302 | 524.96740 | 302.418500 |
| M593T461 | 593.13340 | 460.562000 |
| M126T393 | 125.92890 | 392.933000 |
| M185T283 | 184.94432 | 282.754000 |
| M317T294 | 317.00330 | 294.101000 |
| M243T447 | 243.09879 | 447.003500 |
| M299T592 | 298.93174 | 592.024500 |
| M1032T213 | 1032.35177 | 212.891000 |
| M389T285 | 389.16065 | 285.387000 |
| M328T356 | 328.03279 | 355.567000 |
| M341T596 | 340.91628 | 595.934000 |
| M481T212 | 480.65768 | 212.433000 |
| M692T424_1 | 692.00234 | 423.553500 |
| M838T212_3 | 838.33913 | 211.955000 |
| M287T511 | 287.11008 | 511.426000 |
| M763T365 | 763.13633 | 364.976500 |
| M435T425 | 435.03052 | 425.454000 |
| M427T600 | 427.01889 | 599.644000 |
| M1013T465 | 1012.91042 | 465.052500 |
| M759T334 | 759.49989 | 334.289000 |
| M373T284 | 373.18292 | 284.425500 |
| M510T392 | 510.19192 | 391.728000 |
| M819T365 | 818.53687 | 365.466000 |
| M496T428 | 496.04044 | 428.124500 |
| M495T250 | 495.22670 | 250.071500 |
| M189T504 | 189.41137 | 503.902000 |
| M274T460 | 274.01185 | 459.894000 |
| M493T480 | 492.94642 | 479.604500 |
| M432T361 | 432.09806 | 361.282000 |
| M273T278 | 272.88348 | 277.546500 |
| M377T341 | 377.05557 | 340.632000 |
| M529T593 | 529.03820 | 593.327500 |
| M463T253 | 463.16240 | 253.169000 |
| M803T212_1 | 802.67124 | 211.899000 |
| M388T354 | 388.13619 | 354.353500 |
| M295T338 | 295.11488 | 337.738000 |
| M295T340 | 295.05245 | 339.892000 |
| M279T412_1 | 278.96317 | 412.246000 |
| M274T453 | 273.96170 | 452.830000 |
| M353T361 | 353.15669 | 361.016500 |
| M281T585 | 280.92115 | 584.857500 |
| M469T248 | 469.11632 | 248.104500 |
| M317T210 | 317.21236 | 210.069000 |
| M274T386_2 | 274.09109 | 385.929000 |
| M468T372 | 468.04521 | 371.939500 |
| M484T387 | 484.16801 | 386.613000 |
| M377T294_1 | 377.02406 | 293.791000 |
| M437T213 | 437.48652 | 212.896500 |
| M273T286 | 273.10092 | 285.780000 |
| M490T283 | 490.25574 | 282.904000 |
| M385T502 | 384.80405 | 501.844000 |
| M876T211 | 875.76214 | 210.656000 |
| M1049T211_1 | 1048.68855 | 210.973000 |
| M695T214 | 695.00017 | 213.747500 |
| M403T582 | 402.90415 | 581.606000 |
| M308T428 | 307.98974 | 427.715000 |
| M199T413 | 198.95704 | 412.589000 |
| M678T213_1 | 677.66477 | 212.815000 |
| M363T447 | 363.17632 | 447.372500 |
| M260T340 | 260.04428 | 339.683500 |
| M422T404 | 422.17825 | 404.496000 |
| M799T343 | 799.00982 | 343.021000 |
| M543T435 | 542.98152 | 435.411000 |
| M308T239 | 307.92544 | 238.755000 |
| M312T437 | 312.11469 | 437.067500 |
| M353T283 | 353.07793 | 282.534000 |
| M871T213 | 871.34532 | 212.587500 |
| M775T371 | 775.07089 | 370.856000 |
| M381T388 | 381.10059 | 388.254000 |
| M626T234 | 626.36811 | 234.434500 |
| M350T461 | 350.10594 | 461.173000 |
| M381T583 | 380.83278 | 582.843000 |
| M1019T213 | 1018.67867 | 212.597000 |
| M242T479 | 241.99960 | 478.928500 |
| M247T283 | 247.16636 | 283.340000 |
| M665T464 | 664.68645 | 464.431000 |
| M501T424_1 | 500.98556 | 423.817000 |
| M1076T211_1 | 1075.52321 | 211.329500 |
| M751T459 | 750.86865 | 458.602000 |
| M311T461 | 311.00686 | 460.872000 |
| M619T359 | 619.07010 | 359.482000 |
| M919T335 | 918.98039 | 334.694000 |
| M857T335 | 856.94628 | 334.823500 |
| M199T452 | 198.90556 | 451.504500 |
| M175T615 | 174.88717 | 615.191000 |
| M410T241 | 409.97258 | 241.315500 |
| M503T490 | 503.16203 | 489.727000 |
| M194T351_3 | 194.06659 | 351.026000 |
| M253T472 | 253.05658 | 472.259500 |
| M787T213 | 787.00065 | 213.070000 |
| M283T423 | 282.99585 | 423.201000 |
| M1011T365 | 1011.21867 | 364.759000 |
| M735T428 | 735.36733 | 427.824000 |
| M984T214 | 984.00855 | 213.759000 |
| M546T434 | 546.01132 | 434.377000 |
| M853T302 | 852.96131 | 302.243000 |
| M194T351_2 | 194.05474 | 350.960000 |
| M929T213 | 929.00849 | 212.811000 |
| M747T490 | 747.21784 | 489.734000 |
| M628T335 | 627.97615 | 334.727500 |
| M904T212 | 904.26071 | 212.141000 |
| M302T356 | 302.12228 | 356.335000 |
| M386T371 | 386.04213 | 370.949500 |
| M722T335 | 721.95993 | 335.307000 |
| M495T468 | 494.92364 | 467.600000 |
| M1033T212 | 1033.01683 | 212.463000 |
| M327T472 | 327.17634 | 472.273000 |
| M182T214 | 182.08233 | 214.394000 |
| M410T393 | 410.12725 | 393.271000 |
| M947T213_2 | 947.25789 | 213.392000 |
| M447T393 | 447.15303 | 392.856500 |
| M586T446 | 586.29578 | 446.216500 |
| M533T469 | 533.20442 | 468.687000 |
| M447T449 | 447.03494 | 448.915000 |
| M589T213_1 | 588.66086 | 213.297500 |
| M265T472 | 265.05706 | 472.210000 |
| M564T423 | 564.01397 | 422.627000 |
| M312T432 | 312.00162 | 431.675000 |
| M467T502 | 466.80707 | 502.130000 |
| M154T448 | 154.01225 | 448.130000 |
| M429T213 | 428.98540 | 212.810000 |
| M519T496 | 519.20578 | 495.967000 |
| M153T252 | 153.10337 | 252.275000 |
| M437T293 | 437.04409 | 293.423000 |
| M337T257 | 336.98520 | 256.950000 |
| M461T293 | 461.08843 | 293.457500 |
| M239T614 | 238.91282 | 614.135500 |
| M216T294 | 216.08776 | 294.068000 |
| M751T371_2 | 751.11037 | 370.764500 |
| M651T344 | 650.97994 | 344.353000 |
| M386T399 | 386.05615 | 399.135000 |
| M240T289 | 240.06265 | 288.577000 |
| M962T598 | 961.61177 | 597.860000 |
| M342T371_1 | 341.99487 | 370.799000 |
| M545T448 | 545.01548 | 448.049500 |
| M617T303 | 616.86532 | 302.628000 |
| M360T357 | 360.12773 | 356.811000 |
| M388T295 | 388.03576 | 294.897000 |
| M659T214 | 658.66615 | 213.537000 |
| M761T460 | 761.22704 | 460.166500 |
| M141T447 | 141.06700 | 446.829000 |
| M419T534 | 418.88061 | 534.174000 |
| M645T370 | 645.06235 | 370.168500 |
| M645T364 | 645.15098 | 363.631000 |
| M327T445 | 327.03314 | 445.113500 |
| M447T213 | 446.65328 | 213.444500 |
| M500T356 | 500.08491 | 355.732500 |
| M805T463 | 805.19158 | 462.900000 |
| M301T446 | 301.16325 | 445.923500 |
| M374T470 | 374.07864 | 470.359000 |
| M431T360 | 431.01928 | 359.935000 |
| M676T213 | 675.66871 | 213.067000 |
| M303T213 | 302.98106 | 213.379000 |
| M587T212 | 587.32919 | 212.258000 |
| M483T212 | 482.65464 | 212.495000 |
| M551T212_1 | 550.66278 | 212.498000 |
| M578T366 | 578.38536 | 366.159000 |
| M287T405 | 287.10405 | 404.926500 |
| M469T257 | 469.19230 | 256.593000 |
| M410T356 | 410.03574 | 355.683000 |
| M575T501 | 575.02675 | 500.652000 |
| M293T367 | 293.01635 | 367.372000 |
| M339T303 | 339.23291 | 302.872000 |
| M361T293 | 361.12895 | 293.492000 |
| M545T593 | 545.01523 | 593.332000 |
| M451T485 | 451.11611 | 485.151000 |
| M623T435 | 622.98544 | 434.818000 |
| M533T344 | 532.98324 | 343.752500 |

Table S4 460 metabolites in negative mode in urine samples

| **ID** | **m/z** | **rt(s)** |
| --- | --- | --- |
| M261T597 | 260.91041 | 596.577000 |
| M267T370 | 266.90904 | 369.675000 |
| M977T211 | 977.35121 | 211.385000 |
| M874T210 | 874.00783 | 210.087000 |
| M728T349 | 728.03834 | 348.995000 |
| M366T397 | 366.10869 | 396.553500 |
| M272T465 | 271.86486 | 465.072000 |
| M227T210 | 227.10389 | 209.979500 |
| M78T477_2 | 77.59106 | 476.910000 |
| M298T405 | 298.06995 | 405.439000 |
| M334T228 | 334.11934 | 227.551000 |
| M707T459 | 707.06769 | 459.254000 |
| M1065T212 | 1064.76838 | 212.092000 |
| M259T596 | 258.91344 | 596.469000 |
| M323T314 | 323.07710 | 313.655000 |
| M199T345 | 198.97572 | 345.390000 |
| M463T593 | 463.01226 | 593.317000 |
| M321T400 | 321.03185 | 400.213000 |
| M514T361 | 514.10131 | 361.229500 |
| M165T452 | 164.89558 | 452.196000 |
| M401T392 | 401.20428 | 391.603500 |
| M317T364 | 317.04956 | 364.478500 |
| M401T480 | 401.06943 | 480.056500 |
| M689T338 | 689.07367 | 337.738000 |
| M1093T213_1 | 1092.76754 | 212.604000 |
| M413T403 | 413.00619 | 402.521500 |
| M1172T211 | 1172.27309 | 211.366000 |
| M79T478 | 78.76071 | 477.648000 |
| M620T300 | 619.97280 | 299.672500 |
| M220T295_1 | 220.04686 | 294.589500 |
| M173T446 | 173.09326 | 446.371000 |
| M199T292 | 199.05865 | 292.296000 |
| M1106T213_1 | 1105.76875 | 212.831500 |
| M308T412 | 308.03668 | 411.570000 |
| M359T425 | 359.15734 | 425.074000 |
| M767T394 | 767.15587 | 393.519000 |
| M439T250 | 439.27352 | 249.966000 |
| M331T447 | 331.01392 | 447.085000 |
| M383T460 | 382.89737 | 460.303500 |
| M1071T423 | 1071.18213 | 422.619500 |
| M281T521 | 281.36100 | 521.356000 |
| M814T389 | 814.46825 | 388.720000 |
| M345T601 | 345.01554 | 601.010500 |
| M607T300 | 606.86376 | 300.071000 |
| M519T335 | 519.34823 | 334.973000 |
| M444T299 | 443.97268 | 299.115000 |
| M544T434 | 544.01429 | 434.409000 |
| M883T457 | 883.01368 | 456.730000 |
| M94T299 | 93.87054 | 299.471500 |
| M261T567 | 260.89242 | 567.209000 |
| M409T416 | 409.06012 | 415.591000 |
| M722T393 | 722.27938 | 392.772000 |
| M453T460 | 453.00884 | 460.455000 |
| M561T467 | 560.95251 | 467.077500 |
| M421T544 | 420.82637 | 544.216000 |
| M348T384 | 348.07243 | 383.712000 |
| M1003T597 | 1002.62541 | 597.220000 |
| M1092T213 | 1092.26973 | 212.838500 |
| M518T211 | 517.98966 | 210.985000 |
| M72T366 | 72.00912 | 365.938000 |
| M235T545 | 234.91384 | 545.329500 |
| M345T406 | 345.12929 | 405.905000 |
| M409T212_3 | 409.32050 | 212.319000 |
| M291T356 | 291.16752 | 355.508000 |
| M398T224 | 398.10454 | 223.515000 |
| M952T365 | 952.18252 | 365.189000 |
| M552T211 | 551.99432 | 211.151000 |
| M338T310 | 338.05589 | 309.909500 |
| M980T492_1 | 979.92076 | 492.474000 |
| M277T370 | 277.05101 | 370.104000 |
| M605T237 | 605.15178 | 237.203000 |
| M424T210 | 424.28334 | 210.366000 |
| M483T383 | 483.09292 | 382.734500 |
| M298T227 | 298.07020 | 226.653000 |
| M258T380 | 258.18243 | 380.486000 |
| M338T404 | 338.06271 | 404.229000 |
| M1169T378 | 1169.33980 | 378.098500 |
| M926T214 | 925.67900 | 213.573500 |
| M254T356_1 | 254.06475 | 356.475500 |
| M393T393 | 393.14802 | 393.307000 |
| M331T614 | 330.91911 | 613.941000 |
| M829T302 | 828.94201 | 302.099000 |
| M233T470 | 233.06021 | 470.355500 |
| M640T404 | 640.11702 | 404.377000 |
| M827T386 | 827.31788 | 385.931500 |
| M122T367 | 122.07243 | 366.560000 |
| M751T466 | 751.03117 | 465.695000 |
| M876T213 | 876.00608 | 213.428500 |
| M294T400 | 294.02075 | 400.201000 |
| M526T227 | 526.19866 | 226.901000 |
| M633T595_1 | 632.95250 | 595.269000 |
| M893T465 | 892.95953 | 465.107000 |
| M705T428 | 705.08032 | 427.852000 |
| M409T212_2 | 408.98769 | 212.099000 |
| M297T352 | 297.08200 | 351.582500 |
| M388T464 | 388.04240 | 463.504000 |
| M325T256 | 325.06542 | 256.060500 |
| M503T461 | 503.24497 | 461.155500 |
| M295T456 | 294.88709 | 456.017500 |
| M491T393 | 491.12533 | 392.809000 |
| M362T355 | 362.05575 | 354.829000 |
| M425T526 | 424.96982 | 525.559500 |
| M358T212 | 357.98139 | 212.144000 |
| M737T435 | 736.97689 | 435.156000 |
| M159T452 | 158.89323 | 452.231500 |
| M611T509 | 611.14509 | 509.105000 |
| M272T481 | 272.12505 | 480.884000 |
| M247T546 | 246.95020 | 546.407000 |
| M221T215 | 220.93154 | 214.569000 |
| M471T480 | 470.97119 | 480.048500 |
| M629T479 | 629.10851 | 479.059000 |
| M365T449 | 365.03159 | 448.899500 |
| M263T465 | 262.87806 | 464.984000 |
| M336T272 | 336.06056 | 271.910000 |
| M255T283 | 254.97681 | 282.599000 |
| M120T489 | 120.01252 | 489.052000 |
| M682T364 | 682.21081 | 364.407000 |
| M309T399 | 309.10951 | 398.753000 |
| M311T464 | 310.85507 | 463.815000 |
| M950T618 | 950.11582 | 617.934000 |
| M680T383 | 680.12113 | 382.840500 |
| M272T305 | 272.07534 | 304.995000 |
| M539T464 | 538.72581 | 464.463500 |
| M1179T300 | 1178.69470 | 299.677000 |
| M377T220 | 377.05334 | 220.346000 |
| M345T210 | 345.06507 | 210.234000 |
| M119T459 | 118.94217 | 459.185000 |
| M373T422 | 372.99618 | 422.431500 |
| M525T393 | 525.20180 | 392.776000 |
| M331T461 | 331.09399 | 461.179000 |
| M385T377 | 385.14638 | 376.843500 |
| M450T469 | 450.17294 | 469.090000 |
| M713T339 | 713.08548 | 338.884500 |
| M307T447 | 307.05257 | 447.022500 |
| M927T213 | 927.01135 | 213.026000 |
| M974T387 | 974.32907 | 386.886000 |
| M787T480_2 | 787.35530 | 479.935500 |
| M411T600 | 410.98750 | 599.776000 |
| M471T246 | 471.13027 | 246.460500 |
| M292T334 | 292.14027 | 334.130000 |
| M653T461 | 653.06259 | 461.193000 |
| M326T426 | 326.01721 | 426.033500 |
| M119T310 | 119.05031 | 310.378500 |
| M1049T422 | 1049.19757 | 422.316000 |
| M903T459 | 902.99771 | 458.597500 |
| M280T387 | 280.02061 | 387.297000 |
| M306T398 | 306.08303 | 398.433000 |
| M276T364 | 276.07032 | 364.366500 |
| M759T393 | 759.30657 | 392.509000 |
| M708T465 | 707.85655 | 465.079500 |
| M198T310 | 198.03832 | 309.855000 |
| M756T478 | 756.28830 | 477.800000 |
| M97T616 | 96.92212 | 615.924000 |
| M943T335 | 942.99851 | 334.500500 |
| M216T474 | 216.13538 | 474.215000 |
| M306T213 | 305.97687 | 213.452500 |
| M301T355 | 301.16584 | 354.783500 |
| M733T344 | 732.98304 | 343.522000 |
| M732T212 | 731.99946 | 211.823500 |
| M553T248 | 553.15620 | 248.082000 |
| M932T299 | 931.85887 | 298.735500 |
| M947T456 | 946.80225 | 456.022000 |
| M395T351 | 395.12747 | 350.624000 |
| M129T441 | 128.93224 | 440.900000 |
| M245T592 | 244.96119 | 592.021000 |
| M479T461 | 479.04230 | 460.539000 |
| M169T352_2 | 169.06191 | 351.978000 |
| M365T248 | 365.08614 | 248.474000 |
| M202T383 | 202.05291 | 383.011000 |
| M220T432 | 220.06500 | 431.999000 |
| M989T445 | 989.45405 | 444.545000 |
| M469T284 | 469.12309 | 283.636000 |
| M593T231 | 593.44280 | 230.576000 |
| M425T589 | 424.96972 | 588.855500 |
| M439T300 | 438.90463 | 299.628000 |
| M201T437 | 201.13578 | 437.162500 |
| M1079T212 | 1079.26821 | 212.083000 |
| M332T395 | 332.10810 | 394.831000 |
| M1013T211 | 1013.26255 | 211.156500 |
| M1070T387 | 1070.40578 | 386.625000 |
| M430T420 | 430.19460 | 420.094500 |
| M815T341 | 814.98608 | 340.989000 |
| M247T344 | 246.98137 | 344.322000 |
| M733T299 | 732.83689 | 299.462000 |
| M850T477 | 850.23262 | 476.863500 |
| M615T343 | 614.98639 | 342.530000 |
| M831T300 | 830.81363 | 299.649000 |
| M298T387 | 298.03131 | 386.920000 |
| M386T438 | 385.95696 | 437.779500 |
| M365T423 | 365.05767 | 423.341500 |
| M284T294_2 | 283.98556 | 294.212500 |
| M459T340 | 459.05876 | 340.351000 |
| M898T334 | 897.94755 | 334.154000 |
| M162T452 | 161.89416 | 452.184000 |
| M132T351 | 132.03028 | 351.164500 |
| M517T213 | 516.99141 | 213.135000 |
| M776T383 | 776.23951 | 382.749000 |
| M1109T300 | 1108.73682 | 299.717000 |
| M310T464 | 309.85615 | 463.833500 |
| M171T215_2 | 171.07762 | 214.994000 |
| M230T490 | 230.06722 | 489.731000 |
| M1066T212_2 | 1066.26672 | 212.235500 |
| M144T217 | 143.89227 | 216.753000 |
| M391T435 | 390.95287 | 435.126000 |
| M481T464 | 480.79475 | 463.768000 |
| M519T303 | 518.88874 | 302.874000 |
| M111T219 | 110.89910 | 219.015000 |
| M679T592 | 679.39276 | 592.043000 |
| M807T464 | 806.61670 | 464.435000 |
| M447T591 | 447.09360 | 591.376000 |
| M933T226 | 933.47220 | 226.073500 |
| M859T301 | 858.72098 | 301.168000 |
| M249T283 | 249.09149 | 283.351000 |
| M275T225 | 275.02035 | 224.889000 |
| M417T451 | 417.16060 | 450.677000 |
| M244T462 | 244.13040 | 461.823000 |
| M183T283 | 182.94722 | 282.962500 |
| M307T464 | 306.85703 | 464.119000 |
| M168T446 | 168.11433 | 446.371000 |
| M476T352 | 476.14657 | 351.635000 |
| M848T383 | 848.17226 | 382.628000 |
| M193T370 | 192.91143 | 369.742500 |
| M601T480 | 601.10320 | 479.514500 |
| M900T209 | 899.52773 | 209.005000 |
| M324T247 | 324.00127 | 246.846000 |
| M317T436_1 | 316.89016 | 435.899000 |
| M246T305 | 246.08449 | 304.979000 |
| M299T399 | 299.02636 | 398.775500 |
| M172T435 | 172.06164 | 434.913000 |
| M834T213 | 834.01247 | 212.852000 |
| M322T472 | 322.14976 | 472.268000 |
| M258T367 | 257.96823 | 366.671500 |
| M82T332 | 82.11174 | 332.383000 |
| M873T417_4 | 873.43657 | 416.517000 |
| M400T431 | 400.02141 | 431.453500 |
| M1046T247 | 1046.47549 | 247.343500 |
| M435T435 | 434.93474 | 435.161000 |
| M356T312 | 356.06363 | 311.573000 |
| M475T479 | 475.13601 | 478.888500 |
| M873T209 | 873.34208 | 208.781000 |
| M77T232 | 76.97006 | 231.593000 |
| M161T335 | 161.06622 | 335.151000 |
| M128T220_1 | 127.89737 | 220.149000 |
| M343T568 | 342.89545 | 567.518000 |
| M315T345 | 314.92953 | 344.582000 |
| M687T340 | 687.00756 | 339.503500 |
| M690T383 | 690.19158 | 382.808000 |
| M517T211 | 516.65867 | 211.129000 |
| M277T352 | 277.05911 | 351.698500 |
| M666T590 | 666.06016 | 589.760000 |
| M319T583 | 318.87683 | 582.807000 |
| M423T471 | 423.19515 | 470.993000 |
| M296T453_1 | 295.94362 | 452.809000 |
| M323T303 | 322.93457 | 303.256000 |
| M464T396 | 464.04788 | 396.096500 |
| M293T618 | 292.89153 | 618.482000 |
| M552T372 | 552.02899 | 372.387000 |
| M380T294 | 380.08711 | 293.863000 |
| M325T471 | 325.16172 | 471.245500 |
| M915T302 | 914.77475 | 301.755500 |
| M421T303 | 420.91150 | 303.110000 |
| M373T222 | 373.10177 | 222.400000 |
| M363T407 | 363.14632 | 407.261000 |
| M591T344_2 | 591.02514 | 344.304000 |
| M328T461 | 328.02129 | 461.216000 |
| M357T433 | 357.08283 | 432.711000 |
| M381T449 | 381.00942 | 449.470000 |
| M583T466 | 582.68805 | 466.401500 |
| M534T396 | 534.10158 | 395.667000 |
| M353T459 | 352.82322 | 458.630500 |
| M1017T212 | 1017.34750 | 212.462000 |
| M270T354 | 270.10152 | 354.262500 |
| M181T374 | 181.07218 | 374.125000 |
| M1118T212_2 | 1118.27187 | 212.003500 |
| M763T467 | 762.81238 | 467.485500 |
| M193T276 | 192.91146 | 276.422000 |
| M435T396 | 435.00068 | 396.174000 |
| M369T422_1 | 369.04290 | 421.723500 |
| M385T412 | 385.01069 | 412.459500 |
| M566T434 | 565.99633 | 434.384500 |
| M284T333 | 284.03061 | 333.470000 |
| M509T480 | 509.00167 | 480.106500 |
| M393T435 | 392.94996 | 435.247000 |
| M718T300 | 717.95010 | 299.660000 |
| M349T355 | 349.07185 | 354.615000 |
| M225T287 | 224.98974 | 286.543000 |
| M573T249 | 573.15286 | 248.746000 |
| M254T438 | 253.94944 | 437.642500 |
| M252T436 | 251.95259 | 436.172000 |
| M948T423 | 948.06961 | 423.149500 |
| M358T370 | 358.04321 | 369.959500 |
| M542T210 | 542.28892 | 209.981000 |
| M113T311 | 113.06083 | 310.822500 |
| M534T292 | 534.15200 | 292.049000 |
| M943T598 | 942.64118 | 597.777000 |
| M802T213 | 802.00495 | 212.534000 |
| M302T393 | 302.11645 | 392.823500 |
| M373T461 | 372.99445 | 460.550000 |
| M779T300 | 778.86188 | 300.267000 |
| M280T465 | 279.84730 | 465.109000 |
| M731T212_2 | 731.33379 | 212.258000 |
| M723T378 | 723.14675 | 378.482500 |
| M393T285 | 393.08564 | 284.637000 |
| M356T422 | 356.10738 | 422.316000 |
| M389T452 | 389.15441 | 452.195000 |
| M434T404 | 434.13953 | 403.861000 |
| M541T435 | 540.98236 | 435.159000 |
| M339T429 | 339.01199 | 429.481000 |
| M621T435 | 620.98830 | 435.399500 |
| M518T467_2 | 518.23139 | 466.585000 |
| M96T207 | 95.61609 | 207.251000 |
| M347T416 | 346.99297 | 415.554000 |
| M623T591 | 623.44694 | 591.417500 |
| M259T298 | 258.96401 | 297.861500 |
| M229T441 | 229.04722 | 440.756000 |
| M791T394 | 791.20054 | 394.356000 |
| M321T387 | 321.04710 | 386.903500 |
| M1180T602 | 1179.78119 | 602.290500 |
| M981T464 | 980.72825 | 464.435500 |
| M307T351 | 307.11395 | 350.969000 |
| M244T311 | 244.05921 | 311.106000 |
| M549T274 | 549.35878 | 273.654000 |
| M81T441 | 80.96522 | 440.857000 |
| M362T443 | 362.02348 | 442.867000 |
| M255T618 | 254.90675 | 617.746000 |
| M317T470 | 316.84763 | 469.733000 |
| M768T213 | 768.00095 | 212.676000 |
| M889T213 | 889.01269 | 212.660000 |
| M719T302 | 718.82073 | 302.063000 |
| M303T438 | 303.00317 | 437.796000 |
| M172T238 | 171.88879 | 237.729000 |
| M558T365 | 558.12065 | 364.878500 |
| M266T273 | 265.94698 | 273.031000 |
| M214T433 | 214.13109 | 433.131000 |
| M446T212 | 445.98746 | 211.706000 |
| M345T400 | 345.06876 | 399.751500 |
| M551T597 | 550.94930 | 596.556000 |
| M339T301 | 338.96880 | 300.573000 |
| M207T220 | 206.82246 | 220.149000 |
| M891T378 | 891.19797 | 377.817500 |
| M132T283 | 132.03030 | 283.068000 |
| M285T479_1 | 285.03966 | 478.949500 |
| M278T395 | 278.07350 | 394.577000 |
| M365T446 | 364.96107 | 445.700500 |
| M729T462 | 729.04804 | 461.842000 |
| M245T221 | 244.80948 | 220.729000 |
| M274T285 | 274.09644 | 284.916000 |
| M619T303 | 618.84421 | 302.612000 |
| M283T542 | 283.35816 | 542.056500 |
| M717T344 | 717.00647 | 343.573000 |
| M717T338 | 716.95340 | 338.160000 |
| M624T236 | 624.35206 | 236.156000 |
| M107T213 | 106.92283 | 212.802000 |
| M299T473 | 299.17018 | 473.027000 |
| M245T457 | 244.85250 | 456.684000 |
| M1063T212 | 1063.27073 | 211.686000 |
| M1186T213 | 1185.52416 | 212.540500 |
| M251T351 | 251.09721 | 351.071500 |
| M443T387 | 443.14134 | 386.717500 |
| M598T239 | 598.33672 | 238.619000 |
| M422T426 | 422.11600 | 425.674500 |
| M380T504 | 379.83006 | 503.864500 |
| M419T426_1 | 419.00078 | 425.686000 |
| M476T250 | 476.18295 | 250.041000 |
| M1066T212_1 | 1065.76617 | 212.462000 |
| M411T212_2 | 410.98493 | 211.896000 |
| M443T310 | 443.06047 | 309.736000 |
| M390T461 | 390.04012 | 460.550000 |
| M582T478_2 | 582.28466 | 477.555500 |
| M108T393 | 108.05674 | 393.006000 |
| M851T335 | 851.01400 | 335.196000 |
| M621T464 | 620.72258 | 463.732000 |
| M778T478 | 778.26969 | 477.586000 |
| M745T457 | 744.85860 | 456.689000 |
| M761T471 | 761.36964 | 470.975000 |
| M377T248 | 377.00535 | 247.548500 |
| M433T435 | 432.93782 | 435.124500 |
| M203T373 | 203.10381 | 373.281000 |
| M722T333 | 722.46366 | 333.179000 |
| M920T211 | 919.50688 | 211.229000 |
| M309T431_2 | 309.08179 | 430.789500 |
| M1166T417 | 1165.58570 | 416.661000 |
| M903T338 | 903.05715 | 338.255000 |
| M258T415 | 258.04735 | 415.405000 |
| M493T598 | 492.99067 | 598.028000 |
| M259T413 | 258.96018 | 412.567000 |
| M309T359 | 309.10702 | 358.506000 |
| M78T593_2 | 77.83191 | 593.319000 |
| M330T438 | 329.99065 | 437.856000 |
| M445T354 | 445.07931 | 354.208000 |
| M519T415 | 519.04031 | 415.140000 |
| M167T398 | 167.05754 | 398.058000 |
| M513T301 | 512.93378 | 300.528000 |
| M404T335 | 404.26783 | 335.453000 |
| M473T444_2 | 473.24452 | 444.424000 |
| M504T371 | 504.02219 | 371.083000 |
| M156T215 | 156.03042 | 215.090000 |
| M171T215_1 | 171.04121 | 214.900000 |
| M116T378 | 115.92068 | 378.413000 |
| M304T212 | 303.97977 | 212.072500 |
| M580T355 | 580.05930 | 354.981000 |
| M882T457 | 882.01838 | 456.679000 |
| M512T466 | 511.84268 | 466.402000 |
| M228T466 | 228.05223 | 465.787000 |
| M263T461 | 263.16142 | 461.194000 |
| M287T400 | 286.95513 | 400.279000 |
| M810T367 | 810.14737 | 367.471500 |
| M621T469 | 620.88500 | 468.797000 |
| M142T215 | 141.98040 | 215.479000 |
| M987T365 | 987.25786 | 364.603000 |
| M394T351 | 394.14353 | 351.060000 |
| M252T460 | 251.97976 | 459.828000 |
| M221T482 | 221.06051 | 481.521000 |
| M897T338 | 896.98932 | 337.946000 |
| M851T351 | 851.35541 | 350.585000 |
| M244T447 | 244.08342 | 446.951500 |
| M358T422 | 358.01317 | 422.422500 |
| M320T392 | 320.15773 | 391.602500 |
| M781T333 | 781.48273 | 333.225000 |
| M817T302 | 816.79748 | 301.980000 |
| M323T352 | 323.09521 | 351.928000 |
| M315T463 | 315.16756 | 463.139500 |
| M302T367 | 302.02034 | 366.818000 |
| M240T209 | 240.09901 | 208.608000 |
| M877T338 | 877.04115 | 337.952500 |
| M129T370 | 129.09211 | 369.904000 |
| M115T388 | 115.02309 | 388.195000 |
| M463T450 | 463.01246 | 449.583000 |
| M169T422 | 169.06198 | 422.288000 |
| M457T404 | 457.15543 | 403.832500 |
| M350T430 | 350.07045 | 430.447000 |
| M205T350 | 205.06191 | 350.204500 |
| M753T423 | 753.03516 | 422.573500 |
| M241T221 | 240.78967 | 220.679500 |
| M707T461 | 707.22409 | 460.793000 |
| M204T338 | 204.07796 | 337.790000 |
| M1119T214 | 1118.77045 | 213.603000 |
| M441T591 | 440.94710 | 591.094000 |
| M567T250 | 567.13702 | 249.661000 |
| M929T300 | 928.79087 | 300.448000 |
| M730T466 | 729.83795 | 465.766000 |
| M351T587 | 350.86056 | 587.348000 |
| M400T350 | 400.11192 | 350.410000 |
| M834T369 | 834.10798 | 369.242500 |
| M561T465 | 560.70788 | 465.117000 |
| M657T479 | 656.85736 | 479.489500 |
| M469T223 | 469.14151 | 223.378500 |
| M772T437 | 771.94745 | 437.251000 |
| M269T305_1 | 269.00489 | 304.783500 |
| M597T481 | 597.02153 | 481.437500 |
| M411T300 | 410.90942 | 299.644000 |
| M535T394 | 535.17269 | 393.612000 |
| M236T449 | 236.06543 | 449.347000 |
| M447T503 | 447.06298 | 503.112000 |
| M691T429 | 691.06371 | 428.589000 |
| M349T227 | 349.09477 | 227.353500 |
| M262T442 | 262.09332 | 441.621000 |
| M647T469 | 647.03798 | 468.876000 |
| M84T494_2 | 83.92584 | 493.716000 |
| M727T428 | 727.06401 | 427.902000 |

Table S5 87 genes in up mode between the CON and MOD groups

| **rownames(res)** | **log_2_FoldChange** | **-log_10_FDA** |
| --- | --- | --- |
| ENSMUSG00000025986 | 1.569020389 | 2.316797318 |
| ENSMUSG00000050534 | 1.782015088 | 1.54014178 |
| ENSMUSG00000067780 | 1.159293459 | 1.524598256 |
| ENSMUSG00000025927 | 1.39637921 | 1.473905092 |
| ENSMUSG00000042215 | 1.141970086 | 1.958168771 |
| ENSMUSG00000026023 | 1.257592473 | 1.780978094 |
| ENSMUSG00000053024 | 2.82273457 | 3.328339868 |
| ENSMUSG00000037434 | 1.570395521 | 2.656164726 |
| ENSMUSG00000058248 | 1.47975575 | 1.836429046 |
| ENSMUSG00000102700 | 2.041782129 | 1.570091347 |
| ENSMUSG00000025991 | 1.601687517 | 2.182734064 |
| ENSMUSG00000097083 | 2.438177174 | 2.191791914 |
| ENSMUSG00000062588 | 1.681596698 | 1.845676928 |
| ENSMUSG00000049598 | 2.414468351 | 3.132501657 |
| ENSMUSG00000032852 | 1.984477662 | 1.52168191 |
| ENSMUSG00000078899 | 4.223615492 | 2.283545984 |
| ENSMUSG00000009614 | 1.257865604 | 1.421541275 |
| ENSMUSG00000027577 | 2.905703843 | 2.098757419 |
| ENSMUSG00000027070 | 1.91240248 | 1.395311583 |
| ENSMUSG00000074652 | 1.945801012 | 1.470590967 |
| ENSMUSG00000027015 | 1.454758216 | 2.127783134 |
| ENSMUSG00000003418 | 2.068469965 | 3.042606535 |
| ENSMUSG00000041911 | 1.75534007 | 1.657064117 |
| ENSMUSG00000035183 | 2.116417136 | 2.305362661 |
| ENSMUSG00000076441 | 2.070383983 | 2.572276049 |
| ENSMUSG00000017861 | 1.489357949 | 1.475246443 |
| ENSMUSG00000032841 | 2.04087396 | 2.474482775 |
| ENSMUSG00000027401 | 1.255401418 | 1.340990594 |
| ENSMUSG00000037727 | 3.190122957 | 2.224109923 |
| ENSMUSG00000086214 | 3.789381024 | 4.029490683 |
| ENSMUSG00000050447 | 1.832945579 | 2.057294505 |
| ENSMUSG00000027403 | 1.679950568 | 1.528871143 |
| ENSMUSG00000025333 | 2.444033965 | 1.99386239 |
| ENSMUSG00000087644 | 1.792219122 | 1.963138165 |
| ENSMUSG00000071719 | 1.847349517 | 1.45529801 |
| ENSMUSG00000085715 | 4.366520953 | 2.316797318 |
| ENSMUSG00000028003 | 1.304962057 | 1.326214237 |
| ENSMUSG00000095836 | 2.141328609 | 2.348746205 |
| ENSMUSG00000104109 | 3.801708394 | 2.931992138 |
| ENSMUSG00000033222 | 1.149026412 | 1.54014178 |
| ENSMUSG00000074206 | 2.683622654 | 4.630502298 |
| ENSMUSG00000098008 | 6.507845899 | 3.080172362 |
| ENSMUSG00000077148 | 2.930060683 | 1.470590967 |
| ENSMUSG00000028186 | 2.195647418 | 1.638333572 |
| ENSMUSG00000001021 | 1.858368051 | 2.246172613 |
| ENSMUSG00000085933 | 2.278539132 | 2.716712342 |
| ENSMUSG00000028393 | 1.000259668 | 1.691896787 |
| ENSMUSG00000039492 | 1.471799636 | 2.098757419 |
| ENSMUSG00000037348 | 1.896480519 | 1.389593429 |
| ENSMUSG00000028487 | 1.839528437 | 2.981940426 |
| ENSMUSG00000078487 | 2.723509045 | 2.572276049 |
| ENSMUSG00000028634 | 1.057103843 | 1.434407158 |
| ENSMUSG00000070803 | 1.723383047 | 1.36935543 |
| ENSMUSG00000028654 | 1.644420138 | 2.057294505 |
| ENSMUSG00000006219 | 1.151930752 | 1.872981575 |
| ENSMUSG00000042489 | 1.386253205 | 1.451577027 |
| ENSMUSG00000078735 | 2.57499326 | 2.053163036 |
| ENSMUSG00000087383 | 1.388294868 | 1.71376609 |
| ENSMUSG00000070990 | 1.627431448 | 1.65993911 |
| ENSMUSG00000038656 | 4.008514675 | 1.582076595 |
| ENSMUSG00000041313 | 1.239155987 | 1.639601605 |
| ENSMUSG00000066975 | 2.019557756 | 1.941687857 |
| ENSMUSG00000105059 | 2.199010728 | 1.943655172 |
| ENSMUSG00000081683 | 1.73964456 | 2.145707895 |
| ENSMUSG00000054256 | 1.755563837 | 2.70477007 |
| ENSMUSG00000055725 | 1.768719576 | 1.653939069 |
| ENSMUSG00000029337 | 2.60207802 | 2.165923681 |
| ENSMUSG00000013629 | 1.503469062 | 2.201960932 |
| ENSMUSG00000036565 | 1.306490142 | 1.333464838 |
| ENSMUSG00000029816 | 1.728889353 | 2.576766762 |
| ENSMUSG00000111640 | 1.696962491 | 3.832124623 |
| ENSMUSG00000131482 | 2.067350943 | 5.442347015 |
| ENSMUSG00000159399 | 1.977715429 | 4.950857066 |
| ENSMUSG00000048776 | 2.294195649 | 2.527349439 |
| ENSMUSG00000160883 | 1.643476933 | 2.894659079 |
| ENSMUSG00000156515 | 1.800106208 | 4.236449901 |
| ENSMUSG00000105220 | 2.020771807 | 4.397205168 |
| ENSMUSG00000107502 | 1.775377917 | 1.963138165 |
| ENSMUSG00000004633 | 1.416303888 | 2.76957105 |
| ENSMUSG00000039904 | 1.830365081 | 2.316797318 |
| ENSMUSG00000107653 | 2.537727149 | 1.964005387 |
| ENSMUSG00000040797 | 2.449234011 | 1.657638214 |
| ENSMUSG00000070348 | 1.258521202 | 1.98895389 |
| ENSMUSG00000030523 | 2.756113523 | 2.284124498 |
| ENSMUSG00000108770 | 3.332678177 | 2.542898391 |
| ENSMUSG00000004651 | 1.74668509 | 1.324564126 |
| ENSMUSG00000030616 | 1.472938785 | 2.003232002 |

Table S6 323 genes in down mode between the CON and MOD groups

| **rownames(res)** | **log_2_FoldChange** | **-log_10_FDA** |
| --- | --- | --- |
| ENSMUSG00000032908 | -2.246964225 | 2.282571207 |
| ENSMUSG00000026480 | -1.327197368 | 1.897391921 |
| ENSMUSG00000042684 | -1.553515149 | 2.333116851 |
| ENSMUSG00000072109 | -1.962156547 | 1.393842421 |
| ENSMUSG00000118607 | -1.880853126 | 2.044450432 |
| ENSMUSG00000070034 | -1.42939115 | 1.653939069 |
| ENSMUSG00000070031 | -1.545973271 | 1.817659223 |
| ENSMUSG00000049608 | -2.089194166 | 1.780235909 |
| ENSMUSG00000097649 | -1.542975812 | 1.915771964 |
| ENSMUSG00000073530 | -1.659508108 | 1.896319698 |
| ENSMUSG00000026725 | -3.535611283 | 4.825337634 |
| ENSMUSG00000016529 | -4.147635512 | 2.527349439 |
| ENSMUSG00000101939 | -1.72647096 | 1.619236285 |
| ENSMUSG00000026288 | -1.548273212 | 2.074361536 |
| ENSMUSG00000078190 | -1.991180751 | 2.227129024 |
| ENSMUSG00000026009 | -1.998488955 | 1.41908359 |
| ENSMUSG00000026581 | -2.900137643 | 3.486434402 |
| ENSMUSG00000047180 | -1.310619258 | 1.522212778 |
| ENSMUSG00000046856 | -1.828254757 | 1.45529801 |
| ENSMUSG00000026285 | -2.79293815 | 3.204240733 |
| ENSMUSG00000026656 | -2.254552048 | 3.299228276 |
| ENSMUSG00000059089 | -3.633211613 | 10.19346837 |
| ENSMUSG00000059498 | -2.600652142 | 3.156333373 |
| ENSMUSG00000058715 | -2.451354828 | 3.181143099 |
| ENSMUSG00000006403 | -2.676373886 | 6.251666128 |
| ENSMUSG00000026073 | -1.681367143 | 2.118819454 |
| ENSMUSG00000059956 | -4.218790481 | 11.64275503 |
| ENSMUSG00000026395 | -1.621574848 | 1.819591454 |
| ENSMUSG00000026068 | -1.543880974 | 1.560462476 |
| ENSMUSG00000084228 | -3.50424682 | 1.780235909 |
| ENSMUSG00000044594 | -4.73049789 | 3.646813675 |
| ENSMUSG00000073602 | -3.390869741 | 8.848581239 |
| ENSMUSG00000067001 | -2.083040624 | 1.618512482 |
| ENSMUSG00000092572 | -2.884777885 | 8.775265285 |
| ENSMUSG00000004707 | -1.915520467 | 2.505166579 |
| ENSMUSG00000015355 | -2.403543841 | 2.613832441 |
| ENSMUSG00000026365 | -1.79727856 | 1.341995249 |
| ENSMUSG00000026358 | -1.941872277 | 4.555161418 |
| ENSMUSG00000026177 | -1.895904971 | 2.282571207 |
| ENSMUSG00000053318 | -1.693870198 | 1.522212778 |
| ENSMUSG00000005339 | -1.893017105 | 1.317487295 |
| ENSMUSG00000037872 | -1.472236319 | 1.391811451 |
| ENSMUSG00000037849 | -3.04708443 | 1.61910947 |
| ENSMUSG00000070501 | -3.442802771 | 5.048907459 |
| ENSMUSG00000073491 | -5.684608315 | 1.529735246 |
| ENSMUSG00000043263 | -2.154657911 | 2.971311868 |
| ENSMUSG00000066677 | -2.028040692 | 1.775038482 |
| ENSMUSG00000073489 | -1.629881855 | 1.690976954 |
| ENSMUSG00000090272 | -1.614532854 | 2.118819454 |
| ENSMUSG00000026536 | -1.800213627 | 1.9854842 |
| ENSMUSG00000039997 | -1.517438465 | 1.651260447 |
| ENSMUSG00000026535 | -1.999715576 | 4.004856452 |
| ENSMUSG00000103588 | -3.233613372 | 2.006759136 |
| ENSMUSG00000054203 | -1.696591072 | 1.30298091 |
| ENSMUSG00000006014 | -3.131451003 | 4.58333041 |
| ENSMUSG00000038147 | -1.868088856 | 2.252850634 |
| ENSMUSG00000078867 | -1.655325062 | 1.403518695 |
| ENSMUSG00000036040 | -1.792757989 | 1.782560775 |
| ENSMUSG00000025314 | -1.126048559 | 1.865687653 |
| ENSMUSG00000003283 | -1.704495489 | 4.057473855 |
| ENSMUSG00000070719 | -5.436346312 | 3.621205548 |
| ENSMUSG00000002111 | -1.580765794 | 2.07706579 |
| ENSMUSG00000026712 | -2.064319861 | 1.871029409 |
| ENSMUSG00000046470 | -2.347446898 | 4.825337634 |
| ENSMUSG00000060802 | -1.795540211 | 2.145707895 |
| ENSMUSG00000027219 | -2.121783434 | 1.634679327 |
| ENSMUSG00000079071 | -2.122452605 | 1.734016116 |
| ENSMUSG00000027639 | -1.362327686 | 2.392071464 |
| ENSMUSG00000027239 | -1.460398978 | 2.346622548 |
| ENSMUSG00000016024 | -1.458775818 | 1.42267851 |
| ENSMUSG00000026985 | -3.138201129 | 4.689750003 |
| ENSMUSG00000044103 | -3.19605849 | 4.813842265 |
| ENSMUSG00000026984 | -3.484149671 | 3.396178354 |
| ENSMUSG00000083172 | -3.987465718 | 1.41908359 |
| ENSMUSG00000060131 | -1.456388623 | 1.769487967 |
| ENSMUSG00000026840 | -1.415013889 | 1.343383835 |
| ENSMUSG00000001864 | -1.620489806 | 1.941687857 |
| ENSMUSG00000042845 | -2.823589167 | 4.555161418 |
| ENSMUSG00000017002 | -1.895087923 | 1.648067852 |
| ENSMUSG00000017652 | -1.876513722 | 2.556134386 |
| ENSMUSG00000027398 | -4.301139545 | 2.842368748 |
| ENSMUSG00000087362 | -2.307346206 | 2.08383447 |
| ENSMUSG00000036731 | -1.36806407 | 1.489461658 |
| ENSMUSG00000027670 | -3.198237159 | 1.657638214 |
| ENSMUSG00000027322 | -2.341013161 | 4.029490683 |
| ENSMUSG00000074577 | -1.458173181 | 2.214184371 |
| ENSMUSG00000026938 | -2.784017726 | 1.485008533 |
| ENSMUSG00000027078 | -1.662506621 | 3.344974393 |
| ENSMUSG00000050558 | -4.732316976 | 4.28806739 |
| ENSMUSG00000027074 | -1.569301557 | 1.403518695 |
| ENSMUSG00000027514 | -2.259012117 | 6.832312028 |
| ENSMUSG00000026928 | -1.552579337 | 4.063682662 |
| ENSMUSG00000047250 | -1.492025996 | 2.775458335 |
| ENSMUSG00000027456 | -1.93422193 | 2.365798156 |
| ENSMUSG00000053475 | -1.64567997 | 1.620688468 |
| ENSMUSG00000026832 | -1.521630128 | 1.485008533 |
| ENSMUSG00000027199 | -1.526123805 | 1.775038482 |
| ENSMUSG00000017057 | -1.152696166 | 1.988413648 |
| ENSMUSG00000033777 | -2.684425155 | 3.890832635 |
| ENSMUSG00000031372 | -1.4810831 | 1.382826199 |
| ENSMUSG00000053909 | -2.517261874 | 1.5409972 |
| ENSMUSG00000048621 | -1.898905703 | 1.42267851 |
| ENSMUSG00000031101 | -1.868164156 | 3.397130334 |
| ENSMUSG00000031165 | -1.506289311 | 2.316797318 |
| ENSMUSG00000067276 | -1.275731881 | 1.491259559 |
| ENSMUSG00000025058 | -2.262649306 | 2.819398336 |
| ENSMUSG00000031170 | -6.818727427 | 1.789848671 |
| ENSMUSG00000049775 | -1.199242899 | 1.406955611 |
| ENSMUSG00000040522 | -2.836034665 | 3.418119804 |
| ENSMUSG00000015340 | -2.169392304 | 2.981940426 |
| ENSMUSG00000044583 | -2.649588185 | 3.105397719 |
| ENSMUSG00000078122 | -2.131190409 | 2.819721075 |
| ENSMUSG00000044206 | -3.641491572 | 3.770541851 |
| ENSMUSG00000040138 | -2.161520239 | 1.9854372 |
| ENSMUSG00000023070 | -2.553243411 | 2.206464612 |
| ENSMUSG00000031304 | -1.271135864 | 2.206464612 |
| ENSMUSG00000001131 | -1.868500016 | 1.819591454 |
| ENSMUSG00000068122 | -2.163472219 | 2.504729266 |
| ENSMUSG00000054626 | -1.472719543 | 1.301748066 |
| ENSMUSG00000028037 | -3.146986624 | 15.59744507 |
| ENSMUSG00000039146 | -2.911130932 | 13.01461699 |
| ENSMUSG00000013707 | -1.843058529 | 3.410583049 |
| ENSMUSG00000028111 | -1.415312615 | 1.393773955 |
| ENSMUSG00000038642 | -2.654807183 | 6.04268868 |
| ENSMUSG00000038543 | -1.505490642 | 1.600647909 |
| ENSMUSG00000093507 | -1.713358579 | 2.576766762 |
| ENSMUSG00000015947 | -2.663023832 | 5.98475608 |
| ENSMUSG00000058897 | -1.695403666 | 1.954348757 |
| ENSMUSG00000074604 | -1.356805007 | 2.282571207 |
| ENSMUSG00000054690 | -1.386436411 | 1.721225506 |
| ENSMUSG00000095788 | -2.721813212 | 1.859495449 |
| ENSMUSG00000074677 | -2.681604966 | 1.474942142 |
| ENSMUSG00000078780 | -3.297553339 | 2.534474982 |
| ENSMUSG00000027848 | -1.786891895 | 2.320355861 |
| ENSMUSG00000036353 | -2.700885608 | 4.556960755 |
| ENSMUSG00000070465 | -2.069958612 | 3.621205548 |
| ENSMUSG00000091376 | -3.294986015 | 6.269468743 |
| ENSMUSG00000028268 | -1.596877849 | 2.028697895 |
| ENSMUSG00000063887 | -6.024917793 | 2.090068234 |
| ENSMUSG00000036960 | -1.955439163 | 1.9854842 |
| ENSMUSG00000040809 | -3.462020622 | 2.616542374 |
| ENSMUSG00000040747 | -2.419508299 | 3.630851599 |
| ENSMUSG00000011008 | -1.515244242 | 1.33481893 |
| ENSMUSG00000056071 | -3.425388113 | 2.562596365 |
| ENSMUSG00000043165 | -2.805002438 | 2.224109923 |
| ENSMUSG00000106143 | -3.291103889 | 2.626079045 |
| ENSMUSG00000078664 | -6.60783284 | 4.844941809 |
| ENSMUSG00000074445 | -5.586519398 | 11.21053484 |
| ENSMUSG00000042212 | -4.442649828 | 4.24216591 |
| ENSMUSG00000055030 | -3.104095382 | 1.65993911 |
| ENSMUSG00000046203 | -4.456653396 | 8.071454159 |
| ENSMUSG00000042157 | -3.409161663 | 1.711456638 |
| ENSMUSG00000048455 | -2.399223432 | 4.301212197 |
| ENSMUSG00000086848 | -3.100190054 | 4.063682662 |
| ENSMUSG00000057609 | -3.546278421 | 3.560087286 |
| ENSMUSG00000027923 | -3.783401765 | 3.621205548 |
| ENSMUSG00000068890 | -3.131529884 | 2.505166579 |
| ENSMUSG00000042092 | -3.478847801 | 4.236449901 |
| ENSMUSG00000103243 | -3.815130399 | 4.825337634 |
| ENSMUSG00000068889 | -3.821284936 | 4.650668561 |
| ENSMUSG00000042124 | -4.145748137 | 6.467469606 |
| ENSMUSG00000027919 | -4.376772394 | 6.289718996 |
| ENSMUSG00000049593 | -3.982422315 | 5.025560247 |
| ENSMUSG00000068888 | -4.09266945 | 2.875745026 |
| ENSMUSG00000068887 | -3.960921209 | 4.087951718 |
| ENSMUSG00000095870 | -4.023018814 | 9.359747195 |
| ENSMUSG00000059832 | -2.802267802 | 2.781870195 |
| ENSMUSG00000046676 | -3.110048482 | 2.690007249 |
| ENSMUSG00000090314 | -2.972979973 | 2.474482775 |
| ENSMUSG00000074433 | -7.971719827 | 5.559885741 |
| ENSMUSG00000068885 | -5.412609901 | 3.621205548 |
| ENSMUSG00000102439 | -4.089446035 | 6.049942578 |
| ENSMUSG00000041991 | -3.320661185 | 3.105397719 |
| ENSMUSG00000041984 | -2.269626816 | 4.618896223 |
| ENSMUSG00000028148 | -1.693651618 | 3.004447843 |
| ENSMUSG00000078680 | -3.149353681 | 3.992924594 |
| ENSMUSG00000078673 | -3.35604435 | 1.364954166 |
| ENSMUSG00000045672 | -1.769275451 | 2.626079045 |
| ENSMUSG00000028581 | -1.387320797 | 1.782916773 |
| ENSMUSG00000082699 | -5.63571274 | 1.634679327 |
| ENSMUSG00000049122 | -2.066156548 | 1.703816929 |
| ENSMUSG00000037731 | -2.139252311 | 3.70019705 |
| ENSMUSG00000028874 | -1.955290488 | 2.06208663 |
| ENSMUSG00000057751 | -1.785551457 | 2.019757999 |
| ENSMUSG00000000682 | -1.482088491 | 1.737933927 |
| ENSMUSG00000062585 | -1.666271523 | 1.698156253 |
| ENSMUSG00000036905 | -2.187788671 | 2.782586876 |
| ENSMUSG00000036896 | -2.376013366 | 2.908412448 |
| ENSMUSG00000036887 | -2.089826776 | 2.225727103 |
| ENSMUSG00000028645 | -1.218204638 | 2.406011442 |
| ENSMUSG00000078506 | -5.631924424 | 1.403518695 |
| ENSMUSG00000028599 | -1.38848479 | 2.248545041 |
| ENSMUSG00000028859 | -2.527651553 | 2.788382542 |
| ENSMUSG00000085020 | -3.811282931 | 1.309525524 |
| ENSMUSG00000028289 | -2.063446519 | 1.74504429 |
| ENSMUSG00000042228 | -1.566392655 | 1.639601605 |
| ENSMUSG00000085295 | -3.716562205 | 1.690976954 |
| ENSMUSG00000046593 | -6.231335555 | 2.101265977 |
| ENSMUSG00000028459 | -2.103001185 | 2.754455339 |
| ENSMUSG00000028341 | -1.478729649 | 1.403518695 |
| ENSMUSG00000029304 | -2.493086056 | 3.70019705 |
| ENSMUSG00000034438 | -1.857671961 | 1.852922784 |
| ENSMUSG00000040537 | -1.525542079 | 1.41908359 |
| ENSMUSG00000029236 | -5.302245937 | 1.65993911 |
| ENSMUSG00000060063 | -1.962700151 | 3.307242385 |
| ENSMUSG00000029254 | -1.761144143 | 2.229212305 |
| ENSMUSG00000039899 | -1.567474424 | 1.461316951 |
| ENSMUSG00000061259 | -3.180091253 | 1.436431077 |
| ENSMUSG00000072845 | -2.44509727 | 2.754455339 |
| ENSMUSG00000048163 | -1.666118813 | 3.536071729 |
| ENSMUSG00000035861 | -4.700733015 | 1.371275907 |
| ENSMUSG00000029082 | -2.24375731 | 3.992924594 |
| ENSMUSG00000067149 | -2.148726352 | 1.731498625 |
| ENSMUSG00000029561 | -2.104501878 | 4.556960755 |
| ENSMUSG00000041827 | -2.424808697 | 5.639429455 |
| ENSMUSG00000029373 | -2.122083182 | 1.466944056 |
| ENSMUSG00000029377 | -1.631819287 | 1.602648046 |
| ENSMUSG00000029417 | -1.775963626 | 1.454038279 |
| ENSMUSG00000034855 | -1.941787735 | 1.963138165 |
| ENSMUSG00000060183 | -2.693699786 | 2.510615083 |
| ENSMUSG00000023078 | -4.023579993 | 4.415848368 |
| ENSMUSG00000032690 | -2.394675485 | 6.571934244 |
| ENSMUSG00000032661 | -2.74095244 | 7.548175883 |
| ENSMUSG00000029605 | -1.413419888 | 2.505166579 |
| ENSMUSG00000053765 | -2.476714552 | 2.455703359 |
| ENSMUSG00000066861 | -2.50681035 | 7.262878559 |
| ENSMUSG00000052776 | -2.02226406 | 6.832312028 |
| ENSMUSG00000006642 | -3.862743747 | 4.876279348 |
| ENSMUSG00000047592 | -1.786815747 | 2.118819454 |
| ENSMUSG00000029322 | -3.106396022 | 2.875745026 |
| ENSMUSG00000035273 | -2.049628579 | 2.626079045 |
| ENSMUSG00000015950 | -1.905905033 | 3.71156779 |
| ENSMUSG00000030114 | -2.615714344 | 1.593989115 |
| ENSMUSG00000094335 | -7.075242735 | 4.029490683 |
| ENSMUSG00000105606 | -4.961351325 | 1.403518695 |
| ENSMUSG00000096515 | -7.643692567 | 3.780928359 |
| ENSMUSG00000094006 | -3.713807497 | 1.529735246 |
| ENSMUSG00000094930 | -6.58312301 | 3.048944834 |
| ENSMUSG00000076586 | -4.369729532 | 2.125046156 |
| ENSMUSG00000040552 | -2.700032909 | 3.387138497 |
| ENSMUSG00000076609 | -2.343653387 | 3.00052996 |
| ENSMUSG00000049037 | -2.245707518 | 3.985203252 |
| ENSMUSG00000043832 | -2.72206392 | 4.029490683 |
| ENSMUSG00000055874 | -4.217640817 | 7.262878559 |
| ENSMUSG00000030148 | -2.103539071 | 1.955383721 |
| ENSMUSG00000023349 | -2.527656601 | 3.471899125 |
| ENSMUSG00000030144 | -3.627985579 | 4.189502522 |
| ENSMUSG00000030142 | -3.477383792 | 2.008329489 |
| ENSMUSG00000008845 | -2.259336422 | 2.894659079 |
| ENSMUSG00000030268 | -1.873537508 | 2.257363286 |
| ENSMUSG00000023274 | -1.759981736 | 1.485008533 |
| ENSMUSG00000054435 | -1.412154461 | 1.65993911 |
| ENSMUSG00000030022 | -1.598675898 | 3.71156779 |
| ENSMUSG00000000627 | -1.363226752 | 1.361795278 |
| ENSMUSG00000029553 | -2.591271974 | 2.630288927 |
| ENSMUSG00000110390 | -2.424560296 | 2.120742626 |
| ENSMUSG00000047976 | -1.727280619 | 1.39489264 |
| ENSMUSG00000000182 | -3.427831204 | 1.650835878 |
| ENSMUSG00000030325 | -2.628303462 | 1.698156253 |
| ENSMUSG00000085786 | -1.663681992 | 1.439768192 |
| ENSMUSG00000089728 | -3.187378351 | 2.837200942 |
| ENSMUSG00000053063 | -2.398248671 | 2.056169669 |
| ENSMUSG00000029671 | -1.913956414 | 1.30298091 |
| ENSMUSG00000079293 | -2.404598611 | 1.877262293 |
| ENSMUSG00000050241 | -2.238486779 | 1.880018901 |
| ENSMUSG00000030165 | -1.682924776 | 1.690976954 |
| ENSMUSG00000014543 | -3.630985467 | 1.397815025 |
| ENSMUSG00000030077 | -1.492777219 | 1.491259559 |
| ENSMUSG00000067591 | -1.916578753 | 1.601527727 |
| ENSMUSG00000030187 | -2.62142882 | 1.525991515 |
| ENSMUSG00000029798 | -1.347029426 | 1.432841622 |
| ENSMUSG00000063415 | -2.335806934 | 2.372254672 |
| ENSMUSG00000030220 | -1.374325867 | 1.368949732 |
| ENSMUSG00000092164 | -1.914244994 | 1.365801518 |
| ENSMUSG00000041741 | -1.521720114 | 2.07706579 |
| ENSMUSG00000039070 | -1.565044173 | 1.403518695 |
| ENSMUSG00000038641 | -6.00178585 | 11.31757932 |
| ENSMUSG00000106889 | -7.04296361 | 3.621205548 |
| ENSMUSG00000029925 | -2.116200106 | 2.572276049 |
| ENSMUSG00000029915 | -2.055099443 | 2.144195412 |
| ENSMUSG00000030107 | -1.611493075 | 2.484436277 |
| ENSMUSG00000047735 | -1.41966435 | 1.441716644 |
| ENSMUSG00000029664 | -1.915439439 | 1.54014178 |
| ENSMUSG00000002588 | -4.020092357 | 2.497046238 |
| ENSMUSG00000078616 | -2.465076066 | 1.552060143 |
| ENSMUSG00000030921 | -1.804054097 | 2.819398336 |
| ENSMUSG00000057596 | -3.200511278 | 9.305172902 |
| ENSMUSG00000038623 | -1.548014055 | 1.759769068 |
| ENSMUSG00000004612 | -1.279407289 | 1.676170589 |
| ENSMUSG00000013367 | -1.269099348 | 1.41908359 |
| ENSMUSG00000057706 | -1.491834577 | 2.071642833 |
| ENSMUSG00000054046 | -1.569696225 | 2.038499945 |
| ENSMUSG00000067616 | -1.94512074 | 1.694374558 |
| ENSMUSG00000047884 | -3.656489073 | 10.19346837 |
| ENSMUSG00000064023 | -3.183598311 | 10.35258907 |
| ENSMUSG00000049685 | -1.861275148 | 1.439768192 |
| ENSMUSG00000063903 | -3.984367706 | 3.072237248 |
| ENSMUSG00000044701 | -4.162631897 | 1.804247689 |
| ENSMUSG00000030717 | -1.161255107 | 1.48701018 |
| ENSMUSG00000049130 | -2.28731708 | 2.248545041 |
| ENSMUSG00000058145 | -1.241357846 | 1.406133117 |
| ENSMUSG00000008193 | -2.299867259 | 1.542468286 |
| ENSMUSG00000109329 | -3.486566868 | 1.876117383 |
| ENSMUSG00000004328 | -2.177819645 | 1.907807583 |
| ENSMUSG00000066756 | -3.578003935 | 2.70477007 |
| ENSMUSG00000036111 | -1.186401167 | 1.534060334 |
| ENSMUSG00000025789 | -1.963973526 | 1.963138165 |
| ENSMUSG00000060791 | -1.909986059 | 1.823479205 |
| ENSMUSG00000044317 | -1.037927229 | 1.386075187 |
| ENSMUSG00000108801 | -1.635920824 | 1.475246443 |
| ENSMUSG00000058818 | -1.591268239 | 1.9854842 |
| ENSMUSG00000030787 | -2.074954507 | 1.730821828 |
| ENSMUSG00000055541 | -1.862893775 | 1.535739101 |
| ENSMUSG00000030786 | -1.578770631 | 1.376750374 |
| ENSMUSG00000030789 | -1.352525028 | 1.31881013 |
| ENSMUSG00000053175 | -1.165506705 | 1.819220033 |
| ENSMUSG00000081723 | -2.023531483 | 1.485008533 |
| ENSMUSG00000062524 | -2.76356314 | 3.24402777 |
| ENSMUSG00000030844 | -1.26018073 | 1.628709092 |
| ENSMUSG00000030798 | -1.664866649 | 2.556134386 |
| ENSMUSG00000053158 | -1.153842341 | 1.875291897 |
| ENSMUSG00000054342 | -1.772802376 | 2.638416193 |
| ENSMUSG00000046223 | -1.586536087 | 1.61910947 |

Table S7 KEGG enrichment results between the CON and MOD groups for transcriptomics

| **ID** | **Description** | **GeneRatio** | **BgRatio** | **p.adjust** | **Count** |
| --- | --- | --- | --- | --- | --- |
| hsa04610 | Complement and coagulation cascades | 10/85 | 79/7914 | 1.51047697952178e-06 | 10 |
| hsa05150 | Staphylococcus aureus infection | 9/85 | 96/7914 | 6.03529765968367e-05 | 9 |
| hsa04620 | Toll-like receptor signaling pathway | 9/85 | 104/7914 | 7.95253633482619e-05 | 9 |
| hsa04060 | Cytokine-cytokine receptor interaction | 14/85 | 294/7914 | 9.92831394954503e-05 | 14 |
| hsa00524 | Neomycin, kanamycin and gentamicin biosynthesis | 3/85 | 5/7914 | 0.000379167703527281 | 3 |
| hsa04062 | Chemokine signaling pathway | 10/85 | 189/7914 | 0.000844032409416118 | 10 |
| hsa00500 | Starch and sucrose metabolism | 5/85 | 36/7914 | 0.000847186928325398 | 5 |
| hsa04640 | Hematopoietic cell lineage | 7/85 | 98/7914 | 0.00147711852470371 | 7 |
| hsa00010 | Glycolysis / Gluconeogenesis | 6/85 | 68/7914 | 0.00147711852470371 | 6 |
| hsa04061 | Viral protein interaction with cytokine and cytokine receptor | 7/85 | 100/7914 | 0.00147822821960387 | 7 |
| hsa05152 | Tuberculosis | 9/85 | 179/7914 | 0.00174096300834072 | 9 |
| hsa04066 | HIF-1 signaling pathway | 7/85 | 109/7914 | 0.00212153698811882 | 7 |
| hsa00052 | Galactose metabolism | 4/85 | 31/7914 | 0.00387166822073705 | 4 |
| hsa05020 | Prion diseases | 4/85 | 35/7914 | 0.00579025442054991 | 4 |
| hsa04625 | C-type lectin receptor signaling pathway | 6/85 | 104/7914 | 0.00903428118701067 | 6 |
| hsa04145 | Phagosome | 7/85 | 152/7914 | 0.0115375271442857 | 7 |
| hsa05133 | Pertussis | 5/85 | 76/7914 | 0.0115375271442857 | 5 |
| hsa05140 | Leishmaniasis | 5/85 | 76/7914 | 0.0115375271442857 | 5 |
| hsa04973 | Carbohydrate digestion and absorption | 4/85 | 47/7914 | 0.0131798413343699 | 4 |
| hsa00520 | Amino sugar and nucleotide sugar metabolism | 4/85 | 48/7914 | 0.0135484404576983 | 4 |
| hsa04380 | Osteoclast differentiation | 6/85 | 128/7914 | 0.0188028422841636 | 6 |
| hsa04666 | Fc gamma R-mediated phagocytosis | 5/85 | 93/7914 | 0.0230368987288582 | 5 |
| hsa05162 | Measles | 6/85 | 138/7914 | 0.0249818560003889 | 6 |
| hsa00051 | Fructose and mannose metabolism | 3/85 | 33/7914 | 0.034689644109512 | 3 |
| hsa05221 | Acute myeloid leukemia | 4/85 | 67/7914 | 0.0352266359069506 | 4 |
| hsa05169 | Epstein-Barr virus infection | 7/85 | 201/7914 | 0.0352266359069506 | 7 |
| hsa05230 | Central carbon metabolism in cancer | 4/85 | 69/7914 | 0.037606252293039 | 4 |
| hsa05340 | Primary immunodeficiency | 3/85 | 38/7914 | 0.0442392281371104 | 3 |
| hsa01200 | Carbon metabolism | 5/85 | 117/7914 | 0.0461350446226593 | 5 |
| hsa05166 | Human T-cell leukemia virus 1 infection | 7/85 | 219/7914 | 0.0481154234878643 | 7 |
| hsa04612 | Antigen processing and presentation | 4/85 | 78/7914 | 0.0502558331560931 | 4 |
| hsa04621 | NOD-like receptor signaling pathway | 6/85 | 181/7914 | 0.063523767251636 | 6 |
| hsa04930 | Type II diabetes mellitus | 3/85 | 46/7914 | 0.063523767251636 | 3 |
| hsa05322 | Systemic lupus erythematosus | 5/85 | 133/7914 | 0.0661033650165678 | 5 |
| hsa05144 | Malaria | 3/85 | 49/7914 | 0.0710438719824302 | 3 |
| hsa04514 | Cell adhesion molecules (CAMs) | 5/85 | 147/7914 | 0.0923936071790712 | 5 |

Table S8 GO enrichment results between the CON and MOD groups for transcriptomics

| **term** | **enrichment** | **pvalue** | **count** | **class** |
| --- | --- | --- | --- | --- |
| defense response | 0.532258 | 2.45498503204766e-35 | 66 | BP |
| immune system process | 0.66129 | 5.93863081483495e-34 | 82 | BP |
| immune effector process | 0.443548 | 5.73250421542173e-32 | 55 | BP |
| immune response | 0.556452 | 8.88066012041858e-32 | 69 | BP |
| innate immune response | 0.346774 | 3.43529359962873e-25 | 43 | BP |
| regulation of immune system process | 0.427419 | 3.52555705501963e-24 | 53 | BP |
| regulation of immune response | 0.354839 | 1.16684631834601e-23 | 44 | BP |
| cell activation | 0.395161 | 1.26402191767695e-23 | 49 | BP |
| leukocyte activation | 0.362903 | 3.42331436854201e-22 | 45 | BP |
| response to external stimulus | 0.475806 | 6.54730383722446e-21 | 59 | BP |
| cytoplasmic vesicle part | 0.298387 | 1.19077844489189e-13 | 37 | CC |
| cytoplasmic vesicle | 0.362903 | 2.10656382550022e-13 | 45 | CC |
| intracellular vesicle | 0.362903 | 2.21300167618408e-13 | 45 | CC |
| vesicle | 0.475806 | 9.39263004742921e-13 | 59 | CC |
| plasma membrane part | 0.362903 | 1.10604888843718e-12 | 45 | CC |
| secretory granule | 0.209677 | 7.05656658612995e-12 | 26 | CC |
| cornified envelope | 0.072581 | 8.08513471814515e-12 | 9 | CC |
| whole membrane | 0.282258 | 4.14101249123452e-11 | 35 | CC |
| intrinsic component of plasma membrane | 0.241935 | 1.38834268227334e-10 | 30 | CC |
| secretory vesicle | 0.209677 | 1.39685638128783e-10 | 26 | CC |
| CXCR3 chemokine receptor binding | 0.040323 | 1.49402972287625e-11 | 5 | MF |
| signaling receptor binding | 0.258065 | 7.97206739965295e-10 | 32 | MF |
| carbohydrate binding | 0.112903 | 2.34277362313048e-09 | 14 | MF |
| CXCR chemokine receptor binding | 0.040323 | 3.66162309438102e-09 | 5 | MF |
| receptor regulator activity | 0.129032 | 4.08051526527478e-08 | 16 | MF |
| receptor ligand activity | 0.120968 | 1.06971520033252e-07 | 15 | MF |
| cytokine activity | 0.080645 | 1.19201488992325e-07 | 10 | MF |
| immunoglobulin receptor binding | 0.032258 | 2.72662566898496e-07 | 4 | MF |
| hexokinase activity | 0.024194 | 1.30506982704477e-06 | 3 | MF |
| chemokine activity | 0.040323 | 1.85280174632955e-06 | 5 | MF |

Table S9 958 genes in up mode between the MOD and YMPH groups

| **rownames(res)** | **log_2_FoldChange** | **-log_10_FDA** |
| --- | --- | --- |
| ENSMUSG00000101567 | 4.190180732 | 2.414985827 |
| ENSMUSG00000026482 | 1.525670696 | 3.605824571 |
| ENSMUSG00000026480 | 3.848708444 | 8.04125272 |
| ENSMUSG00000026043 | 1.839817684 | 1.739349328 |
| ENSMUSG00000026042 | 1.681464209 | 1.339502978 |
| ENSMUSG00000026479 | 2.129254898 | 3.177385335 |
| ENSMUSG00000073538 | 1.760558099 | 2.116081472 |
| ENSMUSG00000042684 | 2.087965208 | 1.636114533 |
| ENSMUSG00000101702 | 3.346295433 | 1.486465086 |
| ENSMUSG00000066800 | 2.090833033 | 4.186837223 |
| ENSMUSG00000038608 | 1.78284498 | 6.136895676 |
| ENSMUSG00000073535 | 2.514978032 | 1.498595507 |
| ENSMUSG00000062939 | 1.651369402 | 2.128969518 |
| ENSMUSG00000016918 | 1.718042097 | 2.395952848 |
| ENSMUSG00000045658 | 1.742805345 | 2.989695919 |
| ENSMUSG00000026601 | 1.76326198 | 1.525603918 |
| ENSMUSG00000118590 | 3.361868954 | 3.163932437 |
| ENSMUSG00000026104 | 3.07117796 | 5.254218317 |
| ENSMUSG00000060519 | 2.232836613 | 4.829907301 |
| ENSMUSG00000052477 | 2.371753785 | 2.712054516 |
| ENSMUSG00000118640 | 4.839537802 | 3.229481959 |
| ENSMUSG00000061852 | 4.486511727 | 2.525765578 |
| ENSMUSG00000089844 | 4.6442276 | 8.795920934 |
| ENSMUSG00000073631 | 5.447161456 | 2.591495191 |
| ENSMUSG00000072109 | 4.737207331 | 11.26871559 |
| ENSMUSG00000079457 | 5.873344337 | 2.052541148 |
| ENSMUSG00000079455 | 4.284510918 | 4.885284393 |
| ENSMUSG00000101751 | 3.53544274 | 1.405774052 |
| ENSMUSG00000118607 | 2.167324595 | 2.648188038 |
| ENSMUSG00000070034 | 3.739435531 | 10.0827145 |
| ENSMUSG00000090103 | 6.044293573 | 6.015437585 |
| ENSMUSG00000070031 | 4.156703183 | 10.24538447 |
| ENSMUSG00000090246 | 4.266186329 | 6.290921205 |
| ENSMUSG00000058665 | 1.76708316 | 4.759666079 |
| ENSMUSG00000026102 | 1.452264788 | 1.875401429 |
| ENSMUSG00000026222 | 3.003454265 | 13.21721077 |
| ENSMUSG00000052760 | 2.182251131 | 8.781155118 |
| ENSMUSG00000049608 | 3.681496367 | 7.006380306 |
| ENSMUSG00000025779 | 1.912360339 | 3.488564143 |
| ENSMUSG00000026509 | 1.048251767 | 2.579131195 |
| ENSMUSG00000041779 | 1.421931748 | 4.70319108 |
| ENSMUSG00000026253 | 5.111127571 | 1.566709042 |
| ENSMUSG00000045382 | 2.346929329 | 1.681628884 |
| ENSMUSG00000103308 | 2.71937584 | 1.582237526 |
| ENSMUSG00000039377 | 2.297774246 | 3.099615789 |
| ENSMUSG00000073530 | 1.044009143 | 2.270983795 |
| ENSMUSG00000038305 | 2.366886384 | 6.458348921 |
| ENSMUSG00000064294 | 2.579666649 | 1.311717705 |
| ENSMUSG00000026725 | 5.129431578 | 4.390492744 |
| ENSMUSG00000026420 | 6.971922534 | 7.550928854 |
| ENSMUSG00000016524 | 5.733818263 | 10.06914514 |
| ENSMUSG00000016529 | 5.386591004 | 7.858174284 |
| ENSMUSG00000042349 | 1.803212579 | 4.363863342 |
| ENSMUSG00000026031 | 1.216273598 | 1.989111721 |
| ENSMUSG00000026029 | 1.337840666 | 3.676621258 |
| ENSMUSG00000026288 | 3.18198661 | 6.603523809 |
| ENSMUSG00000026701 | 1.171039941 | 2.138411674 |
| ENSMUSG00000026700 | 3.226778932 | 1.498556262 |
| ENSMUSG00000000817 | 3.10948726 | 1.502811459 |
| ENSMUSG00000078190 | 1.715462124 | 1.801264134 |
| ENSMUSG00000091017 | 8.836809644 | 5.108143322 |
| ENSMUSG00000026628 | 3.266672249 | 5.124346331 |
| ENSMUSG00000103174 | 5.677919592 | 2.639163764 |
| ENSMUSG00000026442 | 2.017566738 | 5.768668544 |
| ENSMUSG00000062310 | 3.32928533 | 2.175197922 |
| ENSMUSG00000049866 | 1.35750108 | 1.319154906 |
| ENSMUSG00000103292 | 5.251742481 | 2.472120802 |
| ENSMUSG00000026126 | 2.295535617 | 4.771457392 |
| ENSMUSG00000026012 | 2.147198312 | 3.169796101 |
| ENSMUSG00000026009 | 2.964683217 | 8.319577239 |
| ENSMUSG00000037318 | 1.654574088 | 3.787236982 |
| ENSMUSG00000048126 | 1.877913452 | 1.446814709 |
| ENSMUSG00000026639 | 1.993132916 | 2.051930911 |
| ENSMUSG00000026582 | 2.716262694 | 3.890369534 |
| ENSMUSG00000026581 | 6.972227269 | 14.67891392 |
| ENSMUSG00000103089 | 7.664769025 | 3.421100984 |
| ENSMUSG00000009633 | 3.43913362 | 3.769209644 |
| ENSMUSG00000104348 | 4.504689612 | 4.117862032 |
| ENSMUSG00000026580 | 2.665672971 | 2.989114775 |
| ENSMUSG00000047180 | 3.299237628 | 6.522669264 |
| ENSMUSG00000037447 | 2.406980222 | 2.529324144 |
| ENSMUSG00000064246 | 2.065246328 | 1.819417743 |
| ENSMUSG00000007805 | 1.528473604 | 1.385759024 |
| ENSMUSG00000026459 | 3.115760709 | 1.432296381 |
| ENSMUSG00000026458 | 1.302995678 | 3.421032663 |
| ENSMUSG00000005763 | 1.677347519 | 1.791577587 |
| ENSMUSG00000026117 | 1.79906789 | 3.19190159 |
| ENSMUSG00000026271 | 4.419214568 | 9.658312202 |
| ENSMUSG00000031506 | 2.412066751 | 4.635834688 |
| ENSMUSG00000003051 | 3.259358842 | 3.187936709 |
| ENSMUSG00000084989 | 3.038949959 | 3.537048343 |
| ENSMUSG00000047793 | 2.135768119 | 3.394551539 |
| ENSMUSG00000060568 | 1.418226808 | 2.084776062 |
| ENSMUSG00000177156 | 1.576045937 | 4.048851192 |
| ENSMUSG00000197713 | 1.996037162 | 9.613843074 |
| ENSMUSG00000023122 | 2.213320702 | 11.0692959 |
| ENSMUSG00000021922 | 3.315244665 | 12.06306352 |
| ENSMUSG00000026285 | 4.281122256 | 7.55918559 |
| ENSMUSG00000038463 | 1.66946215 | 1.438555147 |
| ENSMUSG00000010925 | 1.873633912 | 4.073703708 |
| ENSMUSG00000040710 | 2.393980936 | 2.419681859 |
| ENSMUSG00000070524 | 6.56143529 | 9.309037594 |
| ENSMUSG00000038421 | 3.547480172 | 2.232162054 |
| ENSMUSG00000044768 | 1.216614382 | 2.457645449 |
| ENSMUSG00000026656 | 3.158779718 | 10.33085644 |
| ENSMUSG00000059089 | 6.881587088 | 36.37715969 |
| ENSMUSG00000059498 | 4.085035835 | 9.540038334 |
| ENSMUSG00000026335 | 1.200840684 | 2.456321199 |
| ENSMUSG00000058715 | 4.261991271 | 13.20105676 |
| ENSMUSG00000006403 | 4.559623617 | 14.86278875 |
| ENSMUSG00000097113 | 2.14911303 | 2.780211553 |
| ENSMUSG00000097448 | 4.797587051 | 1.41034225 |
| ENSMUSG00000026073 | 3.909417288 | 5.34163487 |
| ENSMUSG00000102975 | 6.277410787 | 3.886318282 |
| ENSMUSG00000026321 | 2.291002703 | 7.557041286 |
| ENSMUSG00000099843 | 1.328470897 | 1.616233904 |
| ENSMUSG00000026069 | 1.70599689 | 2.161570701 |
| ENSMUSG00000059956 | 5.106339263 | 2.970609071 |
| ENSMUSG00000026395 | 3.59191199 | 8.647920468 |
| ENSMUSG00000026068 | 3.95153611 | 8.564576369 |
| ENSMUSG00000084228 | 5.269077772 | 1.932924819 |
| ENSMUSG00000104291 | 2.510075698 | 2.760765382 |
| ENSMUSG00000073602 | 4.543876944 | 5.985531023 |
| ENSMUSG00000004709 | 1.659483432 | 1.823508336 |
| ENSMUSG00000062345 | 2.093280108 | 1.482588987 |
| ENSMUSG00000092572 | 2.688050339 | 2.332073694 |
| ENSMUSG00000097063 | 5.747584811 | 1.759840633 |
| ENSMUSG00000004707 | 3.02214121 | 7.415063781 |
| ENSMUSG00000038179 | 2.695574915 | 9.649533587 |
| ENSMUSG00000015355 | 2.608077238 | 5.12467777 |
| ENSMUSG00000026051 | 3.230207929 | 1.624257215 |
| ENSMUSG00000015316 | 2.656021672 | 4.554216749 |
| ENSMUSG00000033898 | 4.682636219 | 1.890188215 |
| ENSMUSG00000089991 | 1.580853297 | 1.530374906 |
| ENSMUSG00000026365 | 1.836575511 | 2.141242981 |
| ENSMUSG00000026180 | 6.733057438 | 12.28684915 |
| ENSMUSG00000048480 | 7.046859102 | 4.752695509 |
| ENSMUSG00000026358 | 3.92652374 | 8.934053262 |
| ENSMUSG00000073650 | 2.747507666 | 4.056475044 |
| ENSMUSG00000026177 | 5.091397552 | 7.830528753 |
| ENSMUSG00000053318 | 4.176250363 | 14.51691109 |
| ENSMUSG00000005339 | 1.554301177 | 3.534673417 |
| ENSMUSG00000037872 | 1.63206998 | 1.559704527 |
| ENSMUSG00000032487 | 6.18719884 | 8.366197256 |
| ENSMUSG00000097754 | 3.369559751 | 2.586452803 |
| ENSMUSG00000005338 | 1.946666919 | 2.522940619 |
| ENSMUSG00000037860 | 1.719150954 | 3.813789537 |
| ENSMUSG00000037849 | 5.88746815 | 10.46779601 |
| ENSMUSG00000070501 | 6.853386447 | 14.67891392 |
| ENSMUSG00000073491 | 8.657849882 | 6.843524784 |
| ENSMUSG00000043263 | 5.408998142 | 9.817878097 |
| ENSMUSG00000066677 | 4.534556994 | 7.307718308 |
| ENSMUSG00000073490 | 1.811057426 | 1.60951811 |
| ENSMUSG00000073489 | 3.232057876 | 4.161995021 |
| ENSMUSG00000090222 | 2.637324781 | 4.259941474 |
| ENSMUSG00000102187 | 6.784488569 | 8.644758561 |
| ENSMUSG00000090272 | 2.644405524 | 5.45631285 |
| ENSMUSG00000026536 | 3.03609633 | 4.771457392 |
| ENSMUSG00000039997 | 2.910897968 | 5.715179098 |
| ENSMUSG00000026535 | 3.60838078 | 13.50237614 |
| ENSMUSG00000103588 | 6.15490002 | 16.79323614 |
| ENSMUSG00000054203 | 2.060630477 | 2.711334724 |
| ENSMUSG00000050069 | 2.144257927 | 2.496758097 |
| ENSMUSG00000006014 | 5.767108178 | 21.55154836 |
| ENSMUSG00000039783 | 1.940832754 | 2.03911964 |
| ENSMUSG00000026204 | 4.560716696 | 7.749141167 |
| ENSMUSG00000038147 | 3.205000652 | 8.567688942 |
| ENSMUSG00000073492 | 3.450167489 | 2.817527824 |
| ENSMUSG00000015314 | 3.940048597 | 4.033047602 |
| ENSMUSG00000048865 | 2.90971871 | 7.608610203 |
| ENSMUSG00000042901 | 1.089099764 | 2.181706766 |
| ENSMUSG00000049353 | 2.36635715 | 1.96917083 |
| ENSMUSG00000027460 | 3.553102642 | 3.750464786 |
| ENSMUSG00000050600 | 2.227677254 | 1.471732494 |
| ENSMUSG00000032715 | 2.248280056 | 1.969499024 |
| ENSMUSG00000036067 | 3.989849049 | 7.511899717 |
| ENSMUSG00000026896 | 2.705221376 | 5.458392397 |
| ENSMUSG00000036040 | 2.42064179 | 4.054101051 |
| ENSMUSG00000060445 | 4.145268668 | 5.760576054 |
| ENSMUSG00000025314 | 2.397319067 | 6.15688318 |
| ENSMUSG00000075318 | 4.218861413 | 5.055749609 |
| ENSMUSG00000026837 | 2.038493423 | 1.528646836 |
| ENSMUSG00000027474 | 1.392386414 | 1.746531971 |
| ENSMUSG00000026835 | 7.272400712 | 3.223921579 |
| ENSMUSG00000003283 | 4.205907707 | 17.55380223 |
| ENSMUSG00000026833 | 1.334198157 | 2.405313425 |
| ENSMUSG00000070144 | 5.401255676 | 1.302111186 |
| ENSMUSG00000026829 | 1.891432133 | 1.842357576 |
| ENSMUSG00000063611 | 1.907859966 | 2.265422743 |
| ENSMUSG00000027293 | 1.607813289 | 4.562566678 |
| ENSMUSG00000049401 | 1.40216381 | 1.507796811 |
| ENSMUSG00000086109 | 2.147863303 | 1.311578322 |
| ENSMUSG00000027574 | 2.255238936 | 1.301200821 |
| ENSMUSG00000026809 | 2.061353404 | 1.327926397 |
| ENSMUSG00000035513 | 1.268154868 | 1.838636547 |
| ENSMUSG00000079499 | 1.503359399 | 3.605544874 |
| ENSMUSG00000002111 | 3.646922959 | 9.916303819 |
| ENSMUSG00000002100 | 3.452003542 | 3.517853946 |
| ENSMUSG00000061186 | 2.835536588 | 2.20870309 |
| ENSMUSG00000102196 | 7.341410502 | 2.732047769 |
| ENSMUSG00000027287 | 1.392048733 | 2.166643209 |
| ENSMUSG00000026770 | 1.68788367 | 2.74290518 |
| ENSMUSG00000027611 | 1.567982486 | 1.984408806 |
| ENSMUSG00000027580 | 3.111031492 | 7.488399171 |
| ENSMUSG00000027254 | 1.736175855 | 4.708463259 |
| ENSMUSG00000026728 | 1.626600376 | 2.783605111 |
| ENSMUSG00000026712 | 2.493716948 | 5.500080864 |
| ENSMUSG00000036949 | 6.145081074 | 2.375840619 |
| ENSMUSG00000002103 | 1.049280527 | 2.773653985 |
| ENSMUSG00000026737 | 1.599032621 | 3.294944633 |
| ENSMUSG00000037683 | 2.996240042 | 3.253334365 |
| ENSMUSG00000046470 | 2.575310584 | 4.161229664 |
| ENSMUSG00000075284 | 1.939332515 | 3.117373446 |
| ENSMUSG00000060802 | 2.015180398 | 7.677884575 |
| ENSMUSG00000033368 | 3.080012107 | 4.094305189 |
| ENSMUSG00000027219 | 3.901857819 | 7.476915744 |
| ENSMUSG00000081219 | 2.048548172 | 2.287137805 |
| ENSMUSG00000079071 | 3.627053265 | 6.467108329 |
| ENSMUSG00000027249 | 5.240895128 | 1.667207203 |
| ENSMUSG00000026786 | 2.396244266 | 5.613932468 |
| ENSMUSG00000027639 | 3.61912606 | 6.880442332 |
| ENSMUSG00000050368 | 2.155138439 | 4.338569428 |
| ENSMUSG00000015839 | 1.090002305 | 1.470391613 |
| ENSMUSG00000037820 | 2.708514565 | 1.751089271 |
| ENSMUSG00000039476 | 1.435718399 | 2.483049649 |
| ENSMUSG00000050737 | 1.926334293 | 4.046752101 |
| ENSMUSG00000075270 | 1.406669342 | 1.597314179 |
| ENSMUSG00000016024 | 2.506603443 | 8.159992317 |
| ENSMUSG00000027230 | 1.688319358 | 1.428850484 |
| ENSMUSG00000002732 | 1.542819416 | 1.715986069 |
| ENSMUSG00000027204 | 1.43100512 | 2.308668861 |
| ENSMUSG00000037754 | 1.799000203 | 2.508758458 |
| ENSMUSG00000044103 | 5.32767337 | 9.551287413 |
| ENSMUSG00000027208 | 1.706448456 | 2.724900941 |
| ENSMUSG00000026981 | 3.896704309 | 8.661408629 |
| ENSMUSG00000060131 | 3.267238421 | 8.649954455 |
| ENSMUSG00000027360 | 5.169351659 | 7.698857406 |
| ENSMUSG00000075027 | 3.709840628 | 3.071431151 |
| ENSMUSG00000042272 | 1.129079345 | 1.367606781 |
| ENSMUSG00000017697 | 1.753605989 | 2.363044159 |
| ENSMUSG00000027656 | 2.08318783 | 2.977058043 |
| ENSMUSG00000042845 | 4.273338157 | 2.77056693 |
| ENSMUSG00000027009 | 2.253383564 | 5.645298086 |
| ENSMUSG00000017002 | 5.56315022 | 11.60399073 |
| ENSMUSG00000098489 | 5.943446414 | 2.383939447 |
| ENSMUSG00000075014 | 2.862742739 | 1.899782128 |
| ENSMUSG00000005089 | 4.034705012 | 2.762096925 |
| ENSMUSG00000017737 | 5.299624749 | 8.050309205 |
| ENSMUSG00000027381 | 1.554005648 | 1.481706662 |
| ENSMUSG00000074813 | 2.750604693 | 6.389006311 |
| ENSMUSG00000079053 | 3.722826531 | 2.614042658 |
| ENSMUSG00000017652 | 3.840280124 | 14.44342038 |
| ENSMUSG00000027399 | 4.244467559 | 10.61958554 |
| ENSMUSG00000027398 | 9.618503098 | 26.55236064 |
| ENSMUSG00000074805 | 5.245728316 | 6.877950362 |
| ENSMUSG00000027082 | 1.884002606 | 5.122761751 |
| ENSMUSG00000037902 | 1.47816979 | 4.267137384 |
| ENSMUSG00000032698 | 1.627284828 | 2.466509283 |
| ENSMUSG00000026822 | 4.809059971 | 13.49629305 |
| ENSMUSG00000027670 | 4.681923208 | 5.936451318 |
| ENSMUSG00000018459 | 1.959216377 | 1.352403645 |
| ENSMUSG00000027661 | 1.805688876 | 4.807634544 |
| ENSMUSG00000027408 | 3.005590726 | 5.331881738 |
| ENSMUSG00000079442 | 2.604379012 | 3.657132201 |
| ENSMUSG00000026814 | 2.098299946 | 7.250012648 |
| ENSMUSG00000039621 | 1.923496238 | 4.239243099 |
| ENSMUSG00000059013 | 4.111625683 | 5.603164472 |
| ENSMUSG00000026956 | 1.419374827 | 3.157807338 |
| ENSMUSG00000039021 | 1.711373844 | 1.526705115 |
| ENSMUSG00000027316 | 2.179938163 | 1.769285501 |
| ENSMUSG00000036587 | 2.036383004 | 1.939336147 |
| ENSMUSG00000026797 | 1.012948028 | 1.824373504 |
| ENSMUSG00000039501 | 1.554792428 | 2.31578919 |
| ENSMUSG00000027318 | 1.97009241 | 1.583853191 |
| ENSMUSG00000027322 | 4.075698264 | 8.849890192 |
| ENSMUSG00000017929 | 2.908081635 | 4.759666079 |
| ENSMUSG00000042821 | 2.281294696 | 3.384523052 |
| ENSMUSG00000074577 | 1.315810398 | 2.006000319 |
| ENSMUSG00000038831 | 1.034951735 | 1.307766762 |
| ENSMUSG00000074971 | 1.823565845 | 1.619764181 |
| ENSMUSG00000026938 | 3.564470174 | 2.566560652 |
| ENSMUSG00000027333 | 2.046759125 | 2.241884176 |
| ENSMUSG00000023224 | 1.851659891 | 4.042492979 |
| ENSMUSG00000027078 | 3.564844355 | 8.123200955 |
| ENSMUSG00000050558 | 4.949633495 | 3.613028383 |
| ENSMUSG00000027345 | 6.254316655 | 1.507796811 |
| ENSMUSG00000044338 | 2.523496063 | 3.12979382 |
| ENSMUSG00000074570 | 2.54647468 | 3.400240067 |
| ENSMUSG00000026875 | 1.443087775 | 2.01959232 |
| ENSMUSG00000027514 | 5.737037236 | 13.54163824 |
| ENSMUSG00000074934 | 3.040729424 | 2.954106505 |
| ENSMUSG00000026928 | 2.364453929 | 5.878175705 |
| ENSMUSG00000026880 | 1.968825939 | 3.531244419 |
| ENSMUSG00000035778 | 1.109599414 | 1.820094294 |
| ENSMUSG00000026885 | 2.253618335 | 1.325507394 |
| ENSMUSG00000016256 | 2.012536046 | 7.094020569 |
| ENSMUSG00000040152 | 1.66297516 | 1.597314179 |
| ENSMUSG00000026890 | 2.342836091 | 8.229692158 |
| ENSMUSG00000040133 | 1.828942388 | 2.511854199 |
| ENSMUSG00000026921 | 1.566799794 | 3.279335862 |
| ENSMUSG00000026919 | 5.491219701 | 1.789956643 |
| ENSMUSG00000040061 | 2.456306671 | 4.85161939 |
| ENSMUSG00000074743 | 1.305944572 | 1.487415641 |
| ENSMUSG00000027435 | 2.707468509 | 3.835158675 |
| ENSMUSG00000040035 | 2.602002212 | 4.132113102 |
| ENSMUSG00000074736 | 2.846664013 | 3.872249004 |
| ENSMUSG00000068129 | 2.192423103 | 4.133783576 |
| ENSMUSG00000026866 | 2.152079403 | 1.632590076 |
| ENSMUSG00000049744 | 2.124765833 | 4.478815272 |
| ENSMUSG00000026872 | 1.723294175 | 2.762096925 |
| ENSMUSG00000036249 | 1.191227929 | 2.455115697 |
| ENSMUSG00000026946 | 2.761238281 | 5.468706718 |
| ENSMUSG00000053475 | 2.270888235 | 2.711334724 |
| ENSMUSG00000026832 | 2.981991688 | 5.207870785 |
| ENSMUSG00000026970 | 1.107860807 | 2.116972034 |
| ENSMUSG00000027199 | 2.42053356 | 5.804962035 |
| ENSMUSG00000033213 | 3.056739024 | 3.844973791 |
| ENSMUSG00000033965 | 1.382154631 | 2.201127694 |
| ENSMUSG00000017057 | 2.367980884 | 10.52477775 |
| ENSMUSG00000067768 | 1.999321462 | 3.40163499 |
| ENSMUSG00000031362 | 1.415558034 | 1.327926397 |
| ENSMUSG00000033777 | 5.270872356 | 16.77019379 |
| ENSMUSG00000052821 | 2.361366443 | 3.468391388 |
| ENSMUSG00000050921 | 1.973727063 | 2.320354291 |
| ENSMUSG00000054293 | 2.883290603 | 8.681809395 |
| ENSMUSG00000073008 | 2.473305525 | 4.674161597 |
| ENSMUSG00000025268 | 1.138392145 | 1.421427356 |
| ENSMUSG00000093916 | 2.36024538 | 1.350475176 |
| ENSMUSG00000048621 | 3.313787611 | 6.214611552 |
| ENSMUSG00000031378 | 1.099732065 | 1.433028317 |
| ENSMUSG00000034551 | 1.541486875 | 1.371963193 |
| ENSMUSG00000005696 | 2.633073236 | 2.240211866 |
| ENSMUSG00000031389 | 2.315498498 | 7.954426489 |
| ENSMUSG00000031390 | 2.757021305 | 3.192386798 |
| ENSMUSG00000031253 | 2.027691976 | 1.524566394 |
| ENSMUSG00000037010 | 4.209686158 | 9.119276327 |
| ENSMUSG00000031101 | 3.249459438 | 12.69136717 |
| ENSMUSG00000031264 | 2.951799599 | 8.332789433 |
| ENSMUSG00000031266 | 2.420514009 | 2.780866169 |
| ENSMUSG00000039521 | 2.19698662 | 1.302094952 |
| ENSMUSG00000055110 | 4.391246093 | 1.872266635 |
| ENSMUSG00000087365 | 1.989885764 | 2.023488066 |
| ENSMUSG00000031125 | 5.705238537 | 2.189902032 |
| ENSMUSG00000072945 | 6.280738242 | 2.690924393 |
| ENSMUSG00000032750 | 2.992652161 | 5.586898923 |
| ENSMUSG00000031402 | 1.19638829 | 2.472174222 |
| ENSMUSG00000009731 | 1.300503281 | 3.539688512 |
| ENSMUSG00000031165 | 2.949395331 | 7.830460368 |
| ENSMUSG00000031380 | 2.19617903 | 2.782508373 |
| ENSMUSG00000025059 | 2.159721984 | 3.033519168 |
| ENSMUSG00000081933 | 5.640046434 | 1.309711758 |
| ENSMUSG00000067276 | 1.870089446 | 2.789849428 |
| ENSMUSG00000025058 | 4.097250018 | 15.74621211 |
| ENSMUSG00000035277 | 3.688940105 | 1.367749727 |
| ENSMUSG00000031342 | 1.266429693 | 3.757513429 |
| ENSMUSG00000049775 | 1.715891181 | 2.923813843 |
| ENSMUSG00000040522 | 4.076741937 | 13.02587506 |
| ENSMUSG00000015340 | 3.76484918 | 14.41804144 |
| ENSMUSG00000044583 | 2.908990587 | 7.270945771 |
| ENSMUSG00000072844 | 3.085488089 | 2.472661039 |
| ENSMUSG00000031207 | 1.533738716 | 3.000192415 |
| ENSMUSG00000078122 | 3.865071918 | 11.77101183 |
| ENSMUSG00000044206 | 3.784196936 | 2.760224799 |
| ENSMUSG00000083307 | 1.737999938 | 3.528887602 |
| ENSMUSG00000031216 | 1.569602593 | 1.344624903 |
| ENSMUSG00000031304 | 3.087675352 | 18.86589348 |
| ENSMUSG00000001131 | 4.700247339 | 7.535247511 |
| ENSMUSG00000001128 | 2.489392109 | 4.516312902 |
| ENSMUSG00000068122 | 3.205776967 | 1.885728903 |
| ENSMUSG00000050232 | 2.228702811 | 2.012410676 |
| ENSMUSG00000079481 | 1.208362529 | 1.38813338 |
| ENSMUSG00000049191 | 1.672029142 | 2.514227275 |
| ENSMUSG00000086691 | 1.927117998 | 1.498566284 |
| ENSMUSG00000045092 | 1.536465181 | 1.728012527 |
| ENSMUSG00000028037 | 5.786298954 | 24.70435326 |
| ENSMUSG00000039146 | 5.295876112 | 18.84397885 |
| ENSMUSG00000027962 | 3.003864902 | 6.793909754 |
| ENSMUSG00000043300 | 1.587734765 | 1.415821168 |
| ENSMUSG00000044434 | 1.150750313 | 1.412090172 |
| ENSMUSG00000074637 | 1.615028523 | 1.354626696 |
| ENSMUSG00000106455 | 3.285890899 | 1.303380062 |
| ENSMUSG00000028128 | 1.515132616 | 1.978611211 |
| ENSMUSG00000028015 | 1.247711245 | 2.2303264 |
| ENSMUSG00000027712 | 1.034635762 | 1.443426071 |
| ENSMUSG00000013707 | 2.690007495 | 7.261265868 |
| ENSMUSG00000104554 | 7.691668407 | 5.814477657 |
| ENSMUSG00000027995 | 4.486009023 | 13.25923323 |
| ENSMUSG00000039234 | 1.708807641 | 4.640830626 |
| ENSMUSG00000093424 | 2.713151142 | 2.60608389 |
| ENSMUSG00000046561 | 2.546219272 | 3.009257306 |
| ENSMUSG00000038642 | 4.486768543 | 26.38456918 |
| ENSMUSG00000038612 | 1.361722556 | 1.670176107 |
| ENSMUSG00000028108 | 1.426586214 | 2.570238599 |
| ENSMUSG00000094392 | 1.91982157 | 3.40163499 |
| ENSMUSG00000053819 | 1.61611264 | 2.718253266 |
| ENSMUSG00000038543 | 2.520809527 | 5.6430963 |
| ENSMUSG00000015745 | 2.142623139 | 4.507431942 |
| ENSMUSG00000028028 | 1.672997177 | 3.510071156 |
| ENSMUSG00000059994 | 3.502116164 | 2.364930576 |
| ENSMUSG00000046688 | 2.029681818 | 3.701488077 |
| ENSMUSG00000015947 | 5.92678561 | 20.68921266 |
| ENSMUSG00000027737 | 2.811070191 | 1.768658214 |
| ENSMUSG00000104068 | 4.717451502 | 1.393980724 |
| ENSMUSG00000058952 | 3.262799483 | 1.491121672 |
| ENSMUSG00000027994 | 1.363169834 | 3.383170478 |
| ENSMUSG00000037161 | 2.672503937 | 4.130824703 |
| ENSMUSG00000005640 | 4.309417772 | 2.793908293 |
| ENSMUSG00000028071 | 2.263052609 | 3.052079019 |
| ENSMUSG00000057123 | 2.181335332 | 2.290068065 |
| ENSMUSG00000044468 | 1.93814996 | 3.741644152 |
| ENSMUSG00000097804 | 2.628771889 | 1.349568849 |
| ENSMUSG00000086564 | 2.718074009 | 1.727574055 |
| ENSMUSG00000027863 | 1.756704483 | 2.811894798 |
| ENSMUSG00000027859 | 3.205889204 | 6.221169636 |
| ENSMUSG00000054690 | 2.317123089 | 4.096355406 |
| ENSMUSG00000028159 | 1.55848111 | 3.598530328 |
| ENSMUSG00000078783 | 4.426906967 | 4.208437554 |
| ENSMUSG00000095788 | 5.363998804 | 16.28198063 |
| ENSMUSG00000095028 | 5.495228828 | 8.123200955 |
| ENSMUSG00000074677 | 5.115243721 | 13.20105676 |
| ENSMUSG00000078780 | 6.793529509 | 14.43932693 |
| ENSMUSG00000039519 | 3.13015116 | 9.315838465 |
| ENSMUSG00000028059 | 1.081674945 | 1.506778344 |
| ENSMUSG00000050075 | 2.269922271 | 3.865755501 |
| ENSMUSG00000027848 | 2.45362302 | 2.667321024 |
| ENSMUSG00000036381 | 1.538801202 | 2.672406 |
| ENSMUSG00000036362 | 3.211128801 | 9.259969189 |
| ENSMUSG00000036353 | 3.27324867 | 8.224357748 |
| ENSMUSG00000091376 | 3.567108618 | 1.350475176 |
| ENSMUSG00000044165 | 2.919745513 | 1.593555423 |
| ENSMUSG00000027843 | 1.952471044 | 2.648274265 |
| ENSMUSG00000003617 | 1.771665111 | 3.036081678 |
| ENSMUSG00000105504 | 5.927959092 | 7.4127472 |
| ENSMUSG00000040253 | 4.778467658 | 5.811013791 |
| ENSMUSG00000028268 | 5.779094517 | 7.50615501 |
| ENSMUSG00000040264 | 5.592620974 | 6.110625215 |
| ENSMUSG00000028270 | 4.618574701 | 5.364569227 |
| ENSMUSG00000032902 | 1.543687785 | 2.190482754 |
| ENSMUSG00000002233 | 1.449084913 | 1.739474333 |
| ENSMUSG00000002227 | 2.087266881 | 4.890571755 |
| ENSMUSG00000105652 | 6.041249991 | 1.327926397 |
| ENSMUSG00000028047 | 1.293281663 | 2.504046743 |
| ENSMUSG00000042766 | 1.630864089 | 2.504046743 |
| ENSMUSG00000027698 | 1.264793223 | 1.664703669 |
| ENSMUSG00000039304 | 2.073240193 | 2.587931928 |
| ENSMUSG00000000562 | 2.072280339 | 2.117761116 |
| ENSMUSG00000074342 | 2.880473323 | 5.503343523 |
| ENSMUSG00000039286 | 1.573620728 | 2.686208534 |
| ENSMUSG00000034640 | 2.271960824 | 2.278565123 |
| ENSMUSG00000036960 | 2.128081467 | 1.940588026 |
| ENSMUSG00000063779 | 6.562597447 | 1.414796132 |
| ENSMUSG00000028194 | 2.065676508 | 4.841306573 |
| ENSMUSG00000040747 | 5.158695845 | 12.4042956 |
| ENSMUSG00000047959 | 3.055030497 | 2.382901419 |
| ENSMUSG00000056145 | 2.432309971 | 6.147178504 |
| ENSMUSG00000011008 | 3.492158949 | 4.50592048 |
| ENSMUSG00000014599 | 2.867840151 | 8.446212714 |
| ENSMUSG00000027951 | 1.643696518 | 2.42179569 |
| ENSMUSG00000046280 | 1.692985444 | 2.656934214 |
| ENSMUSG00000027684 | 1.576416912 | 1.443727256 |
| ENSMUSG00000027940 | 1.212134269 | 1.684048662 |
| ENSMUSG00000040322 | 1.13400062 | 2.251339126 |
| ENSMUSG00000105746 | 2.46003027 | 2.137646639 |
| ENSMUSG00000027669 | 1.087082533 | 1.501683731 |
| ENSMUSG00000001020 | 2.987893562 | 2.757688331 |
| ENSMUSG00000056054 | 6.771776625 | 12.28094174 |
| ENSMUSG00000056071 | 8.510485509 | 17.91677656 |
| ENSMUSG00000078664 | 9.526789193 | 6.118144665 |
| ENSMUSG00000074445 | 7.9342503 | 8.833032987 |
| ENSMUSG00000042212 | 7.376323644 | 5.721024183 |
| ENSMUSG00000055030 | 5.724490225 | 3.87595924 |
| ENSMUSG00000050635 | 4.138139247 | 2.414985827 |
| ENSMUSG00000046203 | 7.050590669 | 10.5981555 |
| ENSMUSG00000046259 | 2.938242478 | 1.684048662 |
| ENSMUSG00000042157 | 7.143453885 | 5.54003713 |
| ENSMUSG00000027925 | 6.374389069 | 2.648188038 |
| ENSMUSG00000054215 | 4.647991269 | 2.370628108 |
| ENSMUSG00000048455 | 4.700486177 | 3.997870567 |
| ENSMUSG00000054325 | 3.844987717 | 1.541829137 |
| ENSMUSG00000042031 | 4.605009571 | 2.324512385 |
| ENSMUSG00000043472 | 3.812840749 | 2.058013362 |
| ENSMUSG00000068885 | 8.121569854 | 5.41111051 |
| ENSMUSG00000097761 | 4.782315605 | 1.510874934 |
| ENSMUSG00000028517 | 1.070059484 | 1.612350538 |
| ENSMUSG00000019055 | 1.444814076 | 2.583819215 |
| ENSMUSG00000000409 | 1.170341212 | 1.415367345 |
| ENSMUSG00000050493 | 2.588483633 | 4.973850411 |
| ENSMUSG00000029007 | 1.457752408 | 3.06624719 |
| ENSMUSG00000029005 | 1.711349789 | 1.843512749 |
| ENSMUSG00000028776 | 1.280646751 | 2.175634715 |
| ENSMUSG00000025743 | 2.54780317 | 18.37651786 |
| ENSMUSG00000045672 | 1.562848398 | 1.627253239 |
| ENSMUSG00000028581 | 2.934279489 | 8.360495917 |
| ENSMUSG00000061540 | 2.839734463 | 2.189902032 |
| ENSMUSG00000039158 | 1.828488375 | 6.73466688 |
| ENSMUSG00000028597 | 1.803928054 | 2.421415628 |
| ENSMUSG00000039936 | 2.515799744 | 6.150381137 |
| ENSMUSG00000084807 | 4.08250393 | 1.448006681 |
| ENSMUSG00000085213 | 1.924034677 | 2.157377902 |
| ENSMUSG00000028364 | 2.821120851 | 5.844575689 |
| ENSMUSG00000073821 | 3.809324222 | 5.662761702 |
| ENSMUSG00000028965 | 3.785301126 | 11.56846251 |
| ENSMUSG00000039005 | 2.872139617 | 4.123582095 |
| ENSMUSG00000056529 | 3.834892697 | 6.899517606 |
| ENSMUSG00000037731 | 5.136090012 | 17.36924465 |
| ENSMUSG00000028874 | 4.914440847 | 15.35011412 |
| ENSMUSG00000037553 | 1.093549199 | 1.44696098 |
| ENSMUSG00000028931 | 1.897681698 | 4.428413763 |
| ENSMUSG00000028717 | 1.542988091 | 2.85145002 |
| ENSMUSG00000000682 | 3.366127823 | 12.45402117 |
| ENSMUSG00000078238 | 1.283084413 | 1.915906177 |
| ENSMUSG00000012126 | 1.13578374 | 1.442274723 |
| ENSMUSG00000050105 | 1.718152479 | 2.109232284 |
| ENSMUSG00000042333 | 2.563585985 | 4.584269937 |
| ENSMUSG00000038172 | 1.192870959 | 2.10007437 |
| ENSMUSG00000050989 | 2.317182814 | 2.375115469 |
| ENSMUSG00000037306 | 1.783095132 | 2.529324144 |
| ENSMUSG00000066113 | 1.268618564 | 1.310927095 |
| ENSMUSG00000028494 | 1.512810253 | 1.880646945 |
| ENSMUSG00000037139 | 2.711870255 | 1.904149792 |
| ENSMUSG00000083430 | 6.274830837 | 2.530856418 |
| ENSMUSG00000062585 | 2.982939671 | 5.734070497 |
| ENSMUSG00000028497 | 2.178601916 | 2.989310519 |
| ENSMUSG00000087659 | 3.015926301 | 1.414796132 |
| ENSMUSG00000028680 | 2.230411246 | 1.727614266 |
| ENSMUSG00000054000 | 1.091352943 | 1.939336147 |
| ENSMUSG00000036905 | 2.901800622 | 3.625752536 |
| ENSMUSG00000036896 | 2.838144068 | 3.243861881 |
| ENSMUSG00000036887 | 2.852004807 | 3.076283545 |
| ENSMUSG00000028539 | 1.450564355 | 1.772050678 |
| ENSMUSG00000035692 | 4.431404994 | 14.51691109 |
| ENSMUSG00000033191 | 1.650289372 | 1.787012999 |
| ENSMUSG00000096351 | 1.890301626 | 1.805672666 |
| ENSMUSG00000028645 | 3.225120368 | 8.573445576 |
| ENSMUSG00000086683 | 2.750998069 | 1.491860172 |
| ENSMUSG00000085505 | 5.364446096 | 2.150222426 |
| ENSMUSG00000045349 | 3.546668291 | 6.087979714 |
| ENSMUSG00000028758 | 2.7996696 | 2.025859997 |
| ENSMUSG00000028641 | 1.599581562 | 4.665822538 |
| ENSMUSG00000028530 | 1.30735217 | 2.104965148 |
| ENSMUSG00000028525 | 2.523558366 | 1.830751579 |
| ENSMUSG00000070637 | 4.31451992 | 3.813789537 |
| ENSMUSG00000028583 | 2.578232924 | 3.040195829 |
| ENSMUSG00000078506 | 5.770170733 | 2.137959695 |
| ENSMUSG00000028599 | 3.5086484 | 8.541533523 |
| ENSMUSG00000078502 | 1.946762303 | 1.89046837 |
| ENSMUSG00000042677 | 1.432643641 | 1.814275624 |
| ENSMUSG00000028859 | 6.141587847 | 14.67891392 |
| ENSMUSG00000050234 | 1.540547211 | 2.613776264 |
| ENSMUSG00000028793 | 1.774226144 | 1.761260095 |
| ENSMUSG00000028367 | 1.226144908 | 5.263787141 |
| ENSMUSG00000028369 | 1.917407782 | 3.44095457 |
| ENSMUSG00000057280 | 3.379276208 | 2.568110467 |
| ENSMUSG00000038668 | 1.234868654 | 1.461863223 |
| ENSMUSG00000086150 | 2.158021551 | 2.020705194 |
| ENSMUSG00000028381 | 1.114059655 | 1.385901372 |
| ENSMUSG00000042228 | 3.669426887 | 8.58800781 |
| ENSMUSG00000055216 | 2.713296849 | 2.502727795 |
| ENSMUSG00000085295 | 7.410366085 | 12.05868985 |
| ENSMUSG00000028249 | 1.756434566 | 3.419272795 |
| ENSMUSG00000040296 | 2.431082123 | 6.870537983 |
| ENSMUSG00000046593 | 7.025585478 | 3.931791033 |
| ENSMUSG00000086071 | 5.998958495 | 2.872808753 |
| ENSMUSG00000071005 | 2.730015547 | 1.663670862 |
| ENSMUSG00000028459 | 2.233644941 | 1.748836518 |
| ENSMUSG00000028465 | 1.050654358 | 1.641226583 |
| ENSMUSG00000028480 | 2.434663999 | 3.904479999 |
| ENSMUSG00000028327 | 2.391614442 | 4.110254723 |
| ENSMUSG00000035539 | 4.907337238 | 1.827318191 |
| ENSMUSG00000035517 | 1.511873236 | 2.793908767 |
| ENSMUSG00000039853 | 1.535564459 | 3.304213208 |
| ENSMUSG00000039774 | 1.149290542 | 1.413449941 |
| ENSMUSG00000028341 | 3.077524343 | 2.585155363 |
| ENSMUSG00000015247 | 1.10080759 | 1.679221046 |
| ENSMUSG00000040659 | 2.14839122 | 4.871171344 |
| ENSMUSG00000052271 | 3.22908711 | 2.10278336 |
| ENSMUSG00000029470 | 1.463234479 | 2.232414361 |
| ENSMUSG00000029307 | 1.683868048 | 1.776508752 |
| ENSMUSG00000034438 | 4.635017383 | 8.159992317 |
| ENSMUSG00000029298 | 3.677663613 | 5.950561137 |
| ENSMUSG00000079363 | 4.118142839 | 5.780283541 |
| ENSMUSG00000105096 | 5.808352808 | 5.18978356 |
| ENSMUSG00000104713 | 5.238610937 | 5.651791751 |
| ENSMUSG00000029622 | 1.109512828 | 1.561458315 |
| ENSMUSG00000070858 | 2.119183922 | 3.080618052 |
| ENSMUSG00000054520 | 2.054909316 | 7.243187791 |
| ENSMUSG00000040584 | 1.514356669 | 1.369174399 |
| ENSMUSG00000029231 | 1.667440945 | 2.780866169 |
| ENSMUSG00000042817 | 1.325535325 | 1.350535237 |
| ENSMUSG00000062960 | 2.151311778 | 4.058573075 |
| ENSMUSG00000029648 | 2.136822237 | 4.825335704 |
| ENSMUSG00000045502 | 6.267286852 | 13.95344034 |
| ENSMUSG00000033467 | 1.515669139 | 3.213660701 |
| ENSMUSG00000104818 | 5.484155649 | 2.2303264 |
| ENSMUSG00000036596 | 2.62293734 | 1.940632866 |
| ENSMUSG00000060063 | 3.763656341 | 11.75934395 |
| ENSMUSG00000107317 | 2.207827457 | 2.762096925 |
| ENSMUSG00000029659 | 1.291510332 | 1.42592522 |
| ENSMUSG00000105376 | 2.317112151 | 1.444142608 |
| ENSMUSG00000029344 | 1.280569985 | 1.596903326 |
| ENSMUSG00000042249 | 1.050140245 | 1.421944497 |
| ENSMUSG00000039934 | 2.855125627 | 7.865642772 |
| ENSMUSG00000029254 | 2.5898083 | 2.884308754 |
| ENSMUSG00000039899 | 2.423222263 | 3.267427907 |
| ENSMUSG00000061259 | 4.192514895 | 1.839736812 |
| ENSMUSG00000048163 | 3.221168249 | 8.847110478 |
| ENSMUSG00000044017 | 1.361688995 | 1.521367327 |
| ENSMUSG00000039753 | 1.110479207 | 1.479403459 |
| ENSMUSG00000104728 | 2.256707893 | 1.35907403 |
| ENSMUSG00000106609 | 10.00135457 | 8.058546045 |
| ENSMUSG00000029082 | 4.938393889 | 14.24276792 |
| ENSMUSG00000029084 | 3.531125917 | 9.046833252 |
| ENSMUSG00000087281 | 3.834879689 | 2.351898038 |
| ENSMUSG00000067149 | 3.005067843 | 2.236190236 |
| ENSMUSG00000028978 | 1.881813335 | 3.622216325 |
| ENSMUSG00000029561 | 5.03616275 | 11.27935207 |
| ENSMUSG00000087477 | 6.102487202 | 6.522669264 |
| ENSMUSG00000041827 | 6.070699301 | 17.48550502 |
| ENSMUSG00000039706 | 1.834244878 | 1.303380062 |
| ENSMUSG00000038181 | 1.185615143 | 2.586452803 |
| ENSMUSG00000029371 | 8.013848493 | 9.119868086 |
| ENSMUSG00000029372 | 4.199424027 | 4.399582447 |
| ENSMUSG00000029373 | 3.701808241 | 4.475837507 |
| ENSMUSG00000029379 | 10.43773042 | 4.195439848 |
| ENSMUSG00000029380 | 5.138693323 | 7.142637932 |
| ENSMUSG00000058427 | 11.48322044 | 16.06521369 |
| ENSMUSG00000035020 | 1.777644849 | 1.638243367 |
| ENSMUSG00000072941 | 1.346674338 | 1.84481503 |
| ENSMUSG00000045790 | 1.199306588 | 1.819417743 |
| ENSMUSG00000039252 | 3.060063189 | 3.149629584 |
| ENSMUSG00000029409 | 3.772475326 | 2.395952848 |
| ENSMUSG00000029413 | 1.701226622 | 3.000192415 |
| ENSMUSG00000029417 | 5.230327981 | 12.41006067 |
| ENSMUSG00000034855 | 6.526531983 | 11.60800899 |
| ENSMUSG00000060183 | 6.477093067 | 14.54392582 |
| ENSMUSG00000039191 | 1.128521331 | 1.681867261 |
| ENSMUSG00000025746 | 5.677869817 | 3.730234724 |
| ENSMUSG00000029193 | 6.759322295 | 3.072482154 |
| ENSMUSG00000044576 | 2.371004644 | 1.655553999 |
| ENSMUSG00000044827 | 4.550964901 | 10.43638848 |
| ENSMUSG00000051498 | 3.714341941 | 8.057080266 |
| ENSMUSG00000029185 | 1.302241205 | 2.10278336 |
| ENSMUSG00000037913 | 4.112553151 | 3.868856318 |
| ENSMUSG00000023078 | 3.597563045 | 9.137313934 |
| ENSMUSG00000029484 | 1.131377766 | 1.430918204 |
| ENSMUSG00000029338 | 2.736516505 | 2.361497469 |
| ENSMUSG00000032690 | 4.49486319 | 10.44860635 |
| ENSMUSG00000107075 | 7.15050041 | 4.624267534 |
| ENSMUSG00000032661 | 6.715616397 | 27.08404378 |
| ENSMUSG00000029163 | 2.319378216 | 8.223216009 |
| ENSMUSG00000001166 | 1.233903397 | 1.556182297 |
| ENSMUSG00000029605 | 3.078822956 | 5.671265608 |
| ENSMUSG00000029161 | 1.894956261 | 1.530374906 |
| ENSMUSG00000001168 | 3.867182128 | 2.451114303 |
| ENSMUSG00000066861 | 4.995530761 | 16.77019379 |
| ENSMUSG00000052776 | 4.445236294 | 20.88347652 |
| ENSMUSG00000006642 | 3.889758369 | 3.210808806 |
| ENSMUSG00000029204 | 3.807061351 | 8.532382385 |
| ENSMUSG00000042726 | 2.063349148 | 1.940632866 |
| ENSMUSG00000092060 | 4.134867149 | 4.365256936 |
| ENSMUSG00000097840 | 4.427290893 | 5.094170965 |
| ENSMUSG00000106832 | 4.898907489 | 2.511854199 |
| ENSMUSG00000106925 | 6.430661324 | 2.422885896 |
| ENSMUSG00000042594 | 1.09846532 | 1.626367937 |
| ENSMUSG00000107355 | 3.282007527 | 4.845350877 |
| ENSMUSG00000040751 | 2.05384849 | 3.599285073 |
| ENSMUSG00000029674 | 1.003556804 | 1.742256881 |
| ENSMUSG00000039959 | 1.116607301 | 1.621316788 |
| ENSMUSG00000086656 | 5.970832083 | 2.557224108 |
| ENSMUSG00000029718 | 1.786625156 | 3.703821783 |
| ENSMUSG00000082088 | 2.134953911 | 2.286785033 |
| ENSMUSG00000046245 | 4.113403973 | 7.164069519 |
| ENSMUSG00000066684 | 3.429400223 | 6.221055185 |
| ENSMUSG00000066682 | 2.923127077 | 4.40390532 |
| ENSMUSG00000047592 | 2.865848374 | 4.255553644 |
| ENSMUSG00000109713 | 5.486504508 | 2.182955418 |
| ENSMUSG00000025854 | 1.89949842 | 1.414762134 |
| ENSMUSG00000044092 | 2.360105856 | 2.269153482 |
| ENSMUSG00000050022 | 1.315784877 | 2.019794409 |
| ENSMUSG00000028883 | 1.735381589 | 2.71734348 |
| ENSMUSG00000025534 | 1.525617421 | 2.495621192 |
| ENSMUSG00000029322 | 5.015845603 | 9.397570092 |
| ENSMUSG00000035273 | 2.978756345 | 6.767157566 |
| ENSMUSG00000015950 | 2.923244393 | 12.22692166 |
| ENSMUSG00000029126 | 1.071778688 | 1.575177133 |
| ENSMUSG00000005107 | 1.525475721 | 2.351898038 |
| ENSMUSG00000004846 | 1.062910734 | 1.463780901 |
| ENSMUSG00000037411 | 1.973139555 | 1.339157414 |
| ENSMUSG00000043279 | 1.154820729 | 1.430395447 |
| ENSMUSG00000037390 | 2.585488173 | 1.548991134 |
| ENSMUSG00000076474 | 5.231079971 | 2.039177085 |
| ENSMUSG00000018341 | 1.256542041 | 1.312523689 |
| ENSMUSG00000076480 | 4.144892288 | 1.820115816 |
| ENSMUSG00000030114 | 2.427532416 | 1.820094294 |
| ENSMUSG00000030054 | 6.314669957 | 2.785614795 |
| ENSMUSG00000030236 | 7.329464542 | 2.322490973 |
| ENSMUSG00000084927 | 6.775686062 | 1.949821335 |
| ENSMUSG00000030055 | 1.555918485 | 1.631507043 |
| ENSMUSG00000076490 | 1.552415516 | 3.833130647 |
| ENSMUSG00000105606 | 5.917350716 | 2.34273519 |
| ENSMUSG00000094420 | 3.769080475 | 1.498556262 |
| ENSMUSG00000076540 | 9.2807924 | 6.840118454 |
| ENSMUSG00000076549 | 4.196711571 | 1.735654886 |
| ENSMUSG00000094006 | 4.774605106 | 1.947122394 |
| ENSMUSG00000076563 | 6.286154188 | 3.581677418 |
| ENSMUSG00000076580 | 6.474682185 | 4.033518418 |
| ENSMUSG00000040627 | 5.40884252 | 1.483345415 |
| ENSMUSG00000090192 | 6.171999223 | 4.161995021 |
| ENSMUSG00000089931 | 3.65690814 | 1.61437807 |
| ENSMUSG00000076586 | 4.198537562 | 1.742256881 |
| ENSMUSG00000094797 | 5.115526236 | 1.870509262 |
| ENSMUSG00000040613 | 1.581755384 | 4.541189903 |
| ENSMUSG00000030117 | 3.492063408 | 2.144048308 |
| ENSMUSG00000079477 | 1.131642712 | 1.512342692 |
| ENSMUSG00000040552 | 4.434598677 | 11.67317463 |
| ENSMUSG00000076609 | 4.204261934 | 3.458318439 |
| ENSMUSG00000049037 | 3.280999998 | 15.62460326 |
| ENSMUSG00000043832 | 3.626632538 | 19.18746075 |
| ENSMUSG00000059639 | 4.145146378 | 1.942920169 |
| ENSMUSG00000030148 | 3.312550541 | 13.23993055 |
| ENSMUSG00000023349 | 4.792560989 | 11.48305292 |
| ENSMUSG00000030144 | 8.602440697 | 18.62486977 |
| ENSMUSG00000053044 | 2.838821759 | 2.106731951 |
| ENSMUSG00000030142 | 9.31239529 | 17.26582527 |
| ENSMUSG00000108132 | 2.744620816 | 2.243085059 |
| ENSMUSG00000053977 | 3.057042689 | 3.113252532 |
| ENSMUSG00000008845 | 1.882184879 | 2.722315046 |
| ENSMUSG00000079575 | 1.161332604 | 1.374775092 |
| ENSMUSG00000048473 | 5.452023564 | 1.509988397 |
| ENSMUSG00000055172 | 1.938410564 | 4.825955908 |
| ENSMUSG00000038521 | 2.050110882 | 3.771421624 |
| ENSMUSG00000107568 | 2.303841111 | 1.499296559 |
| ENSMUSG00000098470 | 1.65589076 | 1.865657837 |
| ENSMUSG00000079343 | 2.138071079 | 4.682806711 |
| ENSMUSG00000030275 | 1.219520916 | 2.282011362 |
| ENSMUSG00000004266 | 2.111917827 | 4.268458944 |
| ENSMUSG00000004267 | 2.092777315 | 3.938456181 |
| ENSMUSG00000030268 | 3.533160929 | 17.8127692 |
| ENSMUSG00000023191 | 1.329327501 | 1.521367327 |
| ENSMUSG00000030263 | 1.734284348 | 3.761705479 |
| ENSMUSG00000023274 | 2.753902782 | 3.698802507 |
| ENSMUSG00000030124 | 1.191802507 | 1.775764791 |
| ENSMUSG00000009281 | 2.012559291 | 2.624628072 |
| ENSMUSG00000064262 | 1.263196868 | 2.668590275 |
| ENSMUSG00000054435 | 2.24850087 | 3.42074387 |
| ENSMUSG00000108368 | 3.252705944 | 2.435607396 |
| ENSMUSG00000043931 | 3.035455864 | 1.565657116 |
| ENSMUSG00000090019 | 1.733013849 | 3.892220847 |
| ENSMUSG00000043505 | 2.015610964 | 4.397019996 |
| ENSMUSG00000108298 | 3.09901462 | 1.535326692 |
| ENSMUSG00000039264 | 1.933791501 | 3.333464304 |
| ENSMUSG00000064080 | 1.728025564 | 1.56724374 |
| ENSMUSG00000079523 | 1.545815696 | 3.491120705 |
| ENSMUSG00000034037 | 1.295804296 | 1.474144127 |
| ENSMUSG00000059654 | 5.702279655 | 3.711481707 |
| ENSMUSG00000030022 | 1.803410224 | 2.280535782 |
| ENSMUSG00000048636 | 2.778264914 | 1.728133098 |
| ENSMUSG00000030043 | 2.130916405 | 4.758111828 |
| ENSMUSG00000038213 | 2.467478623 | 1.791922864 |
| ENSMUSG00000030336 | 1.483161381 | 3.080618052 |
| ENSMUSG00000000627 | 1.688023846 | 2.762335683 |
| ENSMUSG00000068335 | 1.519275908 | 3.869013376 |
| ENSMUSG00000041390 | 1.06488621 | 1.84343237 |
| ENSMUSG00000101191 | 3.054068778 | 2.518831999 |
| ENSMUSG00000029553 | 4.488939205 | 10.69883339 |
| ENSMUSG00000038301 | 2.203518553 | 3.073808595 |
| ENSMUSG00000000182 | 8.000208644 | 5.600208695 |
| ENSMUSG00000059182 | 1.410806537 | 2.875793158 |
| ENSMUSG00000030353 | 2.238306792 | 2.286785033 |
| ENSMUSG00000107526 | 4.765154249 | 1.371963193 |
| ENSMUSG00000107894 | 2.318536943 | 1.434332848 |
| ENSMUSG00000032712 | 1.312926734 | 1.74596119 |
| ENSMUSG00000038074 | 1.363959483 | 3.95810735 |
| ENSMUSG00000085786 | 2.774734798 | 6.484531742 |
| ENSMUSG00000089728 | 4.700063984 | 4.980973147 |
| ENSMUSG00000030157 | 1.20939014 | 1.397037543 |
| ENSMUSG00000030156 | 2.38649773 | 2.011267225 |
| ENSMUSG00000047720 | 4.51831304 | 1.39688102 |
| ENSMUSG00000053063 | 3.854507929 | 13.81208416 |
| ENSMUSG00000030158 | 2.759190644 | 2.655554681 |
| ENSMUSG00000030159 | 2.270171 | 1.51334428 |
| ENSMUSG00000029671 | 2.286494366 | 3.572312619 |
| ENSMUSG00000079293 | 6.528947532 | 24.6238437 |
| ENSMUSG00000030162 | 2.552867613 | 2.978834686 |
| ENSMUSG00000050241 | 2.880719079 | 5.557480611 |
| ENSMUSG00000030165 | 2.659409934 | 4.6055652 |
| ENSMUSG00000030149 | 2.998652314 | 12.46477093 |
| ENSMUSG00000052736 | 2.661666279 | 3.479081074 |
| ENSMUSG00000030167 | 3.026375154 | 4.910179465 |
| ENSMUSG00000107715 | 3.115161209 | 2.332073694 |
| ENSMUSG00000043932 | 2.521654802 | 2.762335683 |
| ENSMUSG00000014543 | 6.789962304 | 5.596222465 |
| ENSMUSG00000030077 | 2.212402527 | 1.917622581 |
| ENSMUSG00000067591 | 2.896938857 | 5.90079655 |
| ENSMUSG00000061969 | 6.556019102 | 1.592552745 |
| ENSMUSG00000030187 | 6.819534945 | 17.55380223 |
| ENSMUSG00000029798 | 4.302519318 | 6.467108329 |
| ENSMUSG00000037788 | 1.111994724 | 2.012410676 |
| ENSMUSG00000029802 | 1.155530125 | 1.904724416 |
| ENSMUSG00000063415 | 2.168018233 | 2.573464113 |
| ENSMUSG00000108068 | 2.626834836 | 3.090415404 |
| ENSMUSG00000057604 | 3.052342404 | 1.528646836 |
| ENSMUSG00000030220 | 2.525237321 | 8.5815298 |
| ENSMUSG00000003500 | 1.026174444 | 2.435607396 |
| ENSMUSG00000079652 | 3.388264764 | 3.090415404 |
| ENSMUSG00000030223 | 1.899749369 | 4.816007192 |
| ENSMUSG00000029771 | 2.303232793 | 4.32189028 |
| ENSMUSG00000060477 | 2.192997777 | 2.694890339 |
| ENSMUSG00000108154 | 3.494049817 | 1.350409615 |
| ENSMUSG00000030047 | 2.389256672 | 3.531244419 |
| ENSMUSG00000051855 | 3.280473415 | 3.291994694 |
| ENSMUSG00000030123 | 1.454695368 | 1.682155895 |
| ENSMUSG00000025608 | 2.032225872 | 4.561940813 |
| ENSMUSG00000086763 | 5.383705405 | 2.433897387 |
| ENSMUSG00000029762 | 2.255124004 | 8.323613413 |
| ENSMUSG00000038641 | 7.137187408 | 1.655202268 |
| ENSMUSG00000106889 | 8.325838579 | 4.23514286 |
| ENSMUSG00000029826 | 1.374805613 | 4.841306573 |
| ENSMUSG00000029925 | 3.384612018 | 12.02326889 |
| ENSMUSG00000100121 | 5.30052887 | 1.874927508 |
| ENSMUSG00000038507 | 2.835941753 | 4.094305189 |
| ENSMUSG00000073144 | 1.917410269 | 1.595980482 |
| ENSMUSG00000029923 | 1.262305714 | 2.476036982 |
| ENSMUSG00000038456 | 1.296964465 | 1.974095271 |
| ENSMUSG00000085058 | 3.422169788 | 4.006251834 |
| ENSMUSG00000029915 | 4.974816456 | 14.61680296 |
| ENSMUSG00000068587 | 5.610450791 | 6.394557438 |
| ENSMUSG00000002897 | 1.582579048 | 1.900691406 |
| ENSMUSG00000004446 | 1.572145669 | 3.000192415 |
| ENSMUSG00000030107 | 4.35805899 | 8.456854362 |
| ENSMUSG00000030109 | 2.097633048 | 1.368195067 |
| ENSMUSG00000000693 | 2.720433051 | 9.136769477 |
| ENSMUSG00000029844 | 2.231929716 | 2.993489217 |
| ENSMUSG00000107017 | 1.920598254 | 1.721259405 |
| ENSMUSG00000106734 | 2.636390452 | 4.943876772 |
| ENSMUSG00000107215 | 2.398609129 | 3.942223039 |
| ENSMUSG00000047735 | 3.309560763 | 13.5198764 |
| ENSMUSG00000029664 | 2.911390636 | 3.41906218 |
| ENSMUSG00000032766 | 1.520120118 | 1.514104986 |
| ENSMUSG00000107603 | 4.149611813 | 2.013068306 |
| ENSMUSG00000092035 | 1.411226296 | 1.555505253 |
| ENSMUSG00000002588 | 2.727028592 | 1.327926397 |
| ENSMUSG00000061762 | 2.824131424 | 1.947193592 |
| ENSMUSG00000046178 | 2.252857039 | 1.615134971 |
| ENSMUSG00000108059 | 6.676093209 | 4.991387263 |
| ENSMUSG00000001156 | 2.733111276 | 3.443950245 |
| ENSMUSG00000108291 | 2.360976692 | 1.884779408 |
| ENSMUSG00000029994 | 1.353132549 | 3.429047062 |
| ENSMUSG00000072244 | 1.489144533 | 3.113823977 |
| ENSMUSG00000056144 | 3.454498563 | 6.356516678 |
| ENSMUSG00000030742 | 1.557709872 | 1.941669991 |
| ENSMUSG00000090215 | 3.230327552 | 4.10735653 |
| ENSMUSG00000057143 | 2.116326315 | 3.637658113 |
| ENSMUSG00000052749 | 6.418292217 | 13.85614699 |
| ENSMUSG00000078616 | 5.882168705 | 7.589010907 |
| ENSMUSG00000110114 | 5.807757943 | 2.204480702 |
| ENSMUSG00000030921 | 4.234089864 | 10.83954927 |
| ENSMUSG00000057596 | 6.34771751 | 13.40044814 |
| ENSMUSG00000070526 | 2.377067725 | 1.978348319 |
| ENSMUSG00000002603 | 1.705872603 | 1.916432483 |
| ENSMUSG00000038623 | 1.942085671 | 3.740401268 |
| ENSMUSG00000004612 | 2.360291056 | 4.12520903 |
| ENSMUSG00000013353 | 6.66212595 | 2.978834686 |
| ENSMUSG00000108695 | 9.281446089 | 8.160575903 |
| ENSMUSG00000004609 | 3.448342287 | 5.100278207 |
| ENSMUSG00000030474 | 4.289744523 | 4.889095672 |
| ENSMUSG00000054046 | 3.043872035 | 2.811894798 |
| ENSMUSG00000047884 | 3.913286597 | 3.037598697 |
| ENSMUSG00000064023 | 2.973393167 | 4.656312842 |
| ENSMUSG00000031070 | 1.94334061 | 2.54071042 |
| ENSMUSG00000006948 | 5.217807145 | 1.369650574 |
| ENSMUSG00000052353 | 2.124560951 | 3.122230123 |
| ENSMUSG00000063903 | 4.676535641 | 3.314740314 |
| ENSMUSG00000030720 | 1.308302636 | 2.052541148 |
| ENSMUSG00000042759 | 3.368267178 | 8.402922853 |
| ENSMUSG00000044701 | 7.027614354 | 6.390558268 |
| ENSMUSG00000109341 | 6.328620436 | 2.72183214 |
| ENSMUSG00000030707 | 3.081449947 | 9.892014542 |
| ENSMUSG00000074361 | 4.077464381 | 3.051865782 |
| ENSMUSG00000049130 | 4.603812835 | 6.50567651 |
| ENSMUSG00000030882 | 1.408271103 | 1.505831447 |
| ENSMUSG00000040466 | 1.140211095 | 2.402930797 |
| ENSMUSG00000008193 | 2.972558156 | 2.748862399 |
| ENSMUSG00000030562 | 1.958511688 | 1.638569561 |
| ENSMUSG00000109539 | 1.778645532 | 2.415176192 |
| ENSMUSG00000109685 | 1.859082507 | 1.42592522 |
| ENSMUSG00000086513 | 4.302387215 | 7.082245664 |
| ENSMUSG00000078606 | 3.493799242 | 6.709517128 |
| ENSMUSG00000099241 | 4.572614739 | 3.106810261 |
| ENSMUSG00000095649 | 4.095388077 | 3.135882279 |
| ENSMUSG00000045868 | 4.224478306 | 5.375546583 |
| ENSMUSG00000095457 | 4.618937876 | 5.223585189 |
| ENSMUSG00000053541 | 3.4199616 | 7.146077469 |
| ENSMUSG00000063286 | 5.422810499 | 19.8673574 |
| ENSMUSG00000073902 | 6.608317245 | 11.74402818 |
| ENSMUSG00000039405 | 1.572872248 | 1.379724607 |
| ENSMUSG00000002204 | 2.907175076 | 5.584401448 |
| ENSMUSG00000030681 | 1.906070477 | 2.465767106 |
| ENSMUSG00000043017 | 2.159456521 | 4.026869736 |
| ENSMUSG00000045165 | 1.711039529 | 3.150564112 |
| ENSMUSG00000051457 | 1.869641195 | 2.989310519 |
| ENSMUSG00000097585 | 2.003688634 | 2.278030654 |
| ENSMUSG00000108897 | 2.689972445 | 1.967625653 |
| ENSMUSG00000032776 | 1.828805535 | 2.15695918 |
| ENSMUSG00000108545 | 2.446372653 | 1.419882834 |
| ENSMUSG00000003505 | 8.398537844 | 5.867415501 |
| ENSMUSG00000030413 | 3.567311027 | 6.517127379 |
| ENSMUSG00000030830 | 3.527731439 | 7.961247193 |
| ENSMUSG00000044786 | 2.176053949 | 2.718808789 |
| ENSMUSG00000078817 | 4.458741628 | 1.739158699 |
| ENSMUSG00000097415 | 1.629673718 | 7.901433762 |
| ENSMUSG00000060791 | 3.149635758 | 6.911466088 |
| ENSMUSG00000053338 | 5.88962931 | 10.86797918 |
| ENSMUSG00000039361 | 1.061258481 | 1.328160896 |
| ENSMUSG00000109498 | 2.586305324 | 2.962675962 |
| ENSMUSG00000037463 | 1.342624729 | 1.661302647 |
| ENSMUSG00000044317 | 1.491868798 | 5.042328342 |
| ENSMUSG00000030403 | 1.487060015 | 2.122640319 |
| ENSMUSG00000108801 | 1.485441349 | 2.65691275 |
| ENSMUSG00000030402 | 2.533754954 | 1.51844468 |
| ENSMUSG00000101585 | 2.468301315 | 3.526071475 |
| ENSMUSG00000039236 | 3.015755763 | 6.876699234 |
| ENSMUSG00000031015 | 1.088323743 | 2.682774755 |
| ENSMUSG00000051735 | 2.288962631 | 6.603149011 |
| ENSMUSG00000058818 | 3.304438722 | 8.458844444 |
| ENSMUSG00000030790 | 2.737834019 | 5.240349413 |
| ENSMUSG00000081665 | 4.285264408 | 8.002060409 |
| ENSMUSG00000080917 | 3.458168705 | 3.412617183 |
| ENSMUSG00000011263 | 1.294649297 | 1.771381319 |
| ENSMUSG00000030787 | 3.072100428 | 4.100554041 |
| ENSMUSG00000074419 | 2.869290831 | 3.834229416 |
| ENSMUSG00000089942 | 4.5076196 | 6.624570007 |
| ENSMUSG00000074420 | 2.847103738 | 1.53891953 |
| ENSMUSG00000074417 | 4.580264928 | 7.868845337 |
| ENSMUSG00000030427 | 4.110584803 | 10.25119627 |
| ENSMUSG00000037337 | 1.451281827 | 2.836501615 |
| ENSMUSG00000055541 | 3.698614602 | 14.59233482 |
| ENSMUSG00000002983 | 1.367150841 | 3.447738338 |
| ENSMUSG00000030786 | 4.336604053 | 11.51807709 |
| ENSMUSG00000061119 | 1.360228105 | 1.550281963 |
| ENSMUSG00000030789 | 2.687265346 | 3.12573597 |
| ENSMUSG00000019539 | 1.651462925 | 1.373669094 |
| ENSMUSG00000053175 | 2.887162018 | 7.511899717 |
| ENSMUSG00000087593 | 2.343498774 | 2.1587597 |
| ENSMUSG00000109424 | 3.909779472 | 2.122640319 |
| ENSMUSG00000014686 | 2.712196497 | 1.504017756 |
| ENSMUSG00000081723 | 3.648161678 | 10.25447534 |
| ENSMUSG00000062524 | 1.983142671 | 2.032112722 |
| ENSMUSG00000004508 | 1.733603076 | 2.60608389 |
| ENSMUSG00000030589 | 2.020589018 | 3.150827469 |
| ENSMUSG00000037239 | 1.447245405 | 4.363863342 |
| ENSMUSG00000030798 | 2.565896901 | 4.901603235 |
| ENSMUSG00000053158 | 2.314268632 | 7.550928854 |
| ENSMUSG00000054342 | 3.202013054 | 7.666343373 |
| ENSMUSG00000004371 | 3.019836447 | 1.642161213 |
| ENSMUSG00000046223 | 6.054291443 | 14.15978558 |
| ENSMUSG00000130313 | 2.061685768 | 10.17589773 |
| ENSMUSG00000160211 | 3.079879127 | 19.55223116 |

Table S10 605 genes in down mode between the MOD and YMPH groups

| **rownames(res)** | **log_2_FoldChange** | **-log_10_FDA** |
| --- | --- | --- |
| ENSMUSG00000012187 | -2.381902738 | 1.947122394 |
| ENSMUSG00000032883 | -1.204997474 | 3.868146215 |
| ENSMUSG00000079470 | -1.366802632 | 2.586452803 |
| ENSMUSG00000101337 | -2.173213688 | 3.361254961 |
| ENSMUSG00000025986 | -2.635166436 | 4.825955908 |
| ENSMUSG00000102404 | -5.659081085 | 1.897021332 |
| ENSMUSG00000100502 | -5.503257867 | 1.540905293 |
| ENSMUSG00000096950 | -1.679666382 | 1.31310365 |
| ENSMUSG00000057715 | -3.135964727 | 1.558606956 |
| ENSMUSG00000114299 | -7.217069008 | 5.240349413 |
| ENSMUSG00000113880 | -4.131705369 | 31.36526507 |
| ENSMUSG00000113973 | -8.124365025 | 19.39687555 |
| ENSMUSG00000113846 | -8.703407787 | 13.30411971 |
| ENSMUSG00000113084 | -7.454039143 | 25.31521163 |
| ENSMUSG00000113925 | -9.186075968 | 14.67891392 |
| ENSMUSG00000114011 | -8.812177821 | 13.32839569 |
| ENSMUSG00000113097 | -10.95415689 | 15.86915214 |
| ENSMUSG00000038496 | -2.620949538 | 4.203575907 |
| ENSMUSG00000100190 | -9.223946727 | 25.38022709 |
| ENSMUSG00000113267 | -9.533161838 | 11.80268072 |
| ENSMUSG00000104423 | -11.40014668 | 16.94779606 |
| ENSMUSG00000101315 | -8.143962585 | 29.93691224 |
| ENSMUSG00000026380 | -1.926720125 | 4.890559893 |
| ENSMUSG00000055676 | -1.99559274 | 4.353956272 |
| ENSMUSG00000026383 | -1.64365808 | 2.191892551 |
| ENSMUSG00000092083 | -3.655895352 | 1.784713787 |
| ENSMUSG00000003721 | -1.387358453 | 3.801925715 |
| ENSMUSG00000101791 | -6.773395195 | 4.270181747 |
| ENSMUSG00000050534 | -3.458981028 | 3.900840131 |
| ENSMUSG00000026227 | -2.390148586 | 1.406671066 |
| ENSMUSG00000049690 | -1.538039942 | 1.355674524 |
| ENSMUSG00000100720 | -1.620435908 | 1.868385558 |
| ENSMUSG00000025927 | -3.217578235 | 4.225613885 |
| ENSMUSG00000102960 | -3.971294742 | 1.444563061 |
| ENSMUSG00000025931 | -1.409360463 | 1.739349328 |
| ENSMUSG00000026241 | -3.454668141 | 3.794575989 |
| ENSMUSG00000025934 | -2.236379673 | 1.657043299 |
| ENSMUSG00000004031 | -2.766209546 | 2.956791533 |
| ENSMUSG00000026621 | -2.440422986 | 1.305161923 |
| ENSMUSG00000015829 | -2.979596501 | 2.502727795 |
| ENSMUSG00000090394 | -1.447484206 | 2.718242917 |
| ENSMUSG00000016526 | -1.546593026 | 1.613069517 |
| ENSMUSG00000026258 | -6.556492294 | 2.698183656 |
| ENSMUSG00000047528 | -2.233491816 | 3.616857044 |
| ENSMUSG00000086727 | -3.614299011 | 2.34291332 |
| ENSMUSG00000037624 | -3.010250347 | 4.707183193 |
| ENSMUSG00000104507 | -3.744442199 | 2.514227275 |
| ENSMUSG00000026604 | -1.312158358 | 1.383999393 |
| ENSMUSG00000042251 | -3.008517314 | 11.98984095 |
| ENSMUSG00000044835 | -6.469263141 | 3.845986632 |
| ENSMUSG00000026435 | -3.19165614 | 5.202115034 |
| ENSMUSG00000042115 | -2.068531599 | 2.362303953 |
| ENSMUSG00000053024 | -2.875231086 | 2.942674995 |
| ENSMUSG00000026018 | -2.220341112 | 2.265422743 |
| ENSMUSG00000055833 | -2.01592058 | 3.136428735 |
| ENSMUSG00000037434 | -2.671336853 | 8.58800781 |
| ENSMUSG00000026443 | -2.959206123 | 4.631753453 |
| ENSMUSG00000058248 | -1.656390014 | 1.354626696 |
| ENSMUSG00000052062 | -1.538225251 | 2.892333214 |
| ENSMUSG00000026303 | -2.197998688 | 2.018964126 |
| ENSMUSG00000026640 | -2.194632164 | 5.848212581 |
| ENSMUSG00000100426 | -3.088912926 | 4.049450787 |
| ENSMUSG00000101265 | -4.194857359 | 1.820094294 |
| ENSMUSG00000045005 | -2.649904393 | 8.5815298 |
| ENSMUSG00000025955 | -3.887877897 | 4.052533479 |
| ENSMUSG00000040596 | -1.047593037 | 2.265422743 |
| ENSMUSG00000026090 | -2.574316079 | 2.940790853 |
| ENSMUSG00000055567 | -1.799010309 | 1.313810375 |
| ENSMUSG00000061584 | -7.060846263 | 7.801276572 |
| ENSMUSG00000040693 | -3.373689952 | 4.495102576 |
| ENSMUSG00000025991 | -3.376785342 | 2.16287035 |
| ENSMUSG00000097083 | -3.738341188 | 4.270181747 |
| ENSMUSG00000104145 | -1.416332543 | 1.832100528 |
| ENSMUSG00000098206 | -1.710219119 | 2.666624725 |
| ENSMUSG00000048775 | -3.952685365 | 4.258044418 |
| ENSMUSG00000026062 | -1.814986622 | 2.778716722 |
| ENSMUSG00000062588 | -1.819852075 | 2.41116606 |
| ENSMUSG00000086161 | -3.697364684 | 2.364613193 |
| ENSMUSG00000099576 | -5.917196807 | 2.842483149 |
| ENSMUSG00000026556 | -2.442737721 | 2.465767106 |
| ENSMUSG00000102278 | -3.202077709 | 1.51844468 |
| ENSMUSG00000049598 | -5.1356607 | 17.38024106 |
| ENSMUSG00000006411 | -2.242856365 | 3.033351336 |
| ENSMUSG00000038599 | -6.966993622 | 10.83612076 |
| ENSMUSG00000026205 | -3.912639405 | 6.699937275 |
| ENSMUSG00000041670 | -2.691194783 | 2.466509283 |
| ENSMUSG00000025993 | -1.851796167 | 1.842357576 |
| ENSMUSG00000087422 | -6.51010165 | 3.928515716 |
| ENSMUSG00000074682 | -1.023530059 | 1.501683731 |
| ENSMUSG00000009614 | -2.096023975 | 3.582849742 |
| ENSMUSG00000051396 | -1.226440277 | 2.54071042 |
| ENSMUSG00000034903 | -1.394128163 | 3.421100984 |
| ENSMUSG00000015647 | -1.258234463 | 1.743298139 |
| ENSMUSG00000038963 | -1.479233276 | 1.302111186 |
| ENSMUSG00000045319 | -1.981467402 | 2.809518078 |
| ENSMUSG00000039046 | -1.54072061 | 2.511854199 |
| ENSMUSG00000027577 | -2.967825825 | 2.20870309 |
| ENSMUSG00000015619 | -2.106752629 | 1.512936056 |
| ENSMUSG00000025783 | -2.449985142 | 1.348636064 |
| ENSMUSG00000074665 | -3.172847211 | 1.679936254 |
| ENSMUSG00000027070 | -2.494746458 | 1.60481388 |
| ENSMUSG00000000876 | -1.231873989 | 3.069472894 |
| ENSMUSG00000027605 | -1.312062428 | 6.945244044 |
| ENSMUSG00000027015 | -4.002914711 | 5.036979972 |
| ENSMUSG00000027579 | -2.566491755 | 2.055405299 |
| ENSMUSG00000013338 | -5.910293749 | 3.96908817 |
| ENSMUSG00000003418 | -2.727525403 | 6.624999255 |
| ENSMUSG00000041911 | -3.962578965 | 5.099067344 |
| ENSMUSG00000023391 | -3.898819102 | 5.05134712 |
| ENSMUSG00000086231 | -2.501592113 | 2.742122311 |
| ENSMUSG00000053702 | -2.27902416 | 1.544157479 |
| ENSMUSG00000038605 | -1.109934454 | 1.619001319 |
| ENSMUSG00000068452 | -1.629729894 | 1.72375122 |
| ENSMUSG00000039648 | -1.51859581 | 2.739387043 |
| ENSMUSG00000025716 | -5.383594933 | 1.619157687 |
| ENSMUSG00000035183 | -2.583155909 | 1.508093836 |
| ENSMUSG00000044349 | -1.520642806 | 1.712278213 |
| ENSMUSG00000044405 | -1.939483149 | 2.265422743 |
| ENSMUSG00000074625 | -2.268656554 | 1.798420393 |
| ENSMUSG00000046845 | -4.676454904 | 8.770612565 |
| ENSMUSG00000040434 | -2.431888205 | 2.083908808 |
| ENSMUSG00000044916 | -1.445125741 | 3.144875708 |
| ENSMUSG00000027412 | -1.132862926 | 1.746531971 |
| ENSMUSG00000086152 | -5.215668063 | 2.008606631 |
| ENSMUSG00000075020 | -2.460228674 | 1.361675038 |
| ENSMUSG00000075012 | -1.181203173 | 1.375432875 |
| ENSMUSG00000027380 | -2.825872526 | 4.540708694 |
| ENSMUSG00000027186 | -5.839470678 | 17.55380223 |
| ENSMUSG00000036813 | -8.202079834 | 6.700018209 |
| ENSMUSG00000017897 | -2.887741 | 5.550716194 |
| ENSMUSG00000037727 | -8.349796448 | 7.743425759 |
| ENSMUSG00000027171 | -1.859858169 | 1.548793233 |
| ENSMUSG00000087617 | -4.767288094 | 4.087314705 |
| ENSMUSG00000015093 | -2.044489348 | 1.744374258 |
| ENSMUSG00000015090 | -3.384188585 | 2.179227222 |
| ENSMUSG00000027329 | -1.68422616 | 2.970609071 |
| ENSMUSG00000026943 | -4.974738111 | 2.104965148 |
| ENSMUSG00000074981 | -4.315746968 | 1.976435309 |
| ENSMUSG00000044641 | -1.703683458 | 2.305181367 |
| ENSMUSG00000050199 | -1.688430126 | 2.149683156 |
| ENSMUSG00000050808 | -3.122580567 | 4.502869959 |
| ENSMUSG00000027134 | -1.754705604 | 2.827018717 |
| ENSMUSG00000074939 | -4.058060346 | 1.448685978 |
| ENSMUSG00000013523 | -3.045193365 | 6.376896783 |
| ENSMUSG00000026870 | -2.001978542 | 1.381055377 |
| ENSMUSG00000008999 | -1.801978408 | 1.513040916 |
| ENSMUSG00000027356 | -1.638766776 | 1.830751579 |
| ENSMUSG00000087382 | -1.830308746 | 1.730996132 |
| ENSMUSG00000074771 | -2.18445998 | 1.775764791 |
| ENSMUSG00000086214 | -5.674671785 | 5.833961902 |
| ENSMUSG00000085071 | -2.194566968 | 1.968524108 |
| ENSMUSG00000039092 | -1.843052648 | 1.449044143 |
| ENSMUSG00000074766 | -1.936728939 | 3.128234488 |
| ENSMUSG00000051379 | -2.255401115 | 3.43426764 |
| ENSMUSG00000027419 | -3.238700556 | 1.81236597 |
| ENSMUSG00000026923 | -2.164815717 | 3.319628329 |
| ENSMUSG00000063873 | -2.069609044 | 3.285233545 |
| ENSMUSG00000078137 | -3.48113379 | 2.742122311 |
| ENSMUSG00000026915 | -1.3900469 | 2.327734433 |
| ENSMUSG00000027324 | -1.182893276 | 2.386227999 |
| ENSMUSG00000000247 | -2.718726176 | 2.763633477 |
| ENSMUSG00000055926 | -2.340462748 | 7.566934303 |
| ENSMUSG00000068115 | -1.703193378 | 2.486355029 |
| ENSMUSG00000027315 | -2.577499195 | 3.138847783 |
| ENSMUSG00000026765 | -2.025165117 | 1.367749727 |
| ENSMUSG00000050447 | -2.487560983 | 5.444482899 |
| ENSMUSG00000017144 | -1.017860618 | 1.707940103 |
| ENSMUSG00000085014 | -1.803473797 | 1.904724416 |
| ENSMUSG00000083287 | -1.738397376 | 1.619764181 |
| ENSMUSG00000026989 | -5.401044374 | 20.68921266 |
| ENSMUSG00000040794 | -1.304910813 | 2.380387919 |
| ENSMUSG00000027403 | -6.492644702 | 7.390694281 |
| ENSMUSG00000057836 | -1.814397633 | 1.328160896 |
| ENSMUSG00000058147 | -1.649077723 | 1.418426602 |
| ENSMUSG00000025265 | -1.023950017 | 1.393625672 |
| ENSMUSG00000067377 | -1.384016786 | 1.597203779 |
| ENSMUSG00000087403 | -1.007666286 | 1.42637285 |
| ENSMUSG00000031258 | -3.017393491 | 4.614038628 |
| ENSMUSG00000025333 | -3.929696672 | 2.758569627 |
| ENSMUSG00000033578 | -2.033631107 | 1.749063727 |
| ENSMUSG00000073295 | -1.918122937 | 5.613932468 |
| ENSMUSG00000073293 | -2.471845981 | 5.903753199 |
| ENSMUSG00000083316 | -5.404896334 | 1.902510169 |
| ENSMUSG00000031410 | -4.840163618 | 3.073808595 |
| ENSMUSG00000031112 | -1.295969996 | 1.440148497 |
| ENSMUSG00000036131 | -2.877632656 | 5.100278207 |
| ENSMUSG00000048355 | -1.583496837 | 1.335783626 |
| ENSMUSG00000047844 | -2.43427541 | 1.456412188 |
| ENSMUSG00000087644 | -1.748390618 | 2.558791443 |
| ENSMUSG00000044550 | -2.255955214 | 2.72210159 |
| ENSMUSG00000035967 | -1.138821776 | 1.433028317 |
| ENSMUSG00000031398 | -1.634650081 | 1.423199228 |
| ENSMUSG00000031298 | -1.740774322 | 5.215557099 |
| ENSMUSG00000031150 | -1.665064861 | 1.890188215 |
| ENSMUSG00000031292 | -2.408768201 | 4.06701648 |
| ENSMUSG00000031273 | -3.734848179 | 3.049284483 |
| ENSMUSG00000059493 | -2.344021709 | 2.171757832 |
| ENSMUSG00000031274 | -2.030508571 | 2.605294091 |
| ENSMUSG00000015405 | -3.718981463 | 2.641046352 |
| ENSMUSG00000031284 | -2.5797245 | 3.40163499 |
| ENSMUSG00000039201 | -1.14823572 | 1.635593693 |
| ENSMUSG00000081738 | -1.161113788 | 1.513437855 |
| ENSMUSG00000015217 | -1.397813341 | 1.329177737 |
| ENSMUSG00000000402 | -2.949998319 | 2.113172305 |
| ENSMUSG00000084920 | -5.228007318 | 5.543878469 |
| ENSMUSG00000101819 | -4.458855611 | 2.19832381 |
| ENSMUSG00000031217 | -2.162516384 | 2.605294091 |
| ENSMUSG00000071719 | -2.38986492 | 3.840983058 |
| ENSMUSG00000031220 | -2.147961895 | 3.071431151 |
| ENSMUSG00000015665 | -1.569244729 | 1.422509653 |
| ENSMUSG00000087586 | -4.379957282 | 1.971905921 |
| ENSMUSG00000051159 | -2.350364676 | 1.424760581 |
| ENSMUSG00000085715 | -6.003315993 | 3.177385335 |
| ENSMUSG00000082746 | -4.518797564 | 1.978611211 |
| ENSMUSG00000043461 | -4.508070496 | 25.62656225 |
| ENSMUSG00000068876 | -4.136621837 | 5.904913053 |
| ENSMUSG00000106073 | -1.595789166 | 1.809865214 |
| ENSMUSG00000028009 | -3.895073572 | 2.626992615 |
| ENSMUSG00000034109 | -1.166557652 | 1.820126964 |
| ENSMUSG00000039865 | -2.017110422 | 5.465638952 |
| ENSMUSG00000027710 | -1.268321023 | 1.626152747 |
| ENSMUSG00000105419 | -1.491545426 | 1.775764791 |
| ENSMUSG00000058400 | -7.050077155 | 5.079761687 |
| ENSMUSG00000028125 | -1.627915141 | 2.456973024 |
| ENSMUSG00000033900 | -1.548059081 | 6.290921205 |
| ENSMUSG00000039735 | -1.643453708 | 1.676482852 |
| ENSMUSG00000068860 | -2.937420032 | 5.02333935 |
| ENSMUSG00000028115 | -2.215227036 | 1.617716683 |
| ENSMUSG00000015702 | -3.210640816 | 5.355417445 |
| ENSMUSG00000027993 | -2.501035061 | 4.021179093 |
| ENSMUSG00000104043 | -2.114102983 | 2.237464138 |
| ENSMUSG00000041842 | -2.950004769 | 5.403858536 |
| ENSMUSG00000110441 | -3.075036394 | 3.449171261 |
| ENSMUSG00000028179 | -1.373204016 | 2.738447352 |
| ENSMUSG00000097639 | -6.57409798 | 2.726219685 |
| ENSMUSG00000104616 | -7.529845538 | 4.077117718 |
| ENSMUSG00000106223 | -5.038545788 | 1.403957864 |
| ENSMUSG00000028076 | -1.072980846 | 1.30134554 |
| ENSMUSG00000003382 | -1.037487404 | 1.435168915 |
| ENSMUSG00000095836 | -3.218699641 | 3.243861881 |
| ENSMUSG00000105837 | -4.580218263 | 3.403944904 |
| ENSMUSG00000041220 | -3.161007283 | 4.094305189 |
| ENSMUSG00000028100 | -2.604048914 | 5.732085472 |
| ENSMUSG00000104109 | -4.079127787 | 2.719368198 |
| ENSMUSG00000038298 | -3.897720712 | 5.157254829 |
| ENSMUSG00000027985 | -3.29088383 | 3.119856281 |
| ENSMUSG00000028093 | -1.27729969 | 2.640513927 |
| ENSMUSG00000027875 | -2.423590204 | 1.987949551 |
| ENSMUSG00000027869 | -2.839514382 | 2.58812106 |
| ENSMUSG00000074489 | -2.010331713 | 2.892083255 |
| ENSMUSG00000044365 | -2.398472369 | 1.749063727 |
| ENSMUSG00000027796 | -2.110121893 | 3.804867871 |
| ENSMUSG00000051076 | -5.263755756 | 7.151646066 |
| ENSMUSG00000104737 | -1.439716587 | 1.418836302 |
| ENSMUSG00000074480 | -2.285697722 | 2.486355029 |
| ENSMUSG00000040209 | -1.090763847 | 3.195137748 |
| ENSMUSG00000027528 | -4.36631054 | 2.097625551 |
| ENSMUSG00000053897 | -2.256425585 | 2.303870549 |
| ENSMUSG00000106290 | -2.427438093 | 2.892333214 |
| ENSMUSG00000105681 | -2.494632511 | 1.842357576 |
| ENSMUSG00000027536 | -2.272364555 | 4.026502037 |
| ENSMUSG00000027800 | -1.628308581 | 4.890559893 |
| ENSMUSG00000027801 | -5.434170776 | 4.822593886 |
| ENSMUSG00000097339 | -3.072671282 | 2.272694985 |
| ENSMUSG00000105003 | -3.331651384 | 3.928870036 |
| ENSMUSG00000053054 | -4.400314201 | 9.106421654 |
| ENSMUSG00000074206 | -4.649498524 | 12.67705631 |
| ENSMUSG00000098008 | -7.079304542 | 4.010906884 |
| ENSMUSG00000090527 | -2.34899299 | 3.252894361 |
| ENSMUSG00000074589 | -2.473492529 | 1.842627431 |
| ENSMUSG00000036951 | -2.918088594 | 2.262123038 |
| ENSMUSG00000036885 | -2.525289747 | 3.083993761 |
| ENSMUSG00000102758 | -1.880365479 | 3.27954919 |
| ENSMUSG00000036834 | -1.902494671 | 2.104230113 |
| ENSMUSG00000028266 | -1.355271862 | 5.108248539 |
| ENSMUSG00000104969 | -2.176720353 | 3.239530413 |
| ENSMUSG00000028039 | -3.107848185 | 3.528553371 |
| ENSMUSG00000028040 | -2.089882938 | 1.657043299 |
| ENSMUSG00000049404 | -3.017707871 | 4.173915556 |
| ENSMUSG00000036863 | -1.812146335 | 2.588494537 |
| ENSMUSG00000036832 | -2.032885948 | 1.740677528 |
| ENSMUSG00000086968 | -1.442659968 | 1.447452687 |
| ENSMUSG00000027692 | -1.004011758 | 3.082435762 |
| ENSMUSG00000068747 | -1.446693151 | 2.760520086 |
| ENSMUSG00000068744 | -2.056057738 | 1.347451875 |
| ENSMUSG00000068740 | -2.437304159 | 2.956410259 |
| ENSMUSG00000040412 | -3.586710795 | 4.890559893 |
| ENSMUSG00000040389 | -1.719540766 | 2.685525519 |
| ENSMUSG00000037643 | -1.39996261 | 1.431701888 |
| ENSMUSG00000105509 | -1.058017973 | 1.375432875 |
| ENSMUSG00000001021 | -5.467112637 | 8.04958905 |
| ENSMUSG00000056270 | -5.601390345 | 6.600335242 |
| ENSMUSG00000118547 | -2.628171962 | 2.444556545 |
| ENSMUSG00000045566 | -5.409805885 | 7.334671861 |
| ENSMUSG00000078657 | -5.771399714 | 7.443116001 |
| ENSMUSG00002075117 | -4.848000955 | 1.305921927 |
| ENSMUSG00000057829 | -9.080017996 | 9.598399477 |
| ENSMUSG00000052415 | -5.60340894 | 11.38673738 |
| ENSMUSG00000027908 | -7.422104761 | 31.36526507 |
| ENSMUSG00000028145 | -1.348189895 | 3.233207489 |
| ENSMUSG00000070583 | -1.137867377 | 2.404317956 |
| ENSMUSG00000025418 | -4.113283298 | 1.412090172 |
| ENSMUSG00000085933 | -4.477270154 | 5.602892594 |
| ENSMUSG00000034926 | -3.728668273 | 6.274777193 |
| ENSMUSG00000048485 | -2.743256443 | 2.322490973 |
| ENSMUSG00000047502 | -1.657324052 | 1.479403459 |
| ENSMUSG00000034785 | -5.645048274 | 1.766835998 |
| ENSMUSG00000073830 | -3.596635008 | 2.329318272 |
| ENSMUSG00000087273 | -2.334735746 | 2.239875775 |
| ENSMUSG00000028977 | -1.318085221 | 2.20870309 |
| ENSMUSG00000028391 | -1.608861853 | 1.868044173 |
| ENSMUSG00000028392 | -2.929048548 | 2.388856144 |
| ENSMUSG00000038422 | -1.14691852 | 1.513040916 |
| ENSMUSG00000028393 | -1.376255886 | 4.70319108 |
| ENSMUSG00000028358 | -2.28159066 | 2.800804845 |
| ENSMUSG00000028601 | -1.11853063 | 2.046337608 |
| ENSMUSG00000034645 | -1.823422836 | 1.310736354 |
| ENSMUSG00000028909 | -2.202901055 | 2.805895052 |
| ENSMUSG00000039137 | -1.816713419 | 1.328974292 |
| ENSMUSG00000028972 | -8.306760448 | 21.4044672 |
| ENSMUSG00000046341 | -2.201609852 | 1.592925629 |
| ENSMUSG00000102796 | -4.152883221 | 2.653143244 |
| ENSMUSG00000043003 | -3.330665721 | 2.104230113 |
| ENSMUSG00000066042 | -1.049959275 | 1.642500835 |
| ENSMUSG00000028885 | -1.391886632 | 2.241884176 |
| ENSMUSG00000028940 | -3.286402368 | 1.920496435 |
| ENSMUSG00000028865 | -2.897068081 | 2.609083836 |
| ENSMUSG00000028862 | -1.432705302 | 1.702572342 |
| ENSMUSG00000028860 | -2.309840636 | 2.108552664 |
| ENSMUSG00000048706 | -2.0440121 | 2.617815806 |
| ENSMUSG00000046694 | -1.644900991 | 2.306972248 |
| ENSMUSG00000070867 | -1.849856841 | 4.839731792 |
| ENSMUSG00000039577 | -1.007803778 | 1.347992781 |
| ENSMUSG00000039492 | -2.291641258 | 3.197156639 |
| ENSMUSG00000085022 | -2.140159672 | 2.940790853 |
| ENSMUSG00000034171 | -1.937175399 | 2.836501615 |
| ENSMUSG00000048001 | -5.257309173 | 1.823508336 |
| ENSMUSG00000037348 | -3.043507192 | 5.460823111 |
| ENSMUSG00000028699 | -3.683692397 | 2.97438872 |
| ENSMUSG00000028487 | -2.622026637 | 3.306470962 |
| ENSMUSG00000029055 | -1.662752954 | 1.669148404 |
| ENSMUSG00000028803 | -1.213437639 | 2.243621497 |
| ENSMUSG00000062157 | -1.554252371 | 1.971592003 |
| ENSMUSG00000018983 | -1.656767996 | 1.702754875 |
| ENSMUSG00000117123 | -2.343949728 | 2.457645449 |
| ENSMUSG00000085569 | -3.488072636 | 3.238605479 |
| ENSMUSG00000028681 | -2.325140974 | 2.113583349 |
| ENSMUSG00000070687 | -4.738925614 | 6.828247997 |
| ENSMUSG00000001089 | -1.206258614 | 2.032385709 |
| ENSMUSG00000078487 | -2.909586287 | 3.847750129 |
| ENSMUSG00000028572 | -1.020431222 | 1.932543443 |
| ENSMUSG00000081225 | -2.543242479 | 2.116972034 |
| ENSMUSG00000082932 | -3.188600647 | 3.913038353 |
| ENSMUSG00000023571 | -1.264639344 | 1.727614266 |
| ENSMUSG00000029074 | -2.143670544 | 2.099319249 |
| ENSMUSG00000086549 | -2.632509606 | 2.612635935 |
| ENSMUSG00000033295 | -1.805612928 | 1.888645595 |
| ENSMUSG00000028730 | -2.741762807 | 3.809816012 |
| ENSMUSG00000061859 | -1.62271364 | 2.19832381 |
| ENSMUSG00000028751 | -2.881801962 | 5.135841015 |
| ENSMUSG00000035275 | -1.936391777 | 2.2303264 |
| ENSMUSG00000070803 | -3.175230339 | 5.673996916 |
| ENSMUSG00000028528 | -2.300266748 | 3.538225474 |
| ENSMUSG00000078234 | -2.470867954 | 3.083993761 |
| ENSMUSG00000028655 | -2.242438499 | 1.426553759 |
| ENSMUSG00000028654 | -3.127992676 | 2.185307226 |
| ENSMUSG00000025330 | -2.667703024 | 5.443798093 |
| ENSMUSG00000025328 | -4.679734578 | 4.289977094 |
| ENSMUSG00000032744 | -1.87320069 | 1.883107834 |
| ENSMUSG00000085918 | -4.647198971 | 3.252894361 |
| ENSMUSG00000025329 | -4.955024875 | 10.43931308 |
| ENSMUSG00000032726 | -1.82301749 | 2.128125633 |
| ENSMUSG00000073733 | -1.610403119 | 2.35353715 |
| ENSMUSG00000006445 | -1.881293121 | 2.743726536 |
| ENSMUSG00000006218 | -3.434523318 | 3.347524446 |
| ENSMUSG00000028591 | -2.863405425 | 1.848874302 |
| ENSMUSG00000028589 | -5.048234857 | 1.35898195 |
| ENSMUSG00000095213 | -2.984064975 | 3.281787917 |
| ENSMUSG00000078507 | -3.015804753 | 3.83694158 |
| ENSMUSG00000041735 | -3.294840332 | 9.316945155 |
| ENSMUSG00000078504 | -1.795107638 | 1.656943077 |
| ENSMUSG00000098760 | -1.962220372 | 1.474633579 |
| ENSMUSG00000001985 | -2.369083583 | 3.903935798 |
| ENSMUSG00000042616 | -1.278099375 | 2.116081472 |
| ENSMUSG00000073758 | -1.464185782 | 1.478253273 |
| ENSMUSG00000042367 | -2.01474586 | 3.063697162 |
| ENSMUSG00000046623 | -1.693926404 | 2.292619099 |
| ENSMUSG00000028785 | -2.083044971 | 1.45091423 |
| ENSMUSG00000040606 | -1.290891011 | 1.548991134 |
| ENSMUSG00000055296 | -1.050777932 | 2.498926599 |
| ENSMUSG00000028434 | -1.787732415 | 3.076283545 |
| ENSMUSG00000071019 | -2.036725491 | 1.94730369 |
| ENSMUSG00000086127 | -2.878560446 | 1.507796811 |
| ENSMUSG00000028386 | -2.771441043 | 4.561940813 |
| ENSMUSG00000085558 | -2.668791112 | 5.105697685 |
| ENSMUSG00000028444 | -2.638294762 | 3.252620963 |
| ENSMUSG00000040536 | -4.067024512 | 7.758689952 |
| ENSMUSG00000043252 | -1.646403059 | 5.986021292 |
| ENSMUSG00000073888 | -2.204954032 | 2.757034531 |
| ENSMUSG00000073988 | -1.49473438 | 2.585912377 |
| ENSMUSG00000078735 | -5.000444759 | 9.817878097 |
| ENSMUSG00000102504 | -1.83546136 | 3.051793503 |
| ENSMUSG00000087383 | -4.634035665 | 4.715558281 |
| ENSMUSG00000070990 | -3.872149017 | 4.316497874 |
| ENSMUSG00000028347 | -1.87142979 | 2.278030654 |
| ENSMUSG00000086267 | -3.609898089 | 2.308668861 |
| ENSMUSG00000084970 | -2.220601565 | 2.502727795 |
| ENSMUSG00000040706 | -1.534862908 | 1.42340486 |
| ENSMUSG00000105345 | -1.580182243 | 2.588494537 |
| ENSMUSG00000093910 | -2.474624634 | 1.583229829 |
| ENSMUSG00000038569 | -1.121201715 | 1.358010333 |
| ENSMUSG00000001467 | -1.358749968 | 2.20596469 |
| ENSMUSG00000044674 | -1.693904487 | 1.97565665 |
| ENSMUSG00000029211 | -2.789563465 | 1.757766369 |
| ENSMUSG00000104684 | -2.1744697 | 4.077867473 |
| ENSMUSG00000034573 | -2.107011174 | 3.057153324 |
| ENSMUSG00000029320 | -3.266148859 | 2.983361804 |
| ENSMUSG00000005220 | -2.246290884 | 1.400259097 |
| ENSMUSG00000054252 | -2.699922747 | 3.471504922 |
| ENSMUSG00000070419 | -2.757768945 | 5.443289629 |
| ENSMUSG00000038656 | -3.548863998 | 1.577205276 |
| ENSMUSG00000106943 | -1.231288816 | 1.822031724 |
| ENSMUSG00000049907 | -1.927843901 | 2.812249832 |
| ENSMUSG00000054434 | -1.286248157 | 3.122879435 |
| ENSMUSG00000029438 | -1.644199339 | 2.993489217 |
| ENSMUSG00000003623 | -1.10727347 | 2.033398239 |
| ENSMUSG00000029641 | -2.432204403 | 5.267104154 |
| ENSMUSG00000005672 | -1.513468771 | 1.885633372 |
| ENSMUSG00000085971 | -1.651309843 | 2.974374594 |
| ENSMUSG00000063531 | -2.141158539 | 4.702318404 |
| ENSMUSG00000059325 | -2.076691765 | 3.813789537 |
| ENSMUSG00000087541 | -1.95203993 | 2.465767106 |
| ENSMUSG00000061601 | -2.225572289 | 6.624999255 |
| ENSMUSG00000029503 | -3.478672951 | 1.940588026 |
| ENSMUSG00000043430 | -2.159346232 | 3.082435762 |
| ENSMUSG00000037979 | -2.042523982 | 2.10278336 |
| ENSMUSG00000079215 | -1.324027722 | 3.278965916 |
| ENSMUSG00000066975 | -4.331872957 | 17.91677656 |
| ENSMUSG00000048450 | -2.669752454 | 2.314642882 |
| ENSMUSG00000105039 | -3.274937126 | 2.175634715 |
| ENSMUSG00000029482 | -1.353926141 | 3.493260046 |
| ENSMUSG00000063430 | -1.767467421 | 1.350409615 |
| ENSMUSG00000104980 | -6.448420001 | 3.433335262 |
| ENSMUSG00000072591 | -1.907623036 | 2.28982596 |
| ENSMUSG00000105059 | -2.874293386 | 2.603158759 |
| ENSMUSG00000081683 | -2.514248818 | 1.838338381 |
| ENSMUSG00000054537 | -2.458196635 | 1.384067703 |
| ENSMUSG00000104701 | -1.919258848 | 1.398576971 |
| ENSMUSG00000009580 | -8.412862918 | 7.825925142 |
| ENSMUSG00000001622 | -6.237610211 | 14.41804144 |
| ENSMUSG00000029550 | -1.080700566 | 1.376999618 |
| ENSMUSG00000039682 | -1.685766965 | 3.95810735 |
| ENSMUSG00000054256 | -2.667029247 | 5.836671296 |
| ENSMUSG00000029370 | -3.350971176 | 4.192433989 |
| ENSMUSG00000029090 | -2.698477335 | 5.199782914 |
| ENSMUSG00000106648 | -4.94852128 | 1.452975893 |
| ENSMUSG00000043913 | -2.744138191 | 1.73845287 |
| ENSMUSG00000029130 | -2.001297569 | 2.580305365 |
| ENSMUSG00000057068 | -4.12722594 | 2.452072477 |
| ENSMUSG00000050050 | -2.361957863 | 2.219198346 |
| ENSMUSG00000105940 | -2.422668026 | 2.570238599 |
| ENSMUSG00000029381 | -1.758246207 | 1.976435309 |
| ENSMUSG00000045314 | -3.741309869 | 3.796772317 |
| ENSMUSG00000037999 | -1.54379285 | 1.325507394 |
| ENSMUSG00000105694 | -3.020418269 | 1.3813442 |
| ENSMUSG00000105707 | -2.997034759 | 2.953215774 |
| ENSMUSG00000029168 | -2.101897885 | 1.476222109 |
| ENSMUSG00000055725 | -2.178106993 | 2.943150625 |
| ENSMUSG00000029337 | -5.75214055 | 19.18746075 |
| ENSMUSG00000057816 | -2.322571504 | 1.843264632 |
| ENSMUSG00000013629 | -1.19885011 | 1.467079887 |
| ENSMUSG00000029151 | -2.172094055 | 1.626072282 |
| ENSMUSG00000029134 | -3.330811498 | 9.865275784 |
| ENSMUSG00000047501 | -3.292070361 | 7.195694699 |
| ENSMUSG00000070473 | -5.636964152 | 17.68661239 |
| ENSMUSG00000029705 | -1.790315407 | 4.100554041 |
| ENSMUSG00000036687 | -2.119524943 | 3.840379874 |
| ENSMUSG00000046658 | -1.185459935 | 3.149984821 |
| ENSMUSG00000060261 | -1.109360454 | 1.864101989 |
| ENSMUSG00000107076 | -3.497670937 | 1.775764791 |
| ENSMUSG00000023079 | -1.758519694 | 2.563773978 |
| ENSMUSG00000108292 | -4.256473771 | 1.823682304 |
| ENSMUSG00000030235 | -2.958942639 | 2.938641311 |
| ENSMUSG00000029868 | -4.593072256 | 4.657556777 |
| ENSMUSG00000063975 | -2.371206344 | 2.865963086 |
| ENSMUSG00000044927 | -2.255970904 | 1.535326692 |
| ENSMUSG00000084950 | -2.122800276 | 2.293912055 |
| ENSMUSG00000012396 | -4.043125549 | 1.431985155 |
| ENSMUSG00000029859 | -2.474327304 | 1.846468629 |
| ENSMUSG00000033174 | -1.766127217 | 3.963349634 |
| ENSMUSG00000008153 | -2.457333339 | 3.192080793 |
| ENSMUSG00000030084 | -1.286787685 | 1.428520229 |
| ENSMUSG00000107570 | -3.006068139 | 1.947122394 |
| ENSMUSG00000025821 | -1.275316288 | 1.659080782 |
| ENSMUSG00000087327 | -1.910812693 | 1.92415798 |
| ENSMUSG00000099609 | -2.031734769 | 2.574969804 |
| ENSMUSG00000041540 | -1.58484373 | 2.220153885 |
| ENSMUSG00000086013 | -1.260522616 | 1.327926397 |
| ENSMUSG00000047115 | -1.861456724 | 5.836471637 |
| ENSMUSG00000029819 | -1.937802436 | 1.498571698 |
| ENSMUSG00000030020 | -1.840984012 | 4.464290667 |
| ENSMUSG00000048776 | -3.436589499 | 4.755387178 |
| ENSMUSG00000108236 | -4.007352493 | 1.338923734 |
| ENSMUSG00000030303 | -2.625063136 | 4.072933217 |
| ENSMUSG00000029832 | -3.543653531 | 5.747516209 |
| ENSMUSG00000030029 | -1.019523832 | 1.758339043 |
| ENSMUSG00000030031 | -1.000873521 | 1.327147009 |
| ENSMUSG00000107502 | -2.367840418 | 1.526677688 |
| ENSMUSG00000000184 | -1.792199143 | 2.421459728 |
| ENSMUSG00000039985 | -1.606454038 | 1.652052333 |
| ENSMUSG00000053007 | -1.48134366 | 3.721042741 |
| ENSMUSG00000034832 | -1.104521201 | 1.612513889 |
| ENSMUSG00000004633 | -2.319121254 | 6.147929761 |
| ENSMUSG00000041301 | -3.452569043 | 1.96917083 |
| ENSMUSG00000000416 | -1.839488155 | 1.403957864 |
| ENSMUSG00000106944 | -2.233983888 | 1.739076115 |
| ENSMUSG00000037973 | -4.875497102 | 2.661201464 |
| ENSMUSG00000035357 | -2.003533028 | 5.310282266 |
| ENSMUSG00000097834 | -3.850851239 | 1.775764791 |
| ENSMUSG00000107666 | -4.402008964 | 2.095711291 |
| ENSMUSG00000030200 | -1.892820209 | 1.479403459 |
| ENSMUSG00000030205 | -7.329813078 | 33.69043874 |
| ENSMUSG00000048022 | -2.701977826 | 7.929715674 |
| ENSMUSG00000039904 | -3.423952232 | 3.585375198 |
| ENSMUSG00000045441 | -2.553972996 | 2.648274265 |
| ENSMUSG00000046764 | -2.157249495 | 1.967714055 |
| ENSMUSG00000087341 | -2.54942334 | 3.149629584 |
| ENSMUSG00000097924 | -2.400086831 | 5.081488492 |
| ENSMUSG00000039578 | -1.992567144 | 2.144259974 |
| ENSMUSG00000056755 | -4.938425617 | 1.443727256 |
| ENSMUSG00000049112 | -3.021326064 | 5.935607318 |
| ENSMUSG00000059201 | -2.005685301 | 1.998848164 |
| ENSMUSG00000107653 | -4.266878114 | 6.486502534 |
| ENSMUSG00000030222 | -2.315541112 | 1.355946845 |
| ENSMUSG00000097603 | -1.915027369 | 1.441359329 |
| ENSMUSG00000030278 | -1.867838539 | 1.501683731 |
| ENSMUSG00000001763 | -1.376004272 | 2.112518975 |
| ENSMUSG00000068011 | -2.74620587 | 4.902602129 |
| ENSMUSG00000059878 | -1.030232769 | 2.602310338 |
| ENSMUSG00000042097 | -1.121011708 | 1.423058913 |
| ENSMUSG00000004988 | -3.831014527 | 3.448747728 |
| ENSMUSG00000030110 | -1.98790652 | 6.144189667 |
| ENSMUSG00000063455 | -1.436717176 | 1.328160896 |
| ENSMUSG00000097484 | -3.750272881 | 1.735654886 |
| ENSMUSG00000108282 | -4.811917969 | 1.316204636 |
| ENSMUSG00000041372 | -1.863416458 | 1.66078133 |
| ENSMUSG00000051586 | -1.032044057 | 1.384067703 |
| ENSMUSG00000067825 | -1.577889875 | 2.116972034 |
| ENSMUSG00000030228 | -2.404551034 | 2.952532653 |
| ENSMUSG00000029638 | -1.819868294 | 1.636263712 |
| ENSMUSG00000001157 | -1.098335953 | 1.524566394 |
| ENSMUSG00000037519 | -1.185679148 | 1.917692429 |
| ENSMUSG00000025104 | -1.004723308 | 2.215940244 |
| ENSMUSG00000025105 | -1.761453688 | 1.951062984 |
| ENSMUSG00000040703 | -2.616463487 | 2.793304019 |
| ENSMUSG00000108606 | -5.226993216 | 3.519110671 |
| ENSMUSG00000108413 | -4.044718627 | 5.077431524 |
| ENSMUSG00000040650 | -4.579160667 | 1.736218573 |
| ENSMUSG00000030523 | -3.802872943 | 3.728962064 |
| ENSMUSG00000030713 | -2.177333956 | 2.012410676 |
| ENSMUSG00000066704 | -3.887509891 | 4.625750384 |
| ENSMUSG00000055193 | -6.41896841 | 3.000688882 |
| ENSMUSG00000004651 | -2.762209839 | 1.393915953 |
| ENSMUSG00000062542 | -1.62570846 | 2.447227879 |
| ENSMUSG00000057093 | -1.080039462 | 1.730996132 |
| ENSMUSG00000109143 | -3.419339178 | 5.801902461 |
| ENSMUSG00000078779 | -1.231549528 | 2.022656035 |
| ENSMUSG00000036528 | -1.703127383 | 3.721042741 |
| ENSMUSG00000097455 | -6.156774741 | 4.451191479 |
| ENSMUSG00000053228 | -4.027749765 | 3.30334492 |
| ENSMUSG00000047730 | -3.734034005 | 8.996678933 |
| ENSMUSG00000045598 | -1.460280085 | 3.186061642 |
| ENSMUSG00000030739 | -2.310073116 | 6.221055185 |
| ENSMUSG00000054005 | -2.117298238 | 2.22589774 |
| ENSMUSG00000048012 | -1.311398804 | 1.508906135 |
| ENSMUSG00000053025 | -2.314834379 | 1.814233822 |
| ENSMUSG00000054753 | -6.7280516 | 16.09121426 |
| ENSMUSG00000034825 | -1.330770088 | 1.778372233 |
| ENSMUSG00000030602 | -1.416952436 | 1.739416723 |
| ENSMUSG00000007279 | -3.125940724 | 13.63681228 |
| ENSMUSG00000040734 | -1.711591294 | 1.406671066 |
| ENSMUSG00000044139 | -2.589886335 | 3.953990006 |
| ENSMUSG00000030616 | -2.270838326 | 3.868856318 |
| ENSMUSG00000053964 | -1.016859223 | 1.384716838 |
| ENSMUSG00000040714 | -1.960773672 | 1.587999072 |
| ENSMUSG00000054083 | -4.625767498 | 10.62122152 |
| ENSMUSG00000030800 | -2.151176322 | 1.971905921 |
| ENSMUSG00000011267 | -1.461219161 | 2.152667332 |
| ENSMUSG00000038296 | -1.022564711 | 1.671473572 |
| ENSMUSG00000050382 | -1.7840935 | 2.483049649 |
| ENSMUSG00000109395 | -5.250901956 | 1.645430353 |
| ENSMUSG00000030546 | -1.915115398 | 2.568110467 |
| ENSMUSG00000030545 | -1.162376539 | 2.167990781 |
| ENSMUSG00000039099 | -1.842589235 | 2.562235252 |
| ENSMUSG00000038244 | -1.410370244 | 2.762335683 |
| ENSMUSG00000048078 | -1.877124061 | 2.265422743 |
| ENSMUSG00000095276 | -5.261570081 | 2.113436814 |
| ENSMUSG00000038292 | -3.00174859 | 4.270181747 |
| ENSMUSG00000074227 | -1.593160526 | 1.69192156 |
| ENSMUSG00000003863 | -2.0432071 | 1.599579824 |
| ENSMUSG00000003872 | -2.299890206 | 1.712278213 |
| ENSMUSG00000030666 | -2.732208416 | 1.548991134 |
| ENSMUSG00000011632 | -5.225397233 | 6.995111738 |
| ENSMUSG00000051768 | -1.09792478 | 1.739349328 |
| ENSMUSG00000074277 | -1.847110598 | 1.919082614 |

Table S11 KEGG enrichment results between the MOD and YMPH groups for transcriptomics

| **ID** | **Description** | **GeneRatio** | **BgRatio** | **p.adjust** | **Count** |
| --- | --- | --- | --- | --- | --- |
| hsa04060 | Cytokine-cytokine receptor interaction | 35/134 | 294/7914 | 1.71869910792941e-18 | 35 |
| hsa04061 | Viral protein interaction with cytokine and cytokine receptor | 21/134 | 100/7914 | 7.74548787684448e-16 | 21 |
| hsa05140 | Leishmaniasis | 17/134 | 76/7914 | 2.73198266394191e-13 | 17 |
| hsa05162 | Measles | 21/134 | 138/7914 | 3.8169807014488e-13 | 21 |
| hsa04640 | Hematopoietic cell lineage | 17/134 | 98/7914 | 1.4356608855943e-11 | 17 |
| hsa05144 | Malaria | 13/134 | 49/7914 | 2.46132761536812e-11 | 13 |
| hsa05152 | Tuberculosis | 21/134 | 179/7914 | 4.25779875359488e-11 | 21 |
| hsa04062 | Chemokine signaling pathway | 21/134 | 189/7914 | 1.08513194963192e-10 | 21 |
| hsa04380 | Osteoclast differentiation | 17/134 | 128/7914 | 6.44050325262756e-10 | 17 |
| hsa04514 | Cell adhesion molecules (CAMs) | 18/134 | 147/7914 | 6.44050325262756e-10 | 18 |
| hsa04620 | Toll-like receptor signaling pathway | 15/134 | 104/7914 | 2.67136599523061e-09 | 15 |
| hsa05323 | Rheumatoid arthritis | 14/134 | 93/7914 | 5.75938412045907e-09 | 14 |
| hsa04650 | Natural killer cell mediated cytotoxicity | 16/134 | 131/7914 | 6.85534913329307e-09 | 16 |
| hsa05321 | Inflammatory bowel disease (IBD) | 12/134 | 65/7914 | 8.19988195919828e-09 | 12 |
| hsa05169 | Epstein-Barr virus infection | 19/134 | 201/7914 | 1.0948824319469e-08 | 19 |
| hsa04064 | NF-kappa B signaling pathway | 14/134 | 102/7914 | 1.52198080877206e-08 | 14 |
| hsa04668 | TNF signaling pathway | 14/134 | 112/7914 | 5.01483470424589e-08 | 14 |
| hsa05340 | Primary immunodeficiency | 9/134 | 38/7914 | 9.44748929423259e-08 | 9 |
| hsa04933 | AGE-RAGE signaling pathway in diabetic complications | 13/134 | 100/7914 | 1.01200841514004e-07 | 13 |
| hsa05164 | Influenza A | 16/134 | 170/7914 | 2.07031926037087e-07 | 16 |
| hsa04659 | Th17 cell differentiation | 13/134 | 107/7914 | 2.10933905937747e-07 | 13 |
| hsa05235 | PD-L1 expression and PD-1 checkpoint pathway in cancer | 12/134 | 89/7914 | 2.17784584411769e-07 | 12 |
| hsa05142 | Chagas disease (American trypanosomiasis) | 12/134 | 102/7914 | 9.83414056924318e-07 | 12 |
| hsa04660 | T cell receptor signaling pathway | 12/134 | 104/7914 | 1.17165864453336e-06 | 12 |
| hsa04145 | Phagosome | 14/134 | 152/7914 | 1.73782711388276e-06 | 14 |
| hsa04658 | Th1 and Th2 cell differentiation | 11/134 | 92/7914 | 2.53013590063901e-06 | 11 |
| hsa04151 | PI3K-Akt signaling pathway | 21/134 | 354/7914 | 2.89000392499786e-06 | 21 |
| hsa05150 | Staphylococcus aureus infection | 11/134 | 96/7914 | 3.63916138187669e-06 | 11 |
| hsa04672 | Intestinal immune network for IgA production | 8/134 | 49/7914 | 8.59947609525398e-06 | 8 |
| hsa04670 | Leukocyte transendothelial migration | 11/134 | 112/7914 | 1.60589932892716e-05 | 11 |
| hsa04630 | Jak-STAT signaling pathway | 13/134 | 162/7914 | 1.79630135972451e-05 | 13 |
| hsa05161 | Hepatitis B | 13/134 | 162/7914 | 1.79630135972451e-05 | 13 |
| hsa04657 | IL-17 signaling pathway | 10/134 | 93/7914 | 1.80385895462781e-05 | 10 |
| hsa04666 | Fc gamma R-mediated phagocytosis | 10/134 | 93/7914 | 1.80385895462781e-05 | 10 |
| hsa05134 | Legionellosis | 8/134 | 56/7914 | 2.03169917435232e-05 | 8 |
| hsa05332 | Graft-versus-host disease | 7/134 | 41/7914 | 2.38540787923219e-05 | 7 |
| hsa04610 | Complement and coagulation cascades | 9/134 | 79/7914 | 3.22796634654717e-05 | 9 |
| hsa05146 | Amoebiasis | 10/134 | 102/7914 | 3.7398928620283e-05 | 10 |
| hsa04625 | C-type lectin receptor signaling pathway | 10/134 | 104/7914 | 4.33893841498996e-05 | 10 |
| hsa05418 | Fluid shear stress and atherosclerosis | 11/134 | 139/7914 | 9.72649447667991e-05 | 11 |
| hsa05200 | Pathways in cancer | 23/134 | 530/7914 | 0.000105777615340188 | 23 |
| hsa05143 | African trypanosomiasis | 6/134 | 37/7914 | 0.000137102470868638 | 6 |
| hsa05133 | Pertussis | 8/134 | 76/7914 | 0.000165241733711088 | 8 |
| hsa05166 | Human T-cell leukemia virus 1 infection | 13/134 | 219/7914 | 0.000330205753090513 | 13 |
| hsa05221 | Acute myeloid leukemia | 7/134 | 67/7914 | 0.00051205835791603 | 7 |
| hsa05135 | Yersinia infection | 9/134 | 120/7914 | 0.000734884409613785 | 9 |
| hsa04015 | Rap1 signaling pathway | 12/134 | 210/7914 | 0.000839840517315461 | 12 |
| hsa04621 | NOD-like receptor signaling pathway | 11/134 | 181/7914 | 0.000891027357411082 | 11 |
| hsa05020 | Prion diseases | 5/134 | 35/7914 | 0.001029576982051 | 5 |
| hsa04217 | Necroptosis | 10/134 | 162/7914 | 0.00146029780524307 | 10 |
| hsa04010 | MAPK signaling pathway | 14/134 | 295/7914 | 0.00158536793677579 | 14 |
| hsa04066 | HIF-1 signaling pathway | 8/134 | 109/7914 | 0.00172532944087648 | 8 |
| hsa04014 | Ras signaling pathway | 12/134 | 232/7914 | 0.00185047338031372 | 12 |
| hsa05145 | Toxoplasmosis | 8/134 | 112/7914 | 0.00199312523462208 | 8 |
| hsa04810 | Regulation of actin cytoskeleton | 11/134 | 213/7914 | 0.00308839438201677 | 11 |
| hsa05167 | Kaposi sarcoma-associated herpesvirus infection | 10/134 | 186/7914 | 0.00382342612907088 | 10 |
| hsa05160 | Hepatitis C | 9/134 | 155/7914 | 0.0038720463391958 | 9 |
| hsa05163 | Human cytomegalovirus infection | 11/134 | 225/7914 | 0.0045710007706722 | 11 |
| hsa04612 | Antigen processing and presentation | 6/134 | 78/7914 | 0.00611832456663295 | 6 |
| hsa05130 | Pathogenic Escherichia coli infection | 10/134 | 201/7914 | 0.00637514632634908 | 10 |
| hsa05132 | Salmonella infection | 6/134 | 80/7914 | 0.00673558009425431 | 6 |
| hsa05205 | Proteoglycans in cancer | 10/134 | 204/7914 | 0.00687848089983006 | 10 |
| hsa04662 | B cell receptor signaling pathway | 6/134 | 82/7914 | 0.00739353954427867 | 6 |
| hsa05170 | Human immunodeficiency virus 1 infection | 10/134 | 212/7914 | 0.00881010520593022 | 10 |
| hsa04932 | Non-alcoholic fatty liver disease (NAFLD) | 8/134 | 149/7914 | 0.0101840990737672 | 8 |
| hsa05330 | Allograft rejection | 4/134 | 38/7914 | 0.0101840990737672 | 4 |
| hsa04611 | Platelet activation | 7/134 | 124/7914 | 0.0134263872921258 | 7 |
| hsa04664 | Fc epsilon RI signaling pathway | 5/134 | 68/7914 | 0.0152611411848263 | 5 |
| hsa04940 | Type I diabetes mellitus | 4/134 | 43/7914 | 0.0152611411848263 | 4 |
| hsa04622 | RIG-I-like receptor signaling pathway | 5/134 | 70/7914 | 0.0165275027620645 | 5 |
| hsa05120 | Epithelial cell signaling in Helicobacter pylori infection | 5/134 | 70/7914 | 0.0165275027620645 | 5 |
| hsa01521 | EGFR tyrosine kinase inhibitor resistance | 5/134 | 79/7914 | 0.0269084275363613 | 5 |
| hsa05320 | Autoimmune thyroid disease | 4/134 | 53/7914 | 0.0300695747942637 | 4 |
| hsa04072 | Phospholipase D signaling pathway | 7/134 | 148/7914 | 0.030944728498629 | 7 |
| hsa05202 | Transcriptional misregulation in cancer | 8/134 | 186/7914 | 0.0325434866452736 | 8 |
| hsa05310 | Asthma | 3/134 | 31/7914 | 0.0359504720149779 | 3 |
| hsa04370 | VEGF signaling pathway | 4/134 | 59/7914 | 0.0410097285084396 | 4 |
| hsa05416 | Viral myocarditis | 4/134 | 60/7914 | 0.0428211267811446 | 4 |
| hsa04623 | Cytosolic DNA-sensing pathway | 4/134 | 63/7914 | 0.0496921497045093 | 4 |
| hsa04068 | FoxO signaling pathway | 6/134 | 131/7914 | 0.0532571259387051 | 6 |
| hsa05165 | Human papillomavirus infection | 11/134 | 330/7914 | 0.0535244999643902 | 11 |
| hsa05322 | Systemic lupus erythematosus | 6/134 | 133/7914 | 0.0554815651697055 | 6 |
| hsa04210 | Apoptosis | 6/134 | 136/7914 | 0.0603252207602892 | 6 |
| hsa05230 | Central carbon metabolism in cancer | 4/134 | 69/7914 | 0.0628054591671527 | 4 |
| hsa04520 | Adherens junction | 4/134 | 71/7914 | 0.0679967402882973 | 4 |
| hsa05218 | Melanoma | 4/134 | 72/7914 | 0.0702560177093867 | 4 |
| hsa05212 | Pancreatic cancer | 4/134 | 75/7914 | 0.0788846778846886 | 4 |
| hsa05224 | Breast cancer | 6/134 | 147/7914 | 0.0788846778846886 | 6 |

Table S12 GO enrichment results between the MOD and YMPH groups for transcriptomics

| **term** | **enrichment** | **pvalue** | **count** | **class** |
| --- | --- | --- | --- | --- |
| immune system process | 0.848276 | 6.73048858631349e-72 | 123 | BP |
| immune response | 0.717241 | 2.85962828375142e-63 | 104 | BP |
| cell activation | 0.606897 | 1.09226246071705e-61 | 88 | BP |
| defense response | 0.648276 | 1.00086941287264e-60 | 94 | BP |
| cell surface receptor signaling pathway | 0.765517 | 1.08034223091034e-59 | 111 | BP |
| regulation of immune system process | 0.606897 | 7.64848584772782e-56 | 88 | BP |
| leukocyte activation | 0.544828 | 1.51705098572748e-54 | 79 | BP |
| response to cytokine | 0.517241 | 3.93660526310612e-50 | 75 | BP |
| immune effector process | 0.517241 | 2.4835626774674e-49 | 75 | BP |
| positive regulation of immune system process | 0.496552 | 1.09047180881263e-48 | 72 | BP |
| cell surface | 0.386207 | 5.33850882161665e-43 | 56 | CC |
| plasma membrane part | 0.6 | 2.37109852076188e-42 | 87 | CC |
| external side of plasma membrane | 0.241379 | 9.84345893585434e-38 | 35 | CC |
| side of membrane | 0.255172 | 4.46493602339326e-31 | 37 | CC |
| intrinsic component of plasma membrane | 0.372414 | 6.24369230252898e-27 | 54 | CC |
| integral component of plasma membrane | 0.344828 | 1.84785136949596e-24 | 50 | CC |
| receptor complex | 0.165517 | 6.15092261554753e-18 | 24 | CC |
| membrane raft | 0.165517 | 2.26753989473636e-17 | 24 | CC |
| membrane microdomain | 0.165517 | 2.44220850423961e-17 | 24 | CC |
| whole membrane | 0.331034 | 5.22826340038323e-17 | 48 | CC |
| signaling receptor binding | 0.386207 | 5.22826340038323e-17 | 56 | MF |
| signaling receptor activity | 0.372414 | 1.50612594581381e-24 | 54 | MF |
| molecular transducer activity | 0.372414 | 1.84282540392126e-23 | 54 | MF |
| cytokine receptor binding | 0.158621 | 6.67222881611053e-23 | 23 | MF |
| cytokine activity | 0.117241 | 2.86587401086667e-19 | 17 | MF |
| receptor regulator activity | 0.165517 | 1.51652336584996e-14 | 24 | MF |
| receptor ligand activity | 0.158621 | 1.65102660034644e-13 | 23 | MF |
| transmembrane signaling receptor activity | 0.255172 | 3.27058352003252e-13 | 37 | MF |
| cytokine binding | 0.089655 | 5.76458924323929e-13 | 13 | MF |
| CXCR3 chemokine receptor binding | 0.034483 | 2.58891048511208e-12 | 5 | MF |
